# Post-ingestion conversion of dietary indoles into anticancer agents

**DOI:** 10.1093/nsr/nwab144

**Published:** 2021-08-13

**Authors:** Li Ping Lin, Dan Liu, Jia Cheng Qian, Liang Wu, Quan Zhao, Ren Xiang Tan

**Affiliations:** State Key Laboratory of Pharmaceutical Biotechnology, Institute of Functional Biomolecules, Nanjing University, Nanjing 210023, China; State Key Laboratory Cultivation Base for TCM Quality and Efficacy, Nanjing University of Chinese Medicine, Nanjing 210023, China; State Key Laboratory Cultivation Base for TCM Quality and Efficacy, Nanjing University of Chinese Medicine, Nanjing 210023, China; State Key Laboratory Cultivation Base for TCM Quality and Efficacy, Nanjing University of Chinese Medicine, Nanjing 210023, China; State Key Laboratory Cultivation Base for TCM Quality and Efficacy, Nanjing University of Chinese Medicine, Nanjing 210023, China; State Key Laboratory of Pharmaceutical Biotechnology, Institute of Functional Biomolecules, Nanjing University, Nanjing 210023, China; State Key Laboratory of Pharmaceutical Biotechnology, Institute of Functional Biomolecules, Nanjing University, Nanjing 210023, China; State Key Laboratory Cultivation Base for TCM Quality and Efficacy, Nanjing University of Chinese Medicine, Nanjing 210023, China

**Keywords:** indole-3-carbinol, reaction flux derailing (RFD) approach, 2-(indol-3-ylmethyl)-3,3^′^-diindolylmethane (LTr1), anticancer, *Lactobacillus acidophilus*

## Abstract

There are health benefits from consuming cruciferous vegetables that release indole-3-carbinol (I3C), but the *in vivo* transformation of I3C-related indoles remains underinvestigated. Here we detail the post-ingestion conversion of I3C into antitumor agents, 2-(indol-3-ylmethyl)-3,3^′^-diindolylmethane (LTr1) and 3,3^′^-diindolylmethane (DIM), by conceptualizing and materializing the reaction flux derailing (RFD) approach as a means of unraveling these stepwise transformations to be non-enzymatic but pH-dependent and gut microbe-sensitive. In the upper (or acidic) gastrointestinal tract, LTr1 is generated through Michael addition of 3-methyleneindolium (3MI, derived *in situ* from I3C) to DIM produced from I3C via the formaldehyde-releasing (major) and CO_2_-liberating (minor) pathways. In the large intestine, ‘endogenous’ I3C and DIM can form, respectively, from couplings of formaldehyde with one and two molecules of indole (a tryptophan catabolite). Acid-producing gut bacteria such as *Lactobacillus acidophilus* facilitate the H^+^-promotable steps. This work updates our understanding of the merits of I3C consumption and identifies LTr1 as a drug candidate.

## Teaser

Reaction flux derailing (RFD) approach was conceptualized, established and showcased by its application in clarifying the conversion of dietary and endogenous indoles into anticancer agents.

## INTRODUCTION

Cruciferous vegetable intake is inversely associated with the risk of total mortality and thus has been conjectured to contribute to longevity [1]. Most crucifers biosynthesize indole glucosinolates, the breakdown product of which, indole-3-carbinol (I3C), has important roles in plant growth, development and chemical defense [2]. As a plant-derived small molecule, I3C regulates some biological processes in mammals and, particularly, exerts cancer-preventive action in diverse models [3,4]. However, I3C can be rapidly metabolized *in vivo*, and becomes undetectable in plasma within an hour after ingestion [5]. I3C metabolites identified *in vivo* include indole-3-carbaldehyde (I3A), indole-3-carboxylic acid (I3CA), indolo[3,2-*b*]carbazole (ICZ), 3,3^′^-diindolylmethane (DIM) and 2-(indol-3-ylmethyl)-3,3^′^-diindolylmethane (LTr1) [5] (Table S1). As a major I3C metabolite, DIM has been developed as a key ingredient of commercialized nutraceuticals with anticancer potential [6]. LTr1 has also been reported as a secondary metabolite of some marine bacteria such as *Psychrobacter* sp. [7]. In this work, LTr1 was shown to have superior antitumor efficacies over DIM in diverse mouse models and cell lines. These facts motivated us to question how I3C is metabolized *in vivo* and whether the health benefit of I3C intakes arises, at least partly, from I3C metabolites with antitumor action.

**Figure 1.**
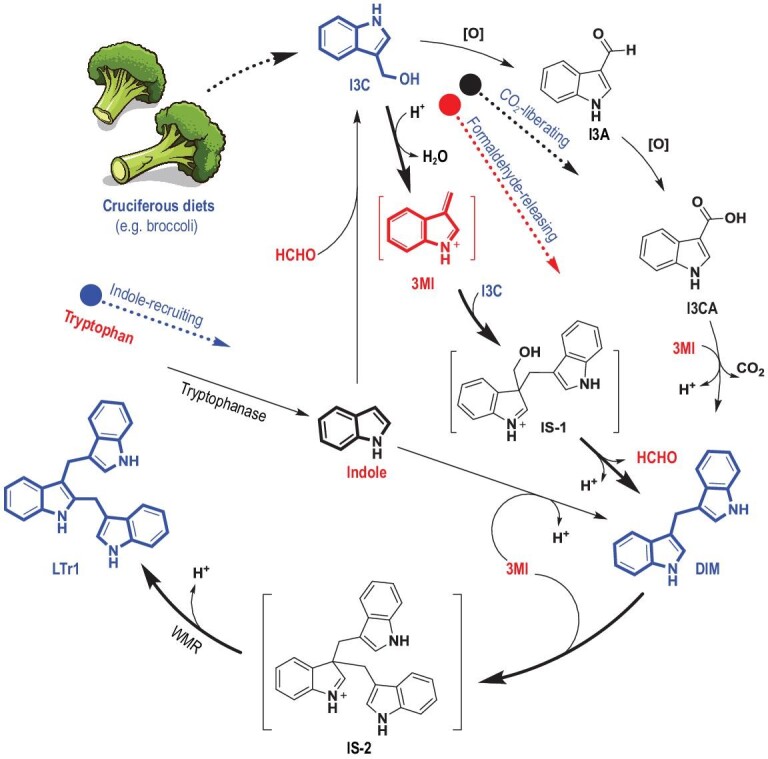
Three intertwined pathways for conversion of I3C to DIM and LTr1 in the gastrointestinal tract. The CO_2_-liberating pathway is characterized by decarboxylative Claisen condensation of I3C with its oxide I3CA. The formaldehyde-releasing pathway involves formaldehyde liberation from the I3C-coupled intermediate salt (IS-1). The indole-recruiting pathway occurs primarily in the large intestine where I3C and DIM form from couplings of formaldehyde with one and two indole molecules, respectively. I3C, indole-3-carbinol; I3A, indole-3-carbaldehyde; I3CA, indole-3-carboxylic acid; DIM, 3,3^′^-diindolylmethane; LTr1, 2-(indol-3-ylmethyl)-3,3^′^-diindolylmethane; 3MI, 3-methyleneindolium; IS-1 and IS-2, intermediate salts 1 and 2, respectively; WMR, Wagner–Meerwein rearrangement.

I3C is chemically labile and forms structurally diverse compounds in aqueous acidic media [8] and microbial culture [9,10]. Mammals secrete gastric juice and their gastrointestinal tracts harbor diverse microorganisms, some of which metabolize xenobiotics such as nutra- and pharmaceuticals [11,12]. But little is known about I3C transformation in the gastrointestinal tract and the contribution of such conversion to the cancer prevention resulting from I3C ingestion. Here we present that I3C, after ingestion, is converted successively to the anticancer agents DIM and LTr1, the latter being more effective in diverse cancer cell lines and various tumors in mouse models. The reaction flux derailing (RFD) approach was conceptualized and deployed to reveal the *in vivo* chemistry underlying the intertwined ‘indole→I3C→DIM→LTr1’ conversion chain (Fig. 1). The formaldehyde-releasing, CO_2_-liberating and indole-recruiting pathways, through which I3C is formed and transformed, were established to be non-enzymatic but pH-dependent, and thus accelerated by gastric acid and acid-producing gut bacteria such as *Lactobacillus acidophilus*. This work symbolizes a movement in understanding the health-beneficial or medical significance of consuming I3C or I3C-releasing diets and updates knowledge about the *in vivo* chemistry and biofunction of I3C and its metabolites such as LTr1 and DIM.

## RESULTS

### LTr1 is more effective than DIM in diverse cancer cell lines and tumor models in mice

There have been several studies on the antitumor potential of I3C and its metabolites, but it is thought that these have some inconsistencies in methodology and activity magnitude (Table S1). To compare the antitumor potency of I3C and its *in vivo* metabolites in the same experimental setting, a panel of cancer cell lines was used to evaluate the cytotoxicity of I3C, LTr1, DIM, I3A, I3CA and ICZ [5]. DIM was initially expected to be the most effective from its commercialization into nutraceuticals (e.g. DIM Supreme) and its inclusion in clinical trials as a tumor therapeutic candidate [6]. However, LTr1 was demonstrated to be about 10-fold more effective than DIM against growth of A549 and A375 cell lines with magnitude comparable to that of doxorubicin, a cancer therapeutic agent coassayed as a reference in the study (Table S2).

The shortage of treatments for lung cancer [11] motivated us to put top priority on investigating whether LTr1 is efficacious in A549-grafted nude mice. As illustrated in Fig. S1, the *in vivo* cancer inhibition by LTr1 was evidenced to be superior to that of DIM by the shrunken size and reduced weight of tumor tissues as well as lowered expression of Ki67 protein, a tumor proliferation marker [13]. With its efficacy ascertained in the tumor cell-grafted mice, we further tested whether LTr1 inhibits autochthonomous lung cancer. A mouse model of *Kras^G12D^*-driven lung tumorigenesis was developed according to the established protocol [14]. To our surprise, the superiority of LTr1 against the cancer over both DIM and pemetrexed disodium (a prescribed therapeutic for non-small cell lung cancer [15]) was corroborated by the lung morphology, microCT, hematoxylin-eosin (H&E) staining and Ki67 immunohistochemistry (Fig. S2). Furthermore, LTr1 was revealed to be well distributed in the autochthonomous lung cancer tissues (Fig. S3), indicating that it is stable *in vivo* and thus could be administered orally for cancer therapy.

### I3C turns into LTr1 via DIM in mouse gastrointestinal tract

**Figure 2.**
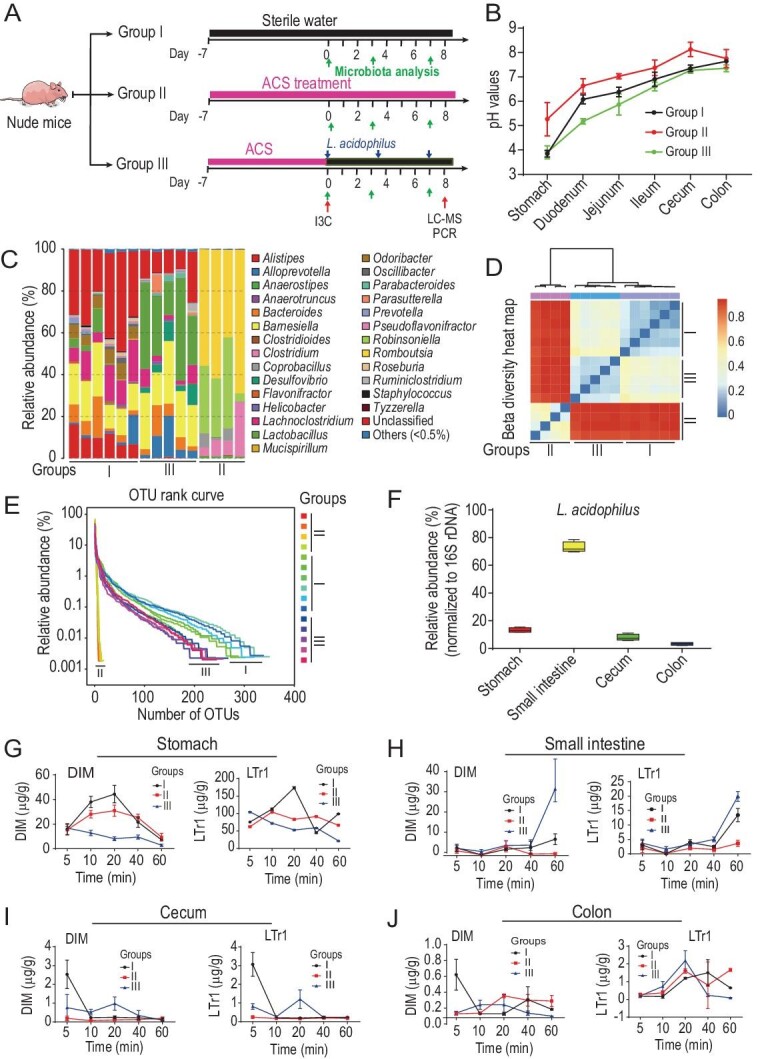
*Lactobacillus acidophilus* (*L. acidophilus*)-promoted conversion of I3C into LTr1. (A) Diagram of the treatment regimen and schedule. Four-week-old nude mice were randomized to three groups (*n* = 5). Group I received I3C ingestion only. Group II was pretreated with a mixture of broad-spectrum antibiotics (ampicillin + colistin + streptomycin (ACS)), followed by oral administration of I3C. Group III was successively ACS-decontaminated (first week), colonized with *L. acidophilus* and ingested with I3C (from the second week). (B) Deviated pH gradients measured for the gastrointestinal tracts of conventional (group I) and ACS-decontaminated mice without (group II, in red) and with *L. acidophilus* monocolonization (group III, in green). (C) The 16S rRNA-based quantitation of bacteria in mouse feces. Comparison between groups I and II confirmed the ACS-eradication of gut bacteria, and the difference between groups II and III indicated successful colonization of *L. acidophilus* in the ACS-decontaminated mice. (D) Beta diversity heat map based on the unweighted UniFrac distances using 16S rRNA analysis showed the similarity within, but significant difference among, the three treatments. (E) The inter-group difference in the operational taxonomic unit (OTU) rank curve demonstrated the robustness of both ACS-decontamination and *L. acidophilus* monocolonization. (F) The relative abundance (%) of *L. acidophilus* (normalized to 16S rDNA) in different gastrointestinal compartments of group III. The lines within boxes are medians, and the whiskers are the minimal and maximal values of the relative abundance (%) of *L. acidophilus*. Data were means ± SEM of four biological replicates. (G–J) Quantification of DIM and LTr1 from stomach (G), small intestine (H), cecum (I) and colon (J) in mice at indicated time points after the last administration of I3C as in (A). Data were means ± SEM of four biological replicates.

The promising anticancer activity of LTr1 intensified our interest in its *in vivo* formation from the ingested I3C molecule. We began by determining the pH gradient of the mouse gastrointestinal tract (Fig. 2B). Considering the UV-photolysis capable of generating indole radicals [16], we modeled the *in vivo* conversion of I3C by artificial gastric juice in a lightproof compartment. As shown in Fig. S4A, 10 min after exposure to the gastric juice, around 20% of I3C was converted to LTr1, and 5% to DIM. With the observation in mind, we examined the abundance variation of LTr1, DIM and I3C in the stomach contents sampled 10, 20, 40, and 60 min after the I3C ingestion (Fig. S4B). Comparable to the light-proofed test was the ‘I3C : DIM : LTr1’ ratio change (400 : 45 : 150 → 110 : 10 : 80) in the stomach from 10 to 60 min after the I3C ingestion (Fig. S4B).

If unconverted in the stomach, I3C might be transformed in the intestine. As expected, 60 min after the I3C gavage, the LTr1 content increased substantially in the small intestine (duodenum, jejunum and ileum) as evidenced by the ‘I3C : DIM : LTr1’ ratio changes in the duodenum (20 : 2 : 17 → 34 : 22 : 135), jejunum (18 : 6 : 40 → 28 : 16 : 105) and ileum (7 : 8 : 33 → 22 : 7 : 53) (Fig. S4B). In particular, LTr1 was (much) more abundant than DIM and I3C in these gastrointestinal compartments. But, in the large intestine (cecum and colon), I3C remained more detectable than were DIM and LTr1 (Fig. S4B). Such changes rationalized the LTr1 boost in the small intestine 60 min after the I3C gavage (Fig. S4B and 4C). This inspired us to investigate whether gut bacteria contribute to the I3C-to-LTr1 conversion. According to the described procedure [17], a panel of gut bacteria was isolated from human feces using the individualized cultivation protocol on diverse media (Table S3). The gut bacteria obtained were evaluated for their I3C metabolizing capacity, indicating that *Lactobacillus acidophilus* was the most efficient converter of I3C into LTr1 and DIM (Fig. S5). *Lactobacillus* strains consist of both true inhabitants and ingested probiotics and are highly populous throughout the human gastrointestinal tract [18–20]. Subsequent I3C conversion tests in mice were therefore performed with *L. acidophilus*, a representative of lactic acid bacteria residing in human and animal guts [19]. Following the described procedure [21], the nude mice were decontaminated by treatment with a mixture of broad-spectrum antibiotics [ampicillin + colistin + streptomycin (ACS)]. Ascertaining the successful ACS eradication of mouse gut microbes through 16S rRNA analysis, Petri dish culture, dilatation of caecum and H&E staining (Fig. S6), the ACS-decontaminated mice were randomized into two I3C gavage groups, one of which was monocolonized with *L. acidophilus* (Fig. 2). As indicated by the 16S rRNA, qPCR and LC-MS measurements, the *L. acidophilus* recolonized mice were disclosed to have an escalated bacterial abundance and an increased LTr1 level in the small intestine in comparison to the counterparts of those without the *L. acidophilus* monocolonization (Fig. 2F and H). As expected, such *L. acidophilus* monocolonization in the ACS-decontaminated mice acidified the gastrointestinal tract, as was evident from the lowered pH gradient of gastrointestinal compartments in comparison with counterparts in conventional and ACS-decontaminated animals (Fig. 2B). This set of experiments established the positive correlation of the LTr1 level with the *L. acidophilus* abundance and mouse gastrointestinal acidity, thereby signifying that the lactic acid bacteria can promote I3C-to-LTr1 transformation.

### Mechanism underlying the *L. acidophilus* promotion of the I3C-to-LTr1 conversion

**Figure 3.**
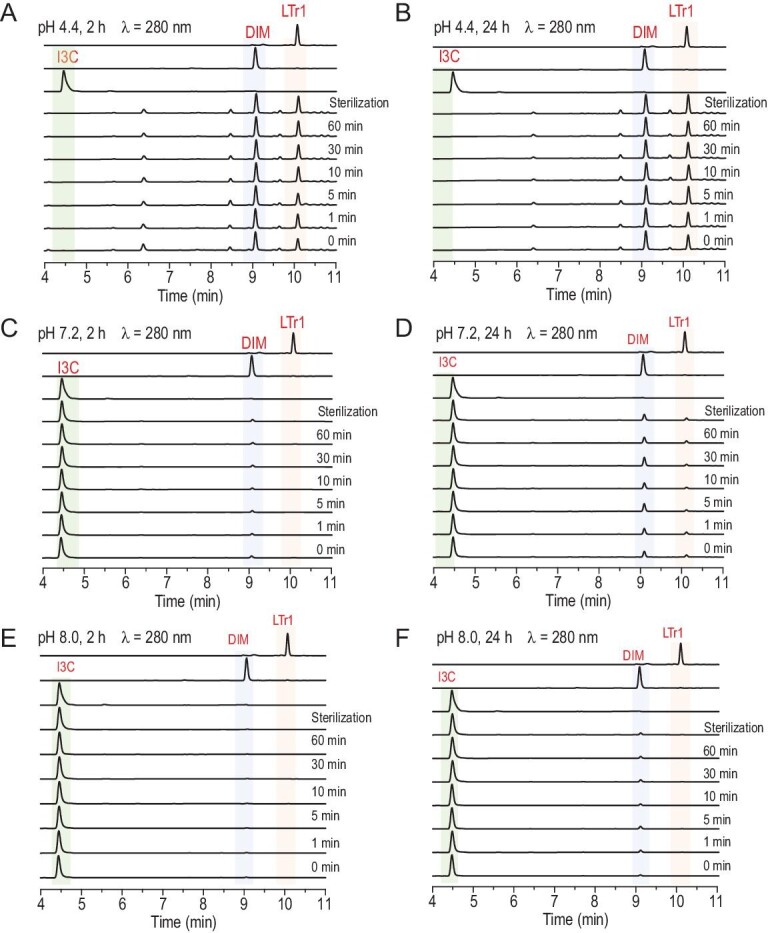
The pH- and time-dependent but non-enzymatic ‘I3C→DIM→LTr1’ transformation. (A–F) ‘I3C→DIM→LTr1’ conversion by *L. acidophilus*. With or without boiling (for 0, 1, 5, 10, 30 and 60 min, respectively) or sterilization (120°C, 30 min), the cell filtrates from *L. acidophilus* cultures were re-suspended in buffers at pHs 4.4, 7.2 and 8.0. Each obtained suspension was divided into two aliquots, which were stirred with I3C (0.15 mg/mL) at 37°C for 2 and 24 h, respectively, followed by LC-MS analysis.

Hypothetically, the I3C-to-LTr1 transformation by *L. acidophilus* could be enzymatic or non-enzymatic, or spontaneous but accelerated by enzymes as discerned with bacterial pericyclases we found recently [22]. To address the ambiguity, the conventional *L. acidophilus* culture was aliquoted into seven fractions for an inactivation test. The first six fractions were boiled for 0 (intact), 1, 5, 10, 30 and 60 min, respectively, so that the bacterial proteins therein were inactivated in ascending order. The seventh was autoclaved at 120°C for 30 min as a definite inactivation of bacterial proteins (Fig. 3). After the inactivation procedure, each of the seven fractions was centrifugated with the precipitate divided into three aliquots which were re-suspended by adding buffers up to the volume of the bacterial culture samples. The three suspensions derived from each precipitate were titrated to pHs 4.4, 7.2 and 8.0, respectively. Each of the titrated suspensions was aliquoted into two parts, which were stirred at 37°C with I3C for 2 and 24 h (Fig. 3A–F), respectively. Subsequent quantification showed that none of these protein inactivation procedures substantially affected transformation of I3C into DIM and LTr1, which, instead, were formed in a pH- and time-dependent manner (Fig. 3). These observations could be explained by assuming that the conversion of I3C into DIM and LTr1 was non-enzymatic but acidity-dependent. To reinforce the assumption, we tested for the formability of DIM and LTr1 from I3C in the blank MRS (de Man-Rogosa-Sharpe) medium used for culturing lactic acid bacteria. Surprisingly, LTr1 and DIM produced as well after supplementing I3C into the MRS medium, which was measured to have a pH of 5.6 after sterilization (Figs S5 and S7A). However, the LTr1 generation was fortified in the *L. acidophilus* culture, which was recorded to have a pH range of 4.0–4.5 (Fig. S7A). This suggested that the pH reduction by bacterially secreted lactic acid favored LTr1 generation. To explore the pH limit for I3C conversion, a series of transforming experiments were conducted in phosphate buffers at different pHs (Fig. S7B). In a basic pH (>7) range, DIM and LTr1 formed in reduced and negligible abundance (Fig. 3C–F), respectively. This is in line with the optimal pH (∼4) ascertained for the I3C conversion into LTr1 (Fig. S7A and 7B) and confirmed that the I3C-to-LTr1 transformation by *L. acidophilus* is non-enzymatic, but requires an acidic context (pH < 7). In addition, DIM and LTr1 were detectable in the cells derived from the I3C-exposed culture of *L. acidophilus*, but none of them could be detected in the conventionally cultured bacterial cells (Fig. S8). This observation underpinned that DIM and LTr1 are not bacterially produced but have their affinity to the bacterial cell membrane.

**Figure 4.**
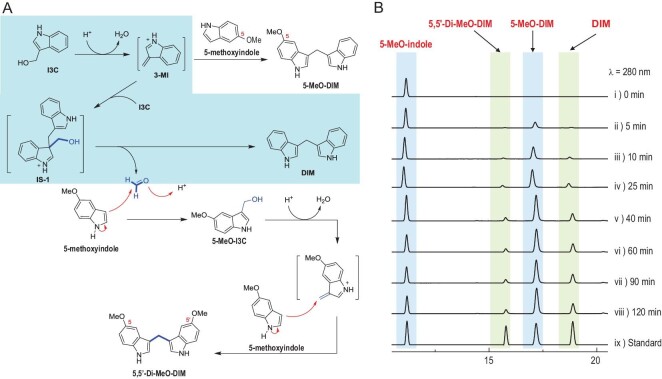
The reaction flux derailing (RFD) approach enabled capture of 3-methyleneindolium (3MI) and formaldehyde liberated from the I3C-to-DIM conversion. (A) The proposed mechanism underlying the incorporation of formaldehyde (liberated from IS-1, see Fig. 1) into 5,5^′^-Di-MeO-DIM, whereas 5-MeO-DIM resulted from compounding of 5-methoxyindole with 3-MI generated from I3C in methanol at pH 3–4. (B) HPLC detections of products obtained after stirring I3C and 5-MeO-indole under acidic conditions. 5,5^′^-Di-MeO-DIM, 5,5^′^-dimethoxylated DIM; 5-MeO-DIM, 5-methoxylated DIM.

The above observation aroused our curiosity about the pathways through which DIM and LTr1 are formed from I3C. Three decades ago [23], DIM was hypothesized to result from I3C dimerization in concert with formaldehyde elimination (hereafter referred to as the ‘formaldehyde-releasing’ pathway). But, to date, no experimental evidence has been found to support this assumption. To tackle the issue, we conceptualized, designed and deployed the reaction flux derailing (RFD) approach by taking advantage of the spontaneousness of the I3C-to-LTr1 conversion. The RFD methodology was created and expected to reroute or trap reaction intermediates using labeled co-substrates with identical or similar reactivities. As a RFD showcase, I3C was stirred in methanol at pH 4 with 5-methoxyindole under the N_2_-flow that prevented its air-oxidation. The 5-methoxy group of 5-methoxyindole, and other small-sized substituents on the benzene moiety of indoles used herein (*vide infra*), served as the ‘tag’ to indicate the substrate origination. Gratifyingly, the formaldehyde released from the I3C coupling into DIM was incorporated *in situ* into the predicted molecule (i.e. 5,5^′^-dimethoxylated DIM) with the ‘signature-like’ methylene motif in the middle of the molecule (Fig. 4). Such a RFD experiment afforded ‘5-methoxylated DIM’, too, highlighting the Michael addition of 5-methoxyindole (a nucleophile) to 3-methyleneindolium (3MI), which was formed as an electrophile from H^+^-promoted *in situ* dehydration of I3C and stabilized by its resonance with (indol-3-yl)methylium (Fig. 4 and Fig. S9). To confirm the 3MI origination, I3C was stirred with thioglycol in an acidic context to give 2-(3-indolylmethyl)thioethanol (Fig. S9A). Furthermore, the vulnerability of I3C to dehydration gained support from its ESI-MS spectrum where the most intense peak at *m/z* 130.0652 was interpreted to form from the elimination of water from the protonated I3C molecule (Fig. S9B). According to the RFD principle, other substituent-labeled indoles were tested and shown to trap the formaldehyde molecule liberated upon I3C-to-DIM transformation (Fig. S10), too. Collectively, the RFD approach established herein enabled capture of reactive intermediates and thus provided experimental evidence for DIM formation through the formaldehyde-releasing dimerization of I3C.

**Figure 5.**
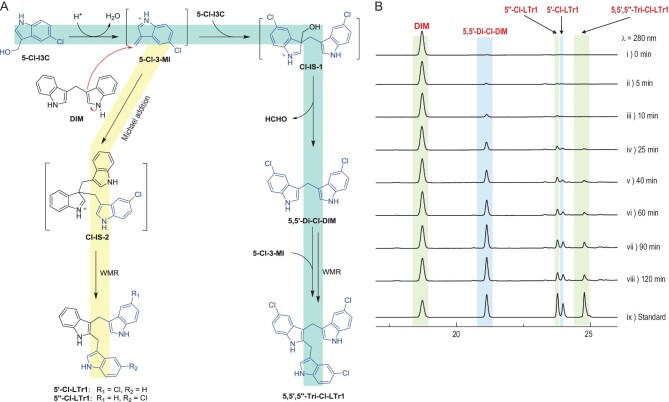
RFD-pinpointed stepwise progression of the Michael addition and Wagner–Meerwein rearrangement (WMR) reactions involved in I3C transformation into LTr1 via DIM. (A) The high 3-methylene electrophilicity of 5-Cl-3-MI (formed via H^+^-promoted dehydration of 5-Cl-I3C) allowed for its Michael addition with DIM to produce IS-2 (see Fig. 1), which underwent unbiased C_3_-to-C_2_ WMR migrations of 5-unsubstituted and 5-chlorinated (indol-3-yl)methyliums to afford 5^′^-Cl-LTr1 and 5^″^-Cl-LTr1, respectively. (B) HPLC monitoring of the reaction of DIM with 5-Cl-I3C to yield 5^′^-mono-, 5^″^-mono-, and 5,5^′^,5^″^-trichlorinated analogues of LTr1. 5-Cl-I3C, 5-chlorinated I3C; 5^′^-Cl-LTr1, 5^′^-chlorinated LTr1; 5^″^-Cl-LTr1, 5^″^-chlorinated LTr1; 5,5^′^,5^″^-Tri-Cl-LTr1, 5,5^′^,5^″^-trichlorinated LTr1; Cl-IS-1 and Cl-IS-2, chlorinated intermediate salts-1 and -2 (see Fig. 1), respectively.

LTr1 was hypothesized to be generated through the Michael addition of DIM with 3MI followed by a Wagner–Meerwein rearrangement (WMR) with proton abstraction (Fig. 1) [24], although this was unconfirmable prior to establishment of RFD. Herein, 5-Cl-I3C was selected and stirred with DIM under acidic conditions in view of the chlorine isotope abundance that eases the MS analysis of products. As anticipated, we obtained both 5^′^- and 5^′′^-chlorinated LTr1 analogues to underpin the Michael addition of DIM to 3-methylene-5-chloroindolium (5-Cl-3MI), which was generated *in situ* from 5-Cl-I3C as for I3C (Fig. 5 and Fig. S9). Such condensation was followed immediately by the WMR of one of the two similarly stabilized carbocations, (indol-3-yl)methylium and (5-chloro-indol-3-yl)methylium (Fig. 5). The reaction of 5-Cl-I3C with DIM also gave 5,5^′^-dichlorinated DIM (5,5^′^-di-Cl-DIM) and 5,5^′^,5^′′^-trichlorinated LTr1 (5,5^′^,5^′′^-tri-Cl-LTr1), highlighting that 5-Cl-I3C undergoes the same conversion to form these products as does I3C to yield LTr1 and DIM (Fig. 5). This is why the LTr1 generation from DIM is preconditioned by an acidic pH, at which 3MI and its congeners (e.g. 5-Cl-3MI) could form *in situ* from I3C and its variants such as 5-Cl-I3C, respectively. In alkaline media, 3MI is deprotonated into 3-methyleneindole whose 3-exomethylene electrophilicity is not sufficient for efficient addition to DIM (Fig. 4A and Fig. S9A). The rationalization agreed with the observation that, within a basic pH (>7) range, DIM and LTr1 formed from I3C in highly reduced and negligible yields (Fig. 3), respectively.

In air-exposed media, I3C can be spontaneously but slowly oxidized into I3CA [10,25]. In theory, DIM could be produced alternatively via decarboxylative Claisen condensation of I3CA with 3MI (formed *in situ* from I3C in an acidic context), termed the ‘CO_2_-liberating’ pathway (Fig. 1 and Fig. S11A). In an attempt to confirm this hypothesis, and if confirmed, to examine competitivity between the CO_2_-liberating and formaldehyde-releasing pathways, 5-methoxy-indole-3-carbinol (5-MeO-I3C) was subjected to RFD experimentation with exposure to I3CA at different pHs. According to the RFD principle, the expected 5,5´-diMeO- and 5-MeO-DIMs were reasoned to be the ‘signal products’ of the formaldehyde-releasing and CO_2_-liberating pathways (Fig. S11), respectively. At pHs 4 and 6, the anticipated products 5,5^′^-diMeO- and 5-MeO-DIMs formed, respectively, as major and minor products, signifying that the formaldehyde-releasing pathway was much more dominant. However, at pH 8, none of them could be detected in the reaction mixture (Fig. S11B). Accordingly, the two I3C-to-DIM transforming routes are active in acidic contexts, reaffirming that the H^+^-promoted dehydration of I3C into 3MI is the key step for formation of DIM in an acidic pH range. On the other hand, the oxygen level is quite low in mammal guts so that numerous anaerobes survive (Table S3). It is therefore reasonable for the CO_2_-liberating pathway to play a trivial role in transforming I3C into DIM in the mouse gastrointestinal tract. To probe whether the CO_2_-liberating and formaldehyde-releasing pathways are spontaneous, 5-MeO-I3C and I3CA were separately co-supplemented in the *L. acidophilus* culture and the blank MRS medium. Interestingly, both 5,5^′^-diMeO- and 5-MeO-DIMs were produced and no difference could be observed between the experiments using bacterial culture and MRS medium (Fig. S11C and 11D). However, 5,5^′^-diMeO-DIM was more abundant than 5-MeO-DIM, reaffirming the dominance of the formaldehyde-releasing pathway over the CO_2_-liberating one in the bacterial culture (Fig. S11C and 11D). A trace amount of 5,5^′^-diMeO- and 5-MeO-DIMs was detected at pH 7.4, at which 3-methyleneindole existed but underwent the Michael addition very slowly. Collectively, such experiments indicate that the DIM formation from I3C is spontaneous but pH-dependent.

### I3C and DIM form alternatively from endogenous indole and formaldehyde

**Figure 6.**
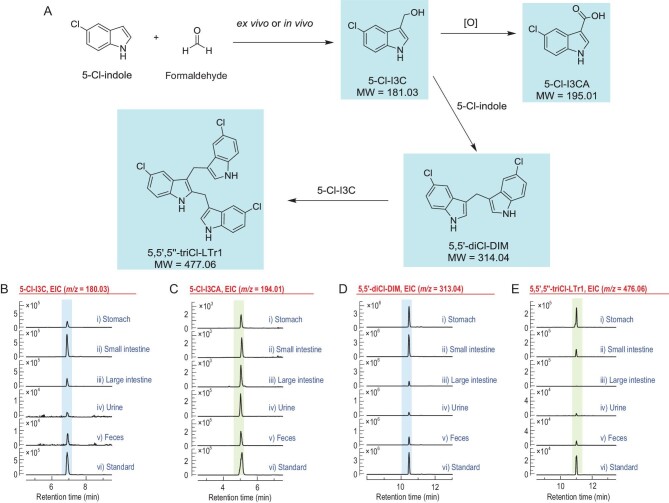
RFD-facilitated identification of the *ex vivo* and *in vivo* coupling of formaldehyde with indole into I3C, DIM and LTr1. (A) Scheme for *ex vivo* or *in vivo* compounding of 5-Cl-indole with formaldehyde into corresponding chlorinated analogues of I3C, I3CA, DIM and LTr1. (B–E) LC-MS detection of the chlorinated analogues in stomach and intestine as well as in urine and feces. (B–E) The EICs corresponded to 5-Cl-I3C (*m/z* 180.03, [M–H]^–^) (B), 5-Cl-I3CA (*m/z* 194.01, [M–H]^–^) (C), 5,5^′^-diCl-DIM (*m/z* 313.04, [M–H]^–^) (D) and 5,5^′^,5^″^-triCl-LTr1 (*m/z* 476.06, [M–H]^–^) (E).

The aforementioned findings were solid enough to explain production of DIM and LTr1 in the upper gastrointestinal tract (stomach and small intestine), which is acidic (Fig. 2B). But, to our surprise, DIM was also detected in the large intestine (Fig. 6D), perhaps formed via yet-to-be-defined pathway(s). In healthy individuals, formaldehyde distributes intracellularly at 0.2–0.5 mM as a common metabolite of methanol, creatine and folate [26], and indole exists in human plasma (4.5–9.4 μg L^−1^) [27] and feces (0.25–1.2 mM) [28] as a tryptophan catabolite [29]. Also encouraged by the formation of 5,5^′^-dimethoxylated DIM from addition of formaldehyde to 5-methoxyindole (Fig. 4), these reports motivated us to hypothesize that indole and formaldehyde might react to form I3C and/or DIM in their physiological concentration ranges (we thus termed such a route the ‘indole-recruiting’ pathway). To corroborate the assumption, indole (0.25 mM) and formaldehyde (0.2 mM), both being below the physiological concentration maxima, were stirred at 37°C at pH 8 in a sealed tube. Subsequent LC-MS analysis indicated that I3C and DIM were generated in a reaction time-dependent manner (Fig. S12B and 12C). This observation could be explained by assuming the base-promoted Friedel–Crafts reaction of indole with formaldehyde to form I3C, and ‘endogenous’ I3C thus formed might undergo a bimolecular nucleophilic substitution (SN2) reaction with indole at its hydroxymethyl carbon to yield DIM (Fig. S13). To ascertain the acidity- and enzyme-dependence of the coupling of indole with formaldehyde, the *L. acidophilus* cells were collected and aliquoted into two parts, one of which was autoclaved at 120°C for 30 min. After such pretreatment, each of the two parts was divided into three aliquots which were re-suspended in buffers at pHs 4.4, 7.2 and 8.0, respectively. Each suspension was divided into two aliquots, in which indole and formaldehyde were agitated at 37°C for 2 and 24 h, respectively (Fig. S12D and 12E). Interestingly, I3C and DIM were both detectable at pH > 7.0 in both living and autoclaved *L. acidophilus* cultures. However, at acidic pHs, I3C disappeared and DIM was concentrated presumably because of H^+^-promoted I3C-to-DIM conversion (Fig. S12D and 12E). The observation highlighted that coupling of indole to formaldehyde was spontaneous but pH- and time-dependent (Fig. S12D and 12E). Moreover, the indole-formaldehyde coupling seems to be irreversible as we failed to detect any products that might result from the retro-aldol reaction of I3C (Fig. S14).

To ascertain the generalizability of the base-promoted condensation of indole with I3C, indole was individually stirred at pH 8 with 5-chlorinated and 5-methoxylated derivatives of I3C to yield the corresponding 5-chloro- and 5-methoxy-DIMs (Fig. S13B). Analogously, I3C was separately mixed at pH 8 with each of 5-chlorinated, 5-methoxylated, N-benzylated and N-methylated indoles to afford 5-chloro-, 5-methoxy-, N-benzyl- and N-methyl-DIMs (Figs S13B, S35−42), respectively. Such a base-promoted condensation of indole with I3C analogues explained why 5-MeO-I3C reacted with indole to give 5-MeO-DIM (major) and 5,5^′^-diMeO-DIM (minor) at pH 8.0 (Fig. S13B and S15). However, at basic pHs, LTr1 failed to form from the condensation of I3C with DIM (as a 3-substituted indole), reaffirming that the LTr1 generation could only be realized through the 3MI-mediated route in an acidic context (Figs 3 and 5). Similarly, MRS media with or without *L. acidophilus* were separately exposed to 5-MeO-I3C and indole. As illustrated in Fig. S15, 5-MeO-DIM was much more abundant than 5,5´-diMeO-DIM, indicating the dominance of the indole-recruiting pathway over the formaldehyde-liberating one.

The above findings motivated us to confirm DIM formation in the large intestine by *in vivo* and *ex vivo* experiments. Mice were gavaged with formaldehyde and 5-chloroindole (chlorine group as tag) at doses within their physiological concentration ranges. As presumed, in mouse feces and urine, 5,5^′^-diCl-DIM and 5,5^′^,5^″^-triCl-LTr1 appeared in an approximate 100 : 1 ratio (Fig. 6). However, 5,5^′^-diCl-DIM, but not 5,5^′^,5^″^-triCl-LTr1, was detected in mouse large intestine (Fig. 6). In alignment with the observation, both 5,5^′^-diCl-DIM and 5,5^′^,5^″^-triCl-LTr1 were generated by equally performed *ex vivo* experiments using mouse stomach and small intestine (Fig. 6). This reinforces that acidity is essential for LTr1 generation from DIM, although formaldehyde and indole used herein for experimentation were a bit more concentrated than their ordinary physiological level. Collectively, I3C and DIM can form alternatively from endogenous indole and formaldehyde, which are more abundant in the lower gastrointestinal tract [26,28,30].

## DISCUSSION

This work shows that the H^+^-facilitated transformation of I3C into LTr1 via DIM can be realized in the upper gastrointestinal tract of mice through the formaldehyde-releasing (major) and CO_2_-liberating (minor) pathways. The indole-recruiting pathway exists in the lower mouse gastrointestinal tract, and allows for ‘endogenous I3C’ formation via the addition reaction of indole (a tryptophan catabolite [29]) to formaldehyde, a common metabolite of methanol, creatine and folate [26]. Endogenous I3C thus formed can be further transformed into DIM, but not LTr1, through its coupling with indole. Concerning the mechanism, the entire ‘indole→I3C→DIM→LTr1’ conversion is non-enzymatic but pH-sensitive, and thus depends on the gastrointestinal compartments. In the stomach and small intestine, LTr1 is produced from the Michael addition of DIM with 3MI generated *in situ* from H^+^-promoted dehydration of I3C. In the large intestine where the pH is around 8 (Fig. 2B), LTr1 production appears negligible, but DIM forms alternatively from the SN2 reaction of indole with I3C produced from the Friedel–Crafts reaction of formaldehyde to indole (Figs S12 and S13). This explains why acid-producing bacteria such as *L. acidophilus* can escalate the transformation of I3C into LTr1 via DIM in the upper mouse gastrointestinal tract. Because of dependence on abiotic factors such as pH, substrate concentration and interaction time, the formaldehyde-releasing, CO_2_-liberating and indole-recruiting pathways established herein (Fig. 1) may be involved in conversion of indole(-coined) compounds by most (if not all) domains of life.

Almost all metazoan taxa harbor a vast ensemble of microbes, but pH seems to be a versatile determinant of the microbiome stratification in a particular niche [31]. Our gastrointestinal compartments are pH-differentiated for optimum digestion of foods and killing of ingested pathogens [32], and this is why many gut microbes (e.g. *L. acidophilus*) produce pH-titrating acids to outcompete each other in diverse host niches [33,34]. This study assigns the driving force behind conversion of I3C into DIM and LTr1. In the gut, DIM is generated mainly via the formaldehyde-releasing pathway, although, alternatively, it could be produced by indole-I3C coupling in the large intestine. However, LTr1 is only formed from I3C in acidic conditions that allow for formation of the key protonated intermediate IS-2 (Fig. 1). This explains why the I3C-to-LTr1 conversion via DIM is non-enzymatic but depends on the acidity of particular gastrointestinal compartments. Interestingly, such conversion proceeds spontaneously in mouse intestines. Our protein inactivation experiments showed that no enzyme may accelerate the conversion. As enzymatic and spontaneous conversions follow identical rules of chemistry [35,36], we took advantage of this non-enzymatic feature to create a reaction flux derailing (RFD) strategy to decipher the transformation mechanism. The RFD methodology can be generalized and practiced easily by adopting substituent-labeled co-substrates with similar reactivity to competent capturing agents (Figs 4–6 and Figs S10, S11, S13 and S15). The prowess of the RFD approach was showcased by, but not limited to, our RFD-facilitated confirmation of the acid-triggered/promoted reactions involved in the I3C conversion to antitumor agents LTr1 and DIM (e.g. SN2 reaction, Lewis-acid-promoted dehydration, Michael addition, Friedel–Crafts reaction and Wagner–Meerwein rearrangement). Using RFD, we were able to reshape the function of (highly) acidic gastrointestinal compartments in chimerizing some pH-susceptible xenobiotics into functionally and architecturally distinct molecules. Coincidently, an acid production strategy has been adopted to counteract the plant-defensive effect of I3C by some crucifer pathogenic microbes such as *Sclerotinia sclerotiorum* [37], an efficient I3C metabolizer capable of producing oxalic acid [10]. It is also noteworthy that the pH-dependence strategy is used in nature by some microbes for constructing skeletally disparate natural products such as merochlorin antibiotics [35]. From an evolutionary viewpoint, pH stratification in the mammal gut could be a general strategy by which different organisms survive in nature.

The origin of health benefits from consumption of I3C or I3C-containing cruciferous vegetables is a long-standing issue because of I3C lability [5,38]. As shown by the present investigation, it is the I3C lability that leverages and allows for its *in vivo* transformation into DIM and LTr1. In particular, LTr1 is prominent in cancer prevention with its *in vivo* efficacy higher than, or at least comparable to, that of DIM which has been approved as a nutraceutical during its clinical trial as a tumor therapeutic candidate [6]. Therefore, it is reasonable to conclude that LTr1 and DIM contribute to the anticancer efficacy resulting from the I3C intakes. Concerning the mode of action of anticancer indoles, I3C and DIM function as aryl hydrocarbon receptor ligands [39]. DIM was additionally reported to induce endoplasmic reticulum stress and modulate miRNA expression [40]. LTr1 was found to regulate the activity (or expression) of quinone reductase [41], cytochrome P450 and phase II enzymes [42]. These reports highlight that, despite the structural comparability, LTr1 might differ from DIM and I3C in its pharmacological mechanism, which falls beyond the scope of the work. In correlation with the I3C lability *in vivo* [5] and *in vitro* [8–10], the observation raises the possibility that many bioactivities ascribed to I3C might be due to its metabolites such as DIM and LTr1.

This work identifies the endogenous production of I3C from the Friedel–Crafts addition of indole to formaldehyde in the large intestine (Figs 1, 6 and S12), being distinct from I3C generation from skatole (3-methylindole) in animals [43,44]. Starting from this finding, the investigation identifies a novel endogenous route to DIM consisting of base-facilitated steps : (i) the *in situ* synthesis of I3C from the indole-formaldehyde coupling and (ii) the SN2 reaction of thus formed I3C with indole (Fig. 1 and Fig. S13). In view of the origination of indole and formaldehyde from ingested diets [26–28,30], the aforementioned findings add a new layer of mechanistic complexity to the metabolism of I3C-related indoles in biosystems, and in particular, further understanding of the health-beneficial basis of consuming suitably combined foods. In this sense, the *in vivo* chemistry clarified herein for indoles may serve as an exemplification of a valid model of investigating diverse chemicals we ingest every day.

In conclusion, the work identifies LTr1 as a promising anticancer drug candidate under the inspiration of the health benefits resulting from consumption of cruciferous vegetables that liberate I3C while chopped and processed. The stepwise conversion of I3C into LTr1 via DIM has been addressed by conceptualizing, designing and utilizing the easy-to-follow RFD approach that enabled our establishment of the formaldehyde-releasing, CO_2_-liberating and indole-recruiting pathways. In view of the involvement of Lewis acid/base chemistry in many biological reactions [36], it can be anticipated that more arrays of health-benefiting compounds can form from diversely structured pH-susceptible chemicals in diets and orally given ethnomedicines, in acidic (micro)environments inside the gastrointestinal compartments including the large intestine [45]. While reminding us to reconsider the actual contributor to the reported biological action of I3C in terms of *in vivo* chemistry, the study raises a few interesting topics such as the anticancer spectrum and mechanistic aspect of LTr1 as well as the role of the unique pH stratification of our gastrointestinal tracts that sense and process countless xenobiotics with diverse structures.

## METHODS

Detailed information about cell lines, culture conditions, proliferation assay, treatments and sample collection, X-ray microCT scanning, pharmacokinetics, chromatographic and mass spectrometric analysis, immunohistochemistry (H&E and Ki67), conversion of I3C into LTr1 *in vitro* and *in vivo*, screening for the I3C metabolizer from human fecal bacteria, monocolonization of antibiotic-decontaminated mice with *L. acidophilus*, 16S rRNA sequencing and data analysis, qPCR, antibiotic treatments for gut bacterial eradication and chemical synthesis are included in the supplementary data online. Variable regions V3–V4 of bacterial 16S rRNA gene were amplified with degenerate PCR primers and the raw data are available.

## DATA AVAILABILITY

The data underlying this article are available in the National Center for Biotechnology Information (NCBI) Gene Expression Omnibus (GEO) database, and can be accessed with submission number PRJNA657969. Crystallographic data of LTr1 in CIF format are available in the Cambridge Crystallographic Data Centre, and can be accessed with no. CCDC-1499138.

## Supplementary Material

nwab144_Supplemental_File
