# Supplementary material for: Post-ingestion conversion of dietary indoles into anticancer agents
Source: Natl Sci Rev. 2021 Aug 13;9(4):nwab144. doi: 10.1093/nsr/nwab144 (PMC9053945; doi:10.1093/nsr/nwab144)
Supplement: nwab144_Supplemental_File [file nwab144_supplemental_file.pdf]

Supplementary Information

Post-ingestion conversion of dietary indoles into anticancer agents

Li Ping Lin,[1,2‡] Dan Liu,[2‡] Jia Cheng Qian,[2] Liang Wu,[2] Quan Zhao,[1] Ren Xiang Tan[1,2*]

[1]State Key Laboratory of Pharmaceutical Biotechnology, Institute of Functional Biomolecules, Nanjing University, Nanjing 210023, P. R. China
[2]State Key Laboratory Cultivation Base for TCM Quality and Efficacy, Nanjing University of Chinese Medicine, Nanjing 210023, P. R. China
*Corresponding author. E-mail: rxtan@nju.edu.cn
‡Contributed equally to this work.

The authors declare no conflict of interest.

This PDF file includes:

Supplementary text

Tables S1 to S3

Figs. S1 to S61

SI References

Supplementary Information Text

Methods

Animals. The animal welfare and experimental procedures were carried out in accordance with the regulation of animal experimentation approved by the Animal Care and Use Committees of Nanjing University and Nanjing University of Chinese Medicine. Unless stated otherwise, all animals were housed at 20 ± 2 °C in the specific-pathogen-free (SPF) condition with relative humidity of 50 ± 10% and a strict 12 h light/dark cycle with free access to sterile chaw and water. Mice used were 4/5-week-old. Institute of Cancer Research (ICR) mice were purchased from Qinglongshan Experimental Animal Breeding Farm (Nanjing, China), and the nude mice from Model Animal Research Center of Nanjing University (Nanjing, China). The C57BL/6 *Kras*$^{G12D/+}$ mice were obtained from the Jackson Laboratory, USA. For the *in vivo* studies, animals were randomized to different treatments, and investigators were blinded to

allocation during experiments and outcome assessment.

Xenograft tumor model. The A549 suspension (6 × 10^6 in 100 μl) in phosphate buffer saline (PBS) was separately injected subcutaneously into the right lower flank of female nude mice. As described (1), all test animals were monitored, every three days, for vitality, physiological condition, body weight and tumor growth. The tumor volume was calculated using $^1/_2 \times L \times W^2$, where '*L*' and '*W*' are the long and short diameters (in cm), respectively. When the grafted tumor sized 20~40 mm^2 (designated as "day 0"), the mice were randomized to treatment and control groups (Fig. S1).

Kras$^{G12D/+}$ **mice and adenoviral infection. The** C57BL/6 *Kras*$^{G12D/+}$ mice were authenticated as reported (2) using the following primers: forward primer for wild mice, 5′-TGTCTTTCCCCAGCACAGT-3′; for mutant mice, 5′-GCAGGTCG AGGGACCTAATA-3′; and reverse 5′-CTGCATAGTACGCTATACCCTGT-3. Endogenous lung tumors were generated in the *Kras*$^{G12D/+}$ mice through the intranasal administration of 15 μl of 2.7 × 10^7 μg/ml AdCre virus (pAAV-CMV bGlobin-Cre-eGFP, Shanghai Genechem Co., Ltd, China) after anesthetized by intraperitoneal injection of 100 μl of 8% chloral hydrate (3). After a 13-week gavage of test samples, the responses were evaluated by CT image analysis. Lungs were collected 15 min after the last treatment and formalin fixation (Fig. S2).

Cell lines and cell culture. Human lung (A549), colon (SW480), liver (HepG2), melanoma (A375), breast (MCF-7) and ovarian (Caov-3) cell lines (purchased from Shanghai Zhongqiao Xinzhou Biotechnology Co., Ltd., China) were authenticated by the routine short tandem repeat profiling. After ascertained to be free of mycoplasma contamination, all cells were cultured at 37 °C under 5% CO_2 in Dul-becco's modified Eagle's medium (DMEM) supplemented with 10% fetal bovine serum (FBS), 100 units/l penicillin G sodium and 100 μg/l streptomycin sulfate. All reagents were obtained from Gibco-Invitrogen, Carlsbad, CA, USA.

MTT cell proliferation assay. The cytotoxicity was evaluated against the cell lines as described (1). Briefly, the cancer cells at the exponential growth phase were transferred into 96-well plates at an approximate density of 1 × 10^4/well and incubated for 24 h. All test compounds were dissolved in DMSO at a final concentration of 10 mM as stock solution, followed by diluting with DMEM (Dulbecco's modification of Eagle's medium) to various

concentrations and added into quintuplicate wells with the vehicle (0.1% DMSO) allocated as a blank control. After a 48-hour incubation, 20 μl MTT (3-(4,5-dimethyl-2-thiazolyl)-2,5-diphenyl-2-*H*-tetrazolium bromide) solution (0.1 mg per well) was added to each well, followed by the incubation for the ensuing 4 h. The supernatant was removed to give the crystals which were fully dissolved in DMSO (100 μl) with the absorbance of each well measured at 490 nm (Sunrise, Tecan). The half maximal inhibitory concentration (IC_{50}) was determined as the concentration, at which a half of cell growth was inhibited. The experiment was performed in triplicate, and the data expressed as means ± SD (Table S2).

***In vivo* evaluation of I3C, DIM and LTr1.**

As detailed in Figs. S1 and S2.

X-ray microCT scanning for lung tumor. As described (3), the C57BL/6 $Kras^{G12D/+}$ mice were anaesthetized using isoflurane and fixed under anaesthesia during the entire scanning procedure. Lung image acquisition was performed on high-resolution X-ray microCT scanning (Quantum FX; PerkinElmer, Hopkinton, MA, USA). X-ray source was set to a current of 40 μA and a voltage of 70 kV. The scan was initiated from high resolution scan mode and lasted for 14 min per mouse. CT images were taken with a field of view of 36 mm ×36 mm and a voxel size of 72 μm (Fig. S2) (4).

Scanning H&E and Ki67 slides, ImageJ and GraphPad.

H&E and Ki67 sections were scanned with Olympus CX41 biomicroscope (Olympus optics Co., Ltd., Japan) and Olympus dp72 micro imaging system (Olympus optical industry Co., Ltd., Japan) and read by Pannoramic MIDI digital slice scanning system (3DHISTECH, Hungary). Quantifications were performed using ImageJ (Version 1.52v). Statistical analysis was performed in GraphPad. One point indicates an average of five FoVs per tumor from a mouse. For comparison, quantification of histological markers was only performed on tumor sections stained at the same time. For proliferation marker Ki67 the immunohistochemistry staining was quantified as the percentage of total cells per Field of View using ImageJ (Fig. S1 and S2).

Analysis of I3C, DIM and LTr1.

Plasma and tissues from animals. Mice were gavaged with I3C at the dosage of 150 mg/kg,

and after certain time duration, were executed to collect blood, stomach, duodenum, jejunum, ileum, cecum and colon tissues. Blood was centrifuged at 8,000 rpm for 10 min to obtain plasma. Tissues taken from tested mice were chopped, accurately weighed, and grinded into homogenates with liquid nitrogen in deionized water, respectively.

Sample preparation. Plasma and homogenate samples (50 or 100 μl) were extracted by 5-fold volumes of ethyl acetate containing ketoconazole 50 ng l^{-1} for concentration determination of I3C, DIM and LTr1 in plasma and tissues from mice as internal standard. The mixture was vortex-mixed for 3 min and then centrifuged at 8,000 rpm for 10 min. The supernatant (200 or 450 μl) was transferred and evaporated to a dryness under vacuum at 30 °C. The residues were re-suspended in 200 μl of methanol and centrifugated at 13,000 rpm for 10 min twice. A 2-μl aliquot of the supernatant from each sample was injected for the LC-MS/MS analysis.

Calibration curve and quantification. Working solutions of LTr1 with concentrations in the range of 1–200 μg/ml were obtained by serially diluting the stock solution with methanol. All solutions were stored at 4 °C and brought to room temperature before use. The calibration standards were prepared by spiking the series of working solutions (2.5 or 5 μl) into blank plasma or tissues (47.5 or 95 μl) to yield LTr1 concentrations of 0.005, 0.05, 0.1, 0.25, 0.5, 1, 2.5, 5 and 10 μg/ml.

Chromatographic and mass spectrometric analysis

Chromatography condition. The chromatographic separation was carried out using an Agilent Zorbax Eclipse Plus C18, 2.1 × 100 mm i.d., 3.5-μm column (Agilent Technologies, USA), on a Shimadzu LC-30A UFLC system (Shimadzu, Kyoto, Japan). The column oven temperature was set at 35 °C. A mobile phase consisting of 0.025% formic acid in water (A) and methanol (B) was delivered at a flow rate of 0.2 ml/min using the following gradient program: 5% (B) from 0→5 min, 5-60% (B) from 5→12 min, 60-80% (B) from 12→21 min, 80% (B) from 21→22 min, 80-95% (B) from 22→22.5 min, 95% (B) from 22.5→24 min, 95-5% (B) from 24→24.5 min and 5% (B) from 24.5→28.5 min for concentration determination of I3C, DIM and LTr1 in plasma and tissues from animals.

Mass spectrometry conditions. Quantitation were performed by a LC-MS/MS system (Triple Quad 4500, Applied Biosystems, MDS SCIEX, Toronto, Canada), which including an Analyst

software (Version 1.6.1) and a MultiQuant software (Version 2.1). The mass operation parameters were set as follows: a positive TurbooSpray ion source was used; ion source gas 1 (GS1) was set at 40 psi, ion source gas 2 (GS2) at 50 psi, curtain gas (CUR) at 40 psi, collision gas (CAD) at medium, temperature at 550 °C, and IonSpray voltage floating (ISVF) at 4500 V. All the compounds were monitored via multiple reaction monitoring (MRM). The precursor-to-product ion pairs for MRM transitions, DP and CE were optimized at *m/z* 130.0 → 103.0, 55 V and 30 V for I3C, 247.1 → 130.0, 35 V and 17 V for DIM, 376.1 → 247.1, 40 V and 12 V for LTr1, and 531.2 → 489.1, 90 V and 42 V for IS, respectively (Fig. 2, Fig. S3 and S4).

Conversion of I3C into LTr1 by gastric juice. To simulate its conversion *in vivo*, I3C was dissolved at 0.15 mg/l in the artificial intestinal juice (pH = 1.2) in a lightproof compartment followed by incubation at 37 ºC with an agitation of 200 rpm. The LC-MS quantification was performed for the DIM and LTr1 abundance in the I3C-added gastric juice sampled 10, 20, 40 and 60 min after the incubation started (Fig.S4A).

Conversion of I3C into LTr1 *in vivo*. After fasting for 24 h, mice were gavaged with I3C at 250 mg/kg. The blood, stomach, duodenum, jejunum, ileum, cecum and colon samples were taken from rats 10, 20, 40 and 60 min after the I3C administration (the duodenum, jejunum and ileum of mice were dissected together as “small intestine”). The tissues were homogenized to obtain the corresponding homogenates. All samples were stored at –80 °C prior to use (Fig. 2 and S4).

Screening for the I3C metabolizer from human fecal bacteria. An anaerobic facility (Whitley DG250 Anaerobic Workstation, United Kingdom) containing 20% CO_2, 10% H_2 and 70% N_2 was used for all anaerobic cultivations. As detailed (5), fresh human feces were inoculated into test tubes containing 10 ml of one of the five media adopted in the study (GAM, BS, MRS, LB and brain-heart infusion (BHI, Hopebio, Qingdao, China); the latter three with 10% horse blood (Quad Five)). The bacterial incubation was performed at 37 °C for 24 h under aerobic or anaerobic conditions (Table S3). The incubated liquid cultures were spread on agar plates, and the bacteria that grew were isolated. As described (6), after separately incubating at 37 °C for 24 h under anaerobic or aerobic conditions, 19 human fecal bacteria were harvested and resuspended in sterile relevant medium at an optical density (600 nm) of 2.50. Individually, 10 ml of such medium was exposed to I3C (0.05 %) and incubated for

another 24 h under the same conditions (Table S3). I3C conversion assays were determined by HPLC (Fig. S5).

Antibiotic treatments for gut bacterial eradication. All antibiotics were obtained from Sigma Aldrich (USA). The female nude mice were given the sterile drinking water containing ampicillin (1 mg/ml), colistin (1 mg/ml), and streptomycin (5 mg/ml) (abbreviated as "ACS") as described (Fig. 2) (1). The effectiveness of the antibiotic-treated murine model (also called bacteria-depleted mice) was confirmed by 16S rRNA and/or qPCR analysis, along with the dilated caecum, hematoxylin-eosin (H&E) staining profiles of cecum, and the colony density of the cultivable bacteria from mouse feces according to the established protocol (Figs. 2C—2F and Fig. S6) (1).

Monocolonizaion of antibiotic-treated mice with *L. acidophilus*. As detailed (7), 10 ml of *L. acidophilus* culture under anaerobic condition at 37 ℃ for 24 h was centrifuged at 4,000 rpm for 10 min. The cells were collected and re-suspended into a suspension of 1×10^9 colony-forming units (CFU) $l^{-1}$by adding a suitable amount of sterile water. After confirming the bacterial deletion by culturing feces on LB (Luria-Bertani) and MRS (de Man-Rogosa-Sharpe) media, antibiotic-treated mice were gavaged with 100 μl of *L. acidophilus* culture twice a week as reported (8, 9). After the bacterial colonization, mice were fed with autoclaved water and chaw. The *L. acidophilus* colonization was validated by cultivating feces each week on LB and MRS media during the treatment. Feces were also collected from mice at day 3 for 16S rRNA gene sequencing or qPCR analysis to monitor the intestinal bacteria status (1).

16S rRNA sequencing and data analysis.

Genomics DNA extraction. The microbial community DNA was extracted using MagPure Stool DNA KF kit B (Magen, China) following the manufacturer's protocols. DNA was quantified with a Qubit Fluorometer by using Qubit® dsDNA BR Assay kit (Invitrogen, USA) and the quality was checked by running aliquot on 1% agarose gel.

Library Construction. Variable regions V3–V4 of bacterial 16S rRNA gene was amplified with degenerate PCR primers, 341F (5'-ACTCCTACGGGAGGCAGCAG-3') and 806R (5'-GGACTACHVGGGTWTCTAAT-3') (10). Both forward and reverse primers were tagged

with illumina adapter, pad, and linker sequences. PCR enrichment was performed in a 50 μl reaction containing 30 ng template, fusion PCR primer and PCR master mix. PCR cycling conditions were as follows: 94 ℃ for 3 min, 30 cycles of 94 ℃ for 30 sec, 56 ℃ for 45 sec, 72 ℃ for 45 sec and final extension for 10 min at 72 ℃. The PCR products were purified with AmpureXP beads and eluted in Elution buffer. Libraries were qualified by the Agilent 2100 bioanalyzer (Agilent, USA). The validated libraries were used for sequencing on Illumina MiSeq platform (BGI, Shenzen, China) following the standard pipelines of Illumina, and generating 2 × 300 bp paired-end reads. The raw data have been deposited in the National Center for Biotechnology Information (NCBI) Gene Expression Omnibus (GEO) database under submission number PRJNA657969.

The qPCR analysis. Genomic DNA was isolated from fecal samples using the QIAamp DNA Stool Mini Kit (Qiagen) following the manufacturer's instruction. Targeted qPCR systems were applied using SybrGreen for *Lactobacillus* (forward primer: 5'-AGCAGTAGGGAATCTTCCA-3'; reverse primer (11): 5'-CACCGCTACACATGGAG-3' and *Lactobacillus acidophilus* genes (forward primer: GTGATCTTTCCTTCACTGCGT; reverse primer: TCCTCGATGGTAACTCTGTAGC). 16S rDNA forward primer: CGGTGAATACGTTCCCGG; reverse primer: TACGGCTACCTTGTTACGACTT (1) (Fig. 2F).

Test for the I3C-to-DIM-to-LTr1 conversion by *L. acidophilus*. The *L. acidophilus* cells collected by centrifugation were re-suspended in equally volumed buffers, and after titrated to preset pHs with and without protein inactivation, were individually stirred at 37 °C for different time duration with I3C or its congeners (0.15 mg/ml). Then 100 μl of such mixtures was sampled and mixed with 100 μl methanol. After centrifuging at 13000 rpm for 15 min, 100 μl of the supernatant was subjected to LC-MS analysis. The blank MRS medium and the conventional *L. acidophilus* culture were subjected to the transformation test in the same way (Figs. S7, S8, S11, S12 and S15).

The *in vivo* and *ex vivo* conversion test. After fasting for 8 h, ICR mice were co-ingested with 5-Cl-indole (in a 2% solution of DMSO in corn oil) and formaldehyde (20% in water) at doses of 150 mg/kg and 50 μl/kg, respectively. Animals were sacrificed 15 min after the

ingestion to collect urine and feces. In the *ex vivo* experiments, stomach, small intestine, and large intestine were quickly isolated from ICR mice and placed in organ chambers containing Tyrode's solution. 5-Cl-indole (100 mg/l, 10 μl) and formaldehyde (20% in water, 10 μl) were injected into the gastrointestinal tubes and incubated at 37 ± 2℃ for 24 h. Tissues were accurately weighed and grinded into homogenates with deionized water. Homogenate samples were extracted with 5-fold volumes of ethyl acetate. The mixture was vortex-mixed for 3 min and then centrifuged at 8,000 rpm for 10 min. The supernatants were evaporated to dryness which was re-suspended in 200 μl of methanol and centrifuged twice at 13,000 rpm for 10 min. A 10-μl aliquot of the supernatant from each sample was injected for the LC-MS/MS analysis performed on a reverse-phase column (Ultimate XB-C18 (100 × 2.1 mm, 3.0 μm)). A mobile phase consisting of 10 mM tetrabutylammonium fluoride in water (A) and acetonitrile (B) was delivered at a flow rate of 0.5 ml/min using the following gradient program: 10% (B) from 0→1.0 min, 10-40% (B) from 1.0→7.0 min, 40-90% (B) from 7.0→10.0 min, 90-95% (B) from 10.0→11.0 min, 95% (B) from 11.0→15.0 min, 95-10% (B) from 15.0→15.1 min, 10% (B) from 15.1→20.0 min (Fig. 6).

Chemical synthesis. All the reactions were carried out under nitrogen with anhydrous solvents in flame-dried glassware, unless otherwise noted. The chemicals used were of analytical grade. HPLC analysis was performed on a reverse-phase column (Agilent Proshell 120 EC-C18 (150 × 4.6 mm, 4 μm)) with a gradient elution system from 30% acetonitrile to 100% acetonitrile in water (supplemented with 0.1% formic acid) for 30 min with a flow rate of 0.8 ml/min. The results were analyzed by Agilent Qualitative Analysis software. DIM, I3A and I3CA were purchased from Aladdin Reagent Co. (Shanghai, China). ICZ and LTr1 were synthesized according to the outlined procedures (12, 13).

Capture of I3C-derived intermediate by thioglycol. To a solution of I3C (0.68 mmol, 100 mg) in acetonitrile (7 ml), one drop of HCl (37% in water) was added followed by being stirred for 30 min. Thioglycol (2.04 mmol, 0.15 ml) was added subsequently and the reaction mixture was stirred at room temperature for 24 h. The reaction was quenched by adding saturated $NaHCO_3$ (10 ml), and the mixture was extracted with CH_2Cl_2 (3 × 14 ml). The organic layer was washed with water and dried over Na_2SO_4. Removal of the solvent gave a residue which

was purified by column chromatography (CC) over silica gel using mixtures of petroleum ether and ethyl acetate with growing polarity (Fig. S9).

Derivation of I3C and its analogues. To a dried vial preloaded with I3C (or an I3C analogue) (1 mmol) and a reagent (I3CA, indole analogues or DIM) (1 mmol), the degassed solution (H_2O/MeOH = 1:1, v/v; 2 ml) was injected under N_2 flow. To the resulting mixture being stirred at 37 ± 2℃ for 4 h, a saturated aqueous $NaHCO_3$ (10 ml) was added followed by the extract of the reactant solution with EtOAc (3 × 10 ml). The combined organic layer was washed with brine (10 ml), dried over Na_2SO_4 and concentrated *in vacuo*. The dryness obtained was dissolved in acetonitrile for the (semi-preparative) HPLC (refinement) analysis. This protocol was subsequently applied for: (1) the decarboxylative Claisen condensation between 5-MeO-I3C and I3CA (Fig. S11); (2) the *in situ* incorporation of formaldehyde into DIM analogues via the reaction of I3C with substituent-labeled indoles (Figs. S10, S12B–12C and S13 and S35–42); (3) the coupling between 5-Cl-I3C and DIM into 5'-Cl-LTr1, 5''-Cl-LTr1 and 5,5',5''-triCl-LTr1 (Fig. 5 and S45–53) and (4) the preparation of mono-labeled DIMs via the bimolecular nucleophilic substitution reaction between indole analogues (a nucleophile) with I3C derivatives (an electrophile) (Figs. S12 and S54-61). Identification of indoles investigated herein was accomplished by comparing their ^{1}H and ^{13}C NMR spectra with those recorded (14–16) (Figs. S35–42 and S54–61).

Spectral data of 5'-Cl-LTr1. ^{1}H NMR (500 MHz, acetone-d_6) δ 10.08 (1H, br s, NH-1'), 10.02 (1H, br s, NH-1''), 9.68 (1H, br s, NH-1), 7.49 (1H, d, J = 1.3 Hz, H-4'), 7.33 (1H, d, J = 7.7 Hz, H-4), 7.29-7.24 (3H, m, H-4'', H-7' and H-7''), 7.11 (1H, d, J = 7.9 Hz, H-7), 7.03 (1H, s, H-2'), 6.95-6.92 (2H, m, H-6' and H-6''), 6.94 (1H, s, H-2''), 6.84 (1H, t, J = 7.2 Hz, H-6), 6.79-6.75 (2H, m, H-5 and H-5''), 4.21 (2H, s, H-9) and 4.20 (2H, s, H-8). ^{13}C NMR (125 MHz, acetone-d_6) δ 136.89 (C-7a''), 135.95 (C-7a), 135.41 (C-7a'), 134.95 (C-2), 128.98 (C-3a), 128.81 (C-3a'), 127.44 (C-3a''), 124.50 (C-5'), 123.66 (C-2''), 123.24 (C-2'), 121.25 (C-6'), 121.04 (C-6''), 120.24 (C-6), 118.62 (C-4'), 118.57 (C-4''), 118.25 (C-4), 118.24 (C-5''), 118.19 (C-5), 115.45 (C-3'), 112.54 (C-3''), 112.46 (C-7'), 111.19 (C-7''), 110.54 (C-7), 109.21 (C-3), 22.11 (C-9) and 19.76 (C-8). HRMS (ESI) m/z: 408.1268 $[M–H]^–$ (calcd for $C_{26}H_{19}{}^{35}ClN_3$

408.1268); The [M–H]/[M–H+2] ratio was *ca.* 3:1.

Spectral data of 5”-Cl-LTr1. ^{1}H NMR (500 MHz, acetone-d_6) δ 10.21 (1H, br s, NH-1”), 9.86 (1H, br s, NH-1’), 9.71 (1H, br s, NH-1), 7.50 (1H, d, J = 7.8 Hz, H-4’), 7.35-7.33 (2H, m, H-4 and H-4”), 7.25 (2H, dd, J = 12.4, 8.7 Hz, H-7’ and H-7”), 7.10 (1H, d, J = 8.0 Hz, H-7), 7.08 (1H, s, H-2”), 6.94 (1H, t, J = 7.5 Hz, H-6’), 6.92 (1H, dd, J = 8.7, 1.7 Hz, H-6”), 6.83 (1H, s, H-2’), 6.83 (2H, t, J = 7.3 Hz, H-5’ and H-6), 6.77 (1H, t, J = 7.5 Hz, H-5), 4.21 (2H, s, H-9) and 4.19 (2H, s, H-8). ^{13}C NMR (125 MHz, acetone-d_6) δ 136.99 (C-7a’), 135.96 (C-7a), 135.27 (C-7a”), 134.42 (C-2), 129.04 (C-3a), 128.56 (C-3a”), 127.68 (C-3a’), 125.14 (C-5”), 123.92 (C-2”), 122.59 (C-2’), 121.29 (C-6”), 121.03 (C-6’), 120.26 (C-6), 118.73 (C-4”), 118.43 (C-4’), 118.31 (C-4), 118.23 (C-5’), 118.03 (C-5), 115.35 (C-3’), 112.62 (C-3”), 112.58 (C-7”), 111.17 (C-7’), 110.51 (C-7), 109.82 (C-3), 21.95 (C-9) and 19.88 (C-8). HRMS (ESI) *m/z*: 408.1265 [M–H]$^-$ calcd for $C_{26}H_{19}{}^{35}ClN_3$ 408.1268); The [M–H]/[M–H+2] ratio was close to 3:1.

Spectral data of 5,5’,5”-triCl-LTr1. ^{1}H NMR (500 MHz, acetone-d_6) δ 10.35 (1H, br s, NH-1”), 10.21 (1H, br s, NH-1’), 10.08 (1H, br s, NH-1), 7.58 (1H, d, J = 1.4 Hz, H-4’), 7.44 (2H, m, H-4 and H-4”), 7.40 (2H, dd, J = 8.6, 2.4 Hz, H-7’ and H-7”), 7.26 (1H, s, H-2”), 7.25 (1H, d, J = 8.6, 2.4 Hz, H-7), 7.07 (1H, s, H-2’), 7.07 (2H, d, J = 8.6, 2.4 Hz, H-6’ and H-6”), 6.96 (1H, ddd, J = 8.6, 3.1, 1.7 Hz, H-6), 4.36 (2H, s, H-9) and 4.33 (2H, s, H-8). ^{13}C NMR (125 MHz, acetone-d_6) δ 136.69 (C-7a’), 135.44 (C-7a”), 135.28 (C-2), 134.38 (C-7a), 130.11 (C-3a), 128.68 (C-3a’), 128.49 (C-3a”), 125.21 (C-5”), 124.47 (C-5’), 124.05 (C-5), 123.74 (C-2”), 123.62 (C-2’), 121.40 (C-6”), 121.18 (C-6’), 120.31 (C-6), 118.13 (C-4”), 117.94 (C-4’), 117.69 (C-4), 114.89 (C-3’), 112.69 (C-3”), 112.65 (C-7”), 112.08 (C-7’), 111.92 (C-7), 109.40 (C-3), 21.94 (C-9) and 19.65 (C-8). HRMS (ESI) *m/z*: 476.0491 [M–H]$^-$ (calcd for $C_{26}H_{17}{}^{35}Cl_3N_3$, 476.0488); The abundance ratio of [M–H]/[M–H+2] /[M–H+4]/[M–H+6] was around 27:27:9:1 with that of the former three looked like 3:3:1.

Reaction between indole and formaldehyde at their physiological concentrations. Four aliquots of 100 ml solution of indole (0.25 mM) and formaldehyde (0.2 mM) in water were stirred in a sealed tube at 37 ± 2 ℃ at pH 8 for 12h, 24h, 48h and 72h, respectively. And then, each reaction mixture was subjected to LC-MS analysis for the I3C and DIM abundance.

(Fig. S12).

Crystal structure of LTr1. The prism of LTr1 crystalized from MeOH was analyzed at 296 K on an Agilent SuperNova diffractometer equipped with Cu–Kα radiation (λ = 1.54178 Å) (Fig. S16). It was found to be orthorhombic, space group *Iba2*, a = 17.3777 (13) Å, b = 27.991 (2) Å, c = 8.2909 (6) Å, V = 4032.8 (5) $Å^3$, Z = 8, D_x = 1.237 g/cm^3, μ = 0.569 mm^{-1} and F (000) = 1584.0; crystal dimensions: 0.26 × 0.22 × 0.18 mm^3; 3139 unique reflections with 2497 obeying the $I \geq 2\sigma (I)$; $R1$ = 0.0403, $wR2$ = 0.1157, S = 1.042; supplementary publication no. CCDC-1499138. The structure was solved by the direct method (SHELXS-97) and refined using full-matrix least-squares difference Fourier techniques. Crystallographic data in CIF format have been deposited in the Cambridge Crystallographic Data Centre [available free of charge at http://www.ccdc.cam.ac.uk/deposit or from the CCDC, 12 Union Road, Cambridge CB21EZ, UK; fax: (+44) 1223-336-033; or e-mail: deposit@ccdc.cam.ac.uk].

Statistical analysis. All statistical analysis was performed using Prism 6.0 software (GraphPad, San Diego, CA, USA). P < 0.05 was considered statistically significant. For comparisons between two groups unpaired, P values was calculated by two-tailed Student's *t* test. For the comparisons of three or more groups, one-way ANOVA using multiple comparison method was performed. Two-way ANOVA was used for analysis that involved two variables, followed by multiple comparisons, comparing the mean of each column with the mean of every other column. *P < 0.05; ** P < 0.01; ***P < 0.001; ****P < 0.0001. Error bars, mean ± SD or SEM, as indicated. No statistical methods were used to predetermine sample size. The investigator was blinded to the group allocation during the experiment and/or when assessing the outcome.

Data availability. The data that support the findings of this study are available from the corresponding author upon request.

Table S1. A list of bioactivities described for I3C and its rodent-produced catabolites.

Compounds*	Bioactivity selections
I3C	Antibacterial (17); anticancer (18, 19); hepatoprotection (18); gut microbiome modulation (20); cardioprotection (21); multiple-drug-resistance reversal (22);

	antioxidant (23); repressing aryl hydrocarbon receptor (AhR) expression (24).
I3A	Antioxidant (25); antibacterial (26); phytotoxic (27); AhR agonist (28).
I3CA	Antioxidant (29); antibacterial (30).
ICZ	Cytostatic (31); antiestrogenic (31); AhR agonist (32); tumor promoter (33).
DIM	Immunomodulation (34); anticancer (35); insulin sensitivity-improvement (36); intestinal permeability-regulation (37); tumor-invasion inhibition (38); selective estrogenic activity (39); stimulating TGF-α expression (40); inducing the death receptor DR5 expression (41).
LTr1	Antiestrogenic (33); weak AhR agonist (42).

*I3C, indole-3-carbinol; I3A, indole-3-carbaldehyde; I3CA, indole-3-carboxylic acid; ICZ, indolo[3,2-*b*]carbazole; DIM, 3,3'-diindolylmethane; LTr1, 2-(indol-3-ylmethyl)-3,3'-diindolylmethane.

Table S2. Cytotoxicity comparison of I3C and its catabolites (IC_{50} in μM).

Compds. / Cell lines	I3A	I3CA	ICZ	I3C	DIM	LTr1	doxorubicin
A549	>10	>10	4.21±0.21	>10	>10	0.73±0.24	1.76±0.13
A375	>10	>10	7.23±0.31	>10	>10	1.40±0.11	1.95±0.09
SW480	>10	>10	5.45±0.14	>10	5.76±0.17	9.43±0.34	1.17±0.11
HepG-2	>10	>10	>10	>10	9.76±0.34	2.18±0.12	2.60±0.18
MCF-7	>10	>10	2.14±0.24	>10	2.54±0.14	1.87±0.15	1.71±0.15
CaoV-3	>10	>10	>10	>10	7.07±0.45	4.47±0.12	2.04±0.37

Table S3. Human fecal bacteria screened for the I3C conversion.

No.	Strains	Media[a]	Culture[b]
1	*Lactobacillus johnsonii*	MRS	aerobic
2	*Lactobacillus acidophilus*	MRS	aerobic
3	*Lactobacillus gasseri*	MRS	aerobic
4	*Escherichia coli*	LB	aerobic
5	*Enterococcus avium*	LB	aerobic
6	*Enterococcus faecalis*	LB	aerobic
7	*Enterococcus casseliflavus*	LB	aerobic
8	*Bifidobacterium breve*	BS	anaerobic
9	*Bifidobacterium adolescentis*	BS	anaerobic
10	*Bifidobacterium longum* subsp. *longum*	BS	anaerobic
11	*Bifidobacterium bifidum*	BS	anaerobic
12	*Bifidobacterium catenulatum*	BS	anaerobic
13	*Bifidobacterium angulatum*	BS	anaerobic
14	*Clostridium clostridioforme*	GAM	anaerobic
15	*Clostridium butyricum*	GAM	anaerobic
16	*Clostridium perfringen*	GAM	anaerobic
17	*Bacteroides vulgatus*	GAM	anaerobic
18	*Bacteroides fragilis*	GAM	anaerobic
19	*Bacteroides thetaiotaomicron*	GAM	anaerobic
20	*Streptococcus salivarius*	GAM	anaerobic

[a] GAM, Gifu anaerobic medium; BS, Bifidobacterium medium; LB, Luria-Bertani medium; MRS, de Man-Rogosa-Sharpe agar. [b] At 37 ºC for 24 h.

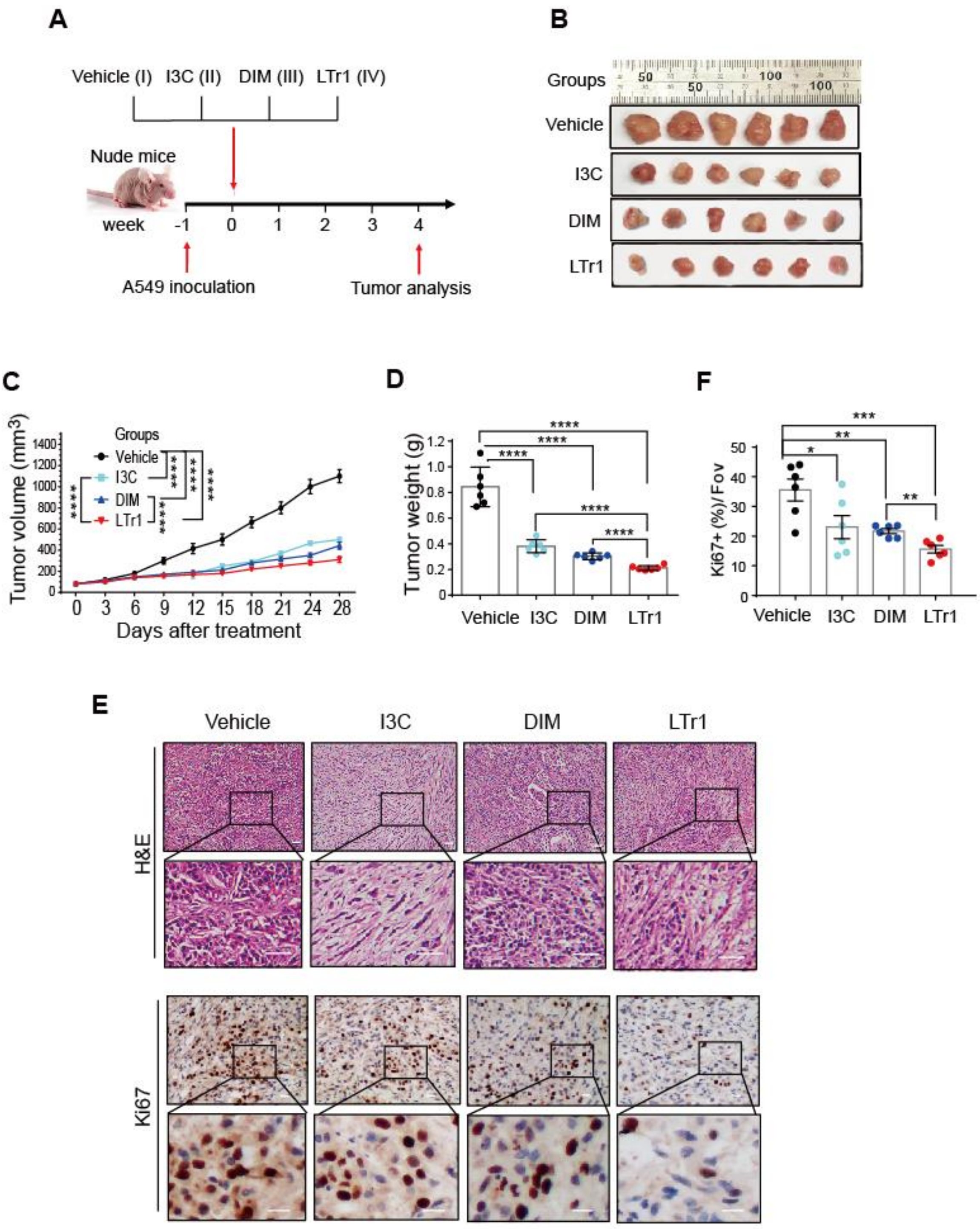

Figure S1. LTr1 was more effective than DIM and I3C in the A549-xenografted nude mice. (A) Experimental design using four-week-old nude mice randomized to groups I–IV (n = 6), which were administered orally with vehicle (100 μl, a 2% solution of DMSO in corn oil), I3C, DIM and LTr1 daily, respectively. (B–F) The antitumor activity was evidenced from the tumor

volume (B and C), weight (D, each dot representing a mouse), the representative H&E staining and Ki67 immunostaining (E) as well as its expression level (F) in cancer sections after four-week treatment (E, scale bar, 50 μm for H&E staining; 20 μm for Ki67 immunostaining; boxed regions in the top row images were enlarged in the bottom row of panels; F, Each individual data point represented an average of five FoVs of a single tumor from a mouse). *$p < 0.05$, **$p < 0.01$, ***$p < 0.001$, ****$p < 0.0001$ by two-way ANOVA test (C) and Student's t test (D, F), comparing the means ± SEM of six biological replicates versus indicated controls.

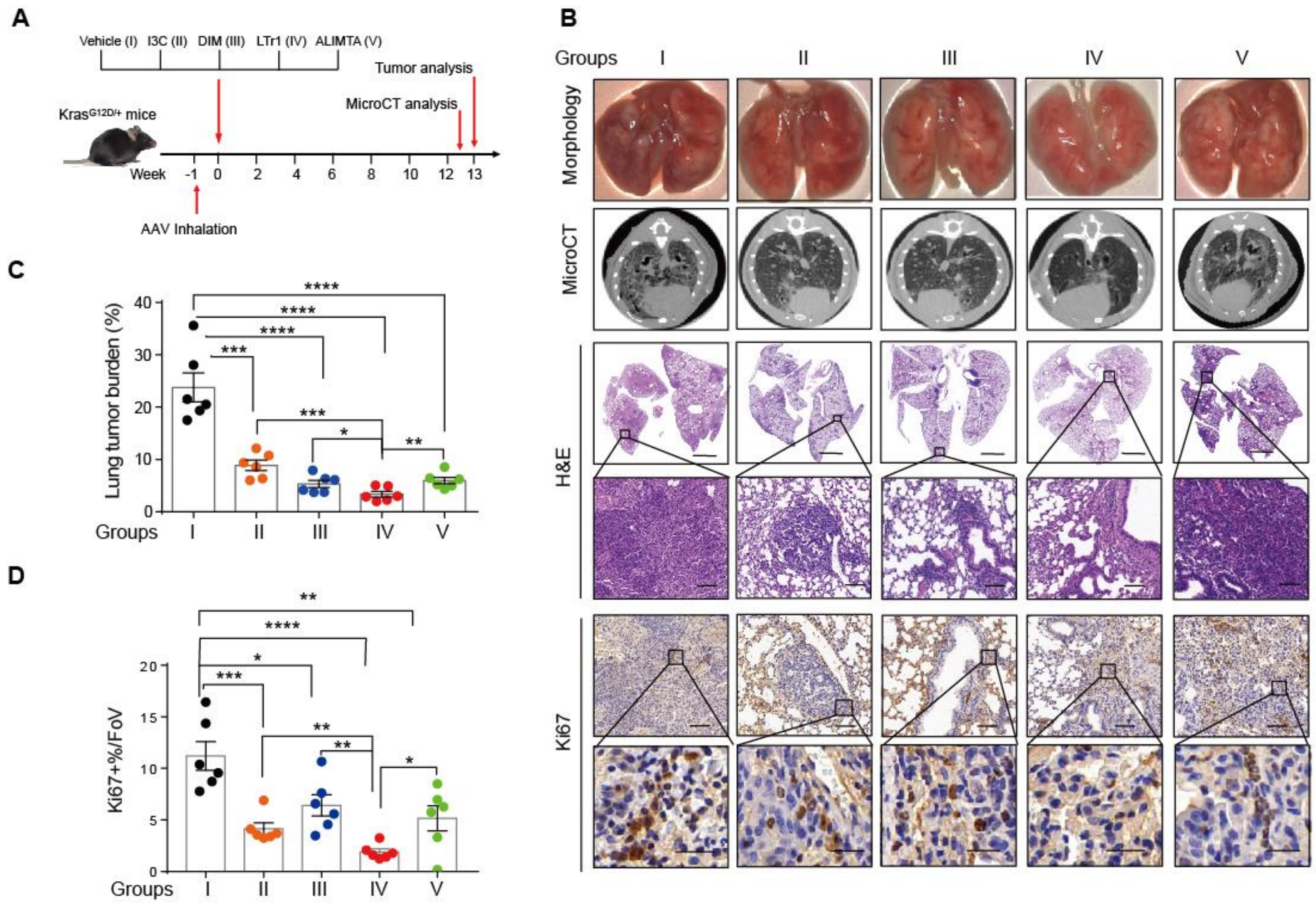

Figure S2. Comparison of the anticancer efficacy of LTr1 in *Kras*$^{G12D/+}$ mice with those of DIM, I3C and pemetrexed disodium.

(A) Experimental design. Five-week-old *Kras*$^{G12D/+}$ mice were randomized to five treatment groups (I–V, n = 6) and treated for 13 weeks. Groups I-IV were administered via oral gavage. Group I: vehicle (100 µl, a 2% solution of DMSO in corn oil). Groups II–IV: 150 mg/kg/d of I3C, DIM, and LTr1, respectively. Group V: 150 mg/kg/d of pemetrexed disodium (ALIMTA) administered via intraperitoneal injection (twice a week). (B) Anticancer efficacies of LTr1, DIM, I3C and pemetrexed disodium were compared in terms of representative lung morphology, microCT image, H&E staining of lung lobes [scale bar, 2,000 µm; boxed regions in the top row images were enlarged in the bottom row of related panels (scale bar, 100 µm)], and Ki67 immunostaining of lung sections (scale bar, 100 µm; higher magnifications of boxed regions illustrated in the bottom row of panels with a scale bar of 20 µm). (C and D) Quantifications of tumor burden (C) and Ki67 immunostainings (D) in mouse lungs. FoV = field of view. One point represents an average of five FoVs per lung from a mouse. *$p < 0.05$, **$p < 0.01$, ***$p < 0.001$, ****$p < 0.0001$ by Student's t test, comparing the means ± SEM of six biological replicates versus indicated controls.

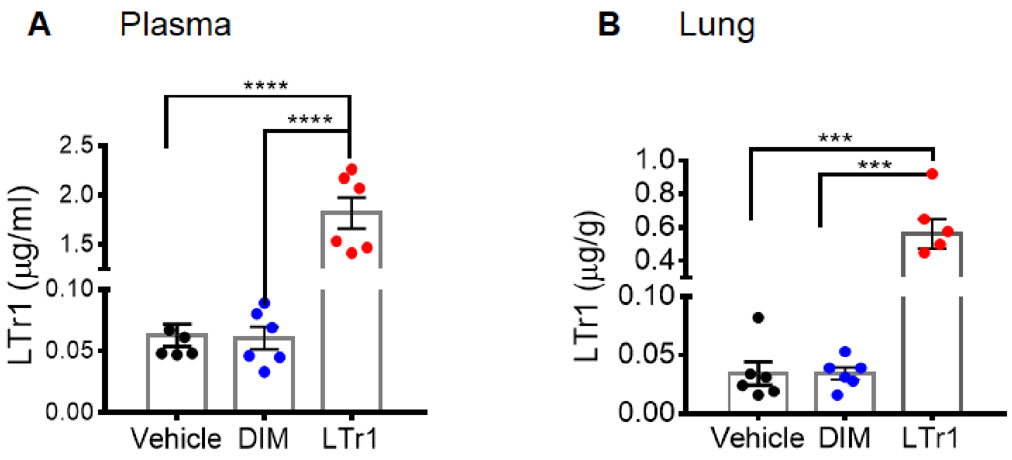

Figure S3. Penetration of LTr1 in *Kras*$^{G12D/+}$-driven lung cancer tissues. The LTr1 concentration in plasma (A) and lung (B) of *Kras*$^{G12D/+}$ mice was quantified by LC-MS analysis after a 13-week treatment with vehicle, DIM or LTr1 (n = 6, see **Fig. S2**). ***$p < 0.001$, ****$p < 0.0001$ by Student's t test, comparing means ± SEM of six biological replicates versus indicated controls.

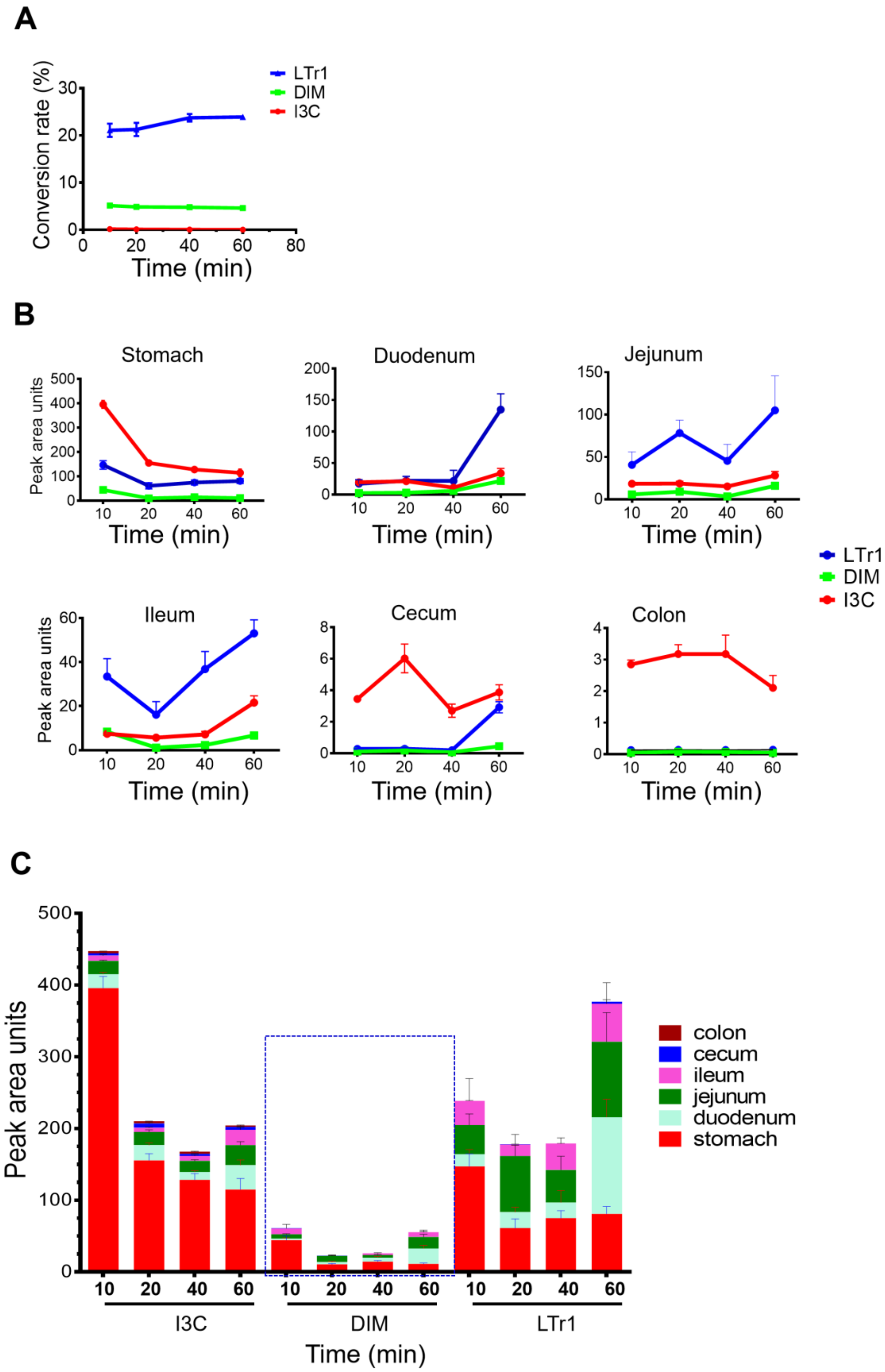

Figure S4. Time-course comparison for LTr1, DIM and I3C *in vitro* and *in vivo*. (A) In the artificial gastric juice, I3C was converted almost completely into DIM-LTr1 mixture (~1:5). (B) *In*

vivo conversion of I3C into DIM and LTr1 in stomach, duodenum, jejunum, ileum, cecum and colon. (C) The distribution profiles of I3C, DIM and LTr1 in the examined compartments. Data were means ± SEM of five biological replicates. Peak area ratios were proportions of I3C, DIM, and LTr1 peak areas to that of internal standard (ketoconazole 50 ng l^{-1}).

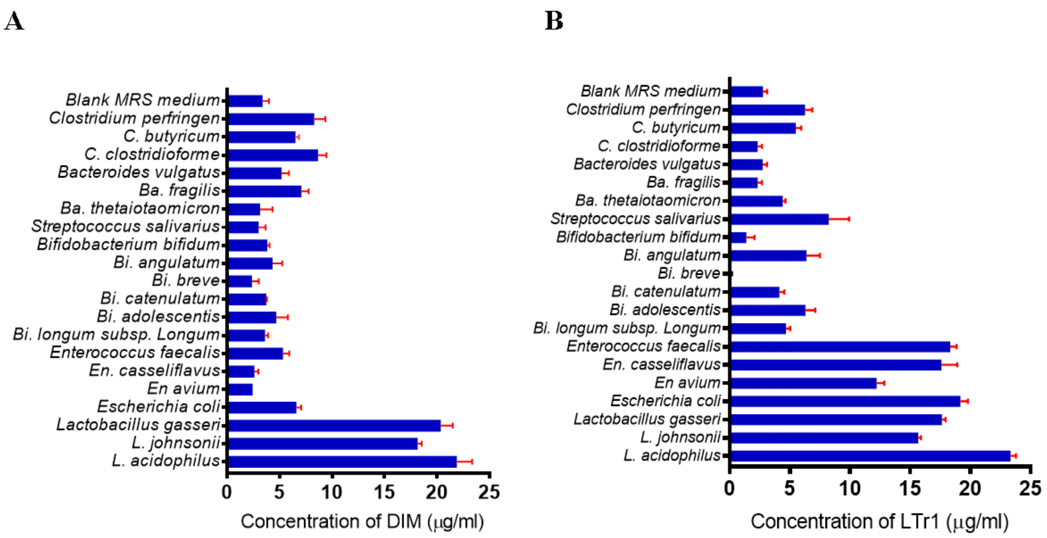

Figure S5. *L. acidophilus* promoted the *in vitro* conversion of I3C into DIM and LTr1. *In vitro* conversion of I3C into LTr1 by different bacteria isolated from human feces (OD_{600} = 2.50, see also Table S3). DIM (A) and LTr1 (B) were determined by HPLC with subtraction of spontaneous production of LTr1 in blank media (if any). Results were the average of three independent experiments. Data were means ± SEM of three biological replicates.

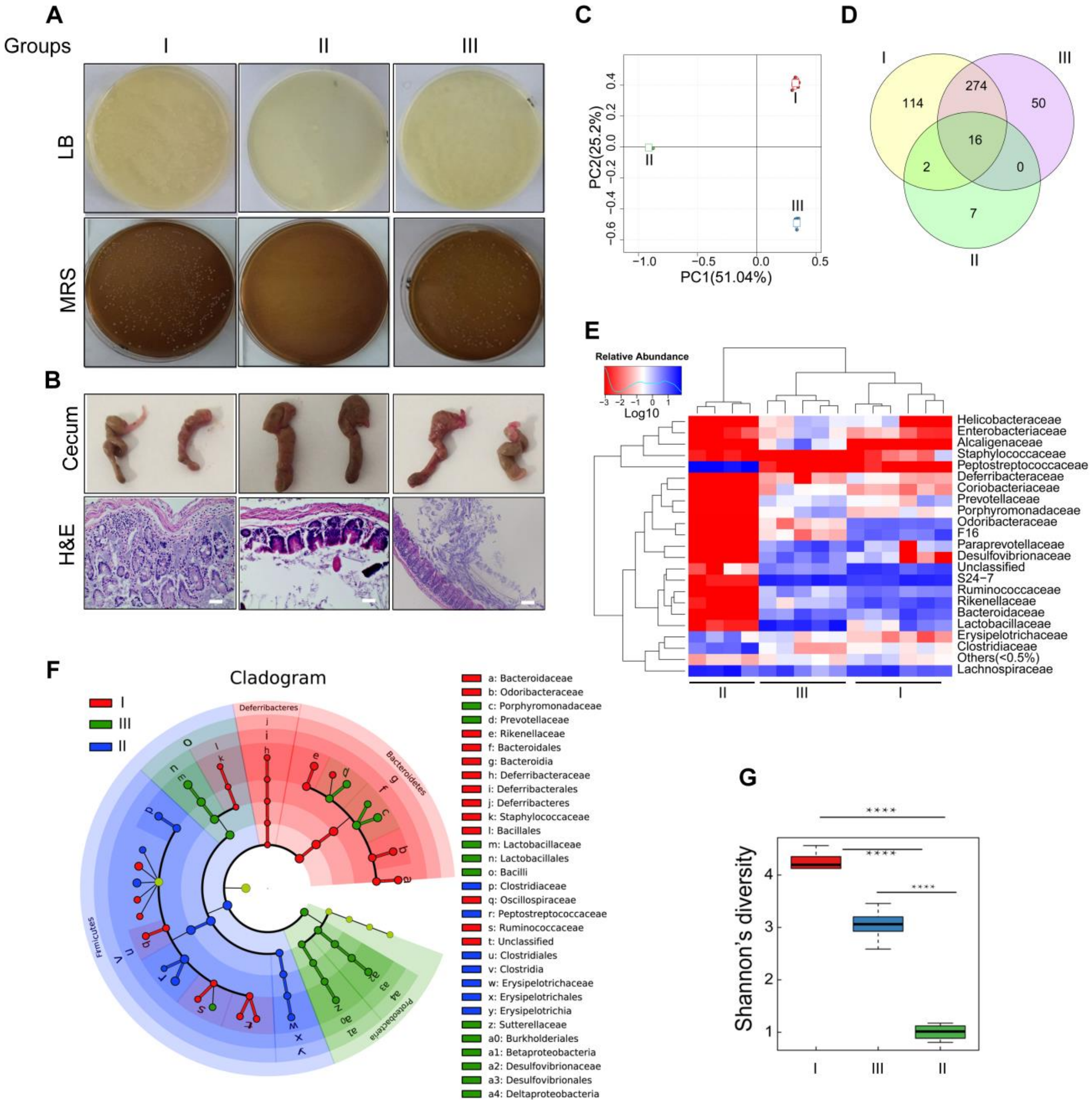

Figure S6. Validation of ACS-decontamination and *L. acidophilus* monocolonization. Nude mice were randomized to the conventional (I), ACS-decontaminated (II), and ACS-pretreated and *L. acidophilus* monocolonized groups (III). Mouse feces were collected one week after the ACS treatment or three days after the *L. acidophilus* monocolonization. (A and B) Fecal bacteria were grown on Petri dish in LB and MRS media (A), and the representative cecum and the H&E staining of cecal sections (B; scale bar, 40 μm). (C–E and G) The robustness of the ACS decontamination and *L. acidophilus* monocolonization was evidenced from the principal coordinates analysis (PCoA) (C), Venn diagram (D), the heat map (E), and the alpha-diversity measured by Shannon index (G) of mouse fecal samples based on operational taxonomic units (OTUs). (F) LEfSe (Linear discriminant analysis Effect Size) pinpoints

Lactobacillaceae as a biomarker in group III. Data were means ± SEM of 4~6 biological replicates. ****p < 0.0001 by a one-way ANOVA (multiple comparisons) versus indicated controls.

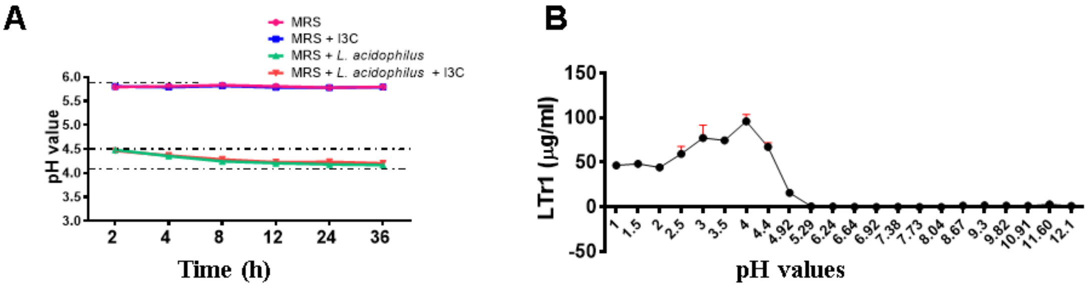

Figure S7. The H^+-promoted I3C-to-DIM-to-LTr1 transformation. (A) The *L. acidophilus* cultures with and without the I3C exposure shared a pH range of 4.0–4.5. Samples were taken for the pH comparison from two equally prepared *L. acidophilus* cultures, to one of which I3C was added at 0.15 mg/ml. The blank MRS medium was worked up in the same way. Incubations were accomplished at 37 ºC for 2, 4, 8, 12, 24 and 36 h, respectively. Data were means ± SEM of three biological replicates. (B) The optimal pH range for the I3C conversion into LTr1 *in vitro*. I3C solutions at 0.15 mg/ml in the phosphate buffers (pH 1–12) was agitated at 37 ºC at 200 rpm for 24 h. The resulting reactants therefrom were subjected to the HPLC quantification of LTr1. Data were means ± SEM of three biological replicates.

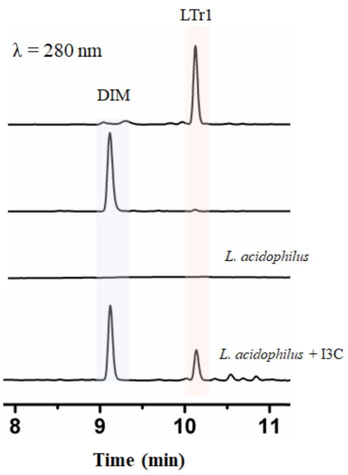

Figure S8. DIM and LTr1 were undetectable in *L. acidophilus* cells without the I3C-exposure. After cultured at 37 °C for 24 h with or without exposure to I3C (0.15 mg/ml), the *L. acidophilus* culture cells were collected by centrifugation. After crushing, the cell lysates were extracted with methanol, centrifuged and subjected to LC-MS analysis.

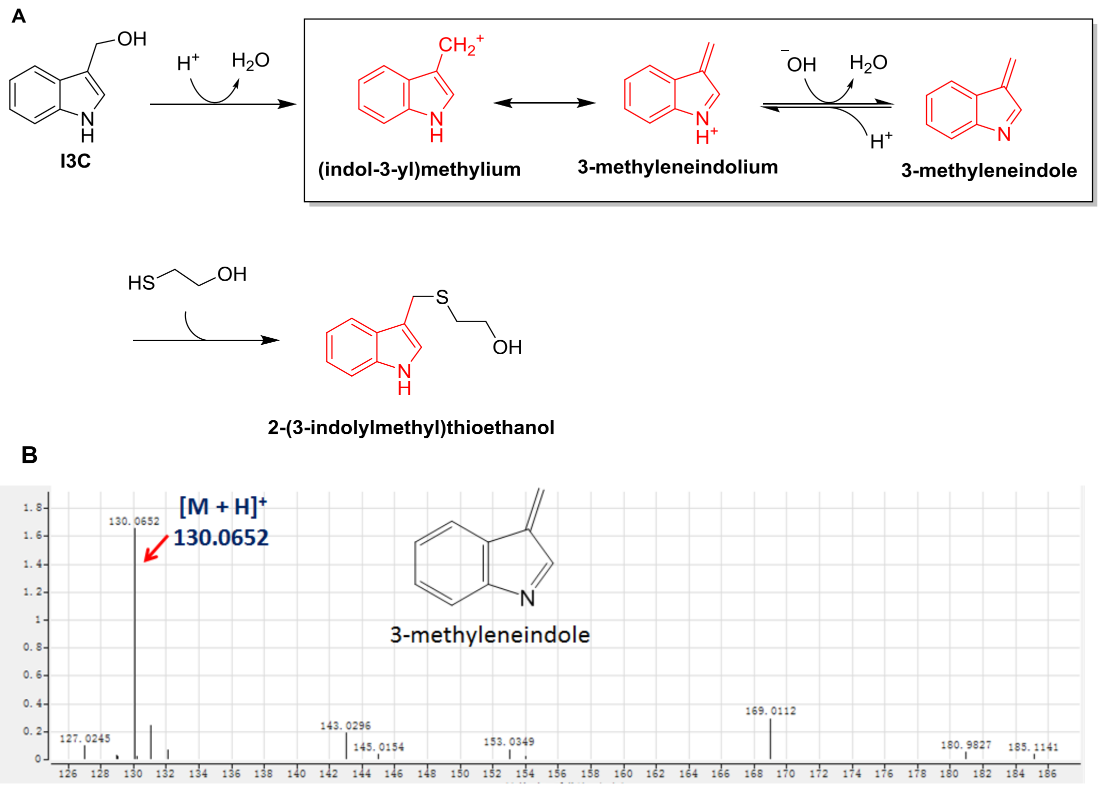

Figure S9. The I3C propensity to dehydrate. (A) The proposed intermediate formation from the I3C dehydration. (B) The ESI-MS evidence for 3-methyleneindole derived from I3C.

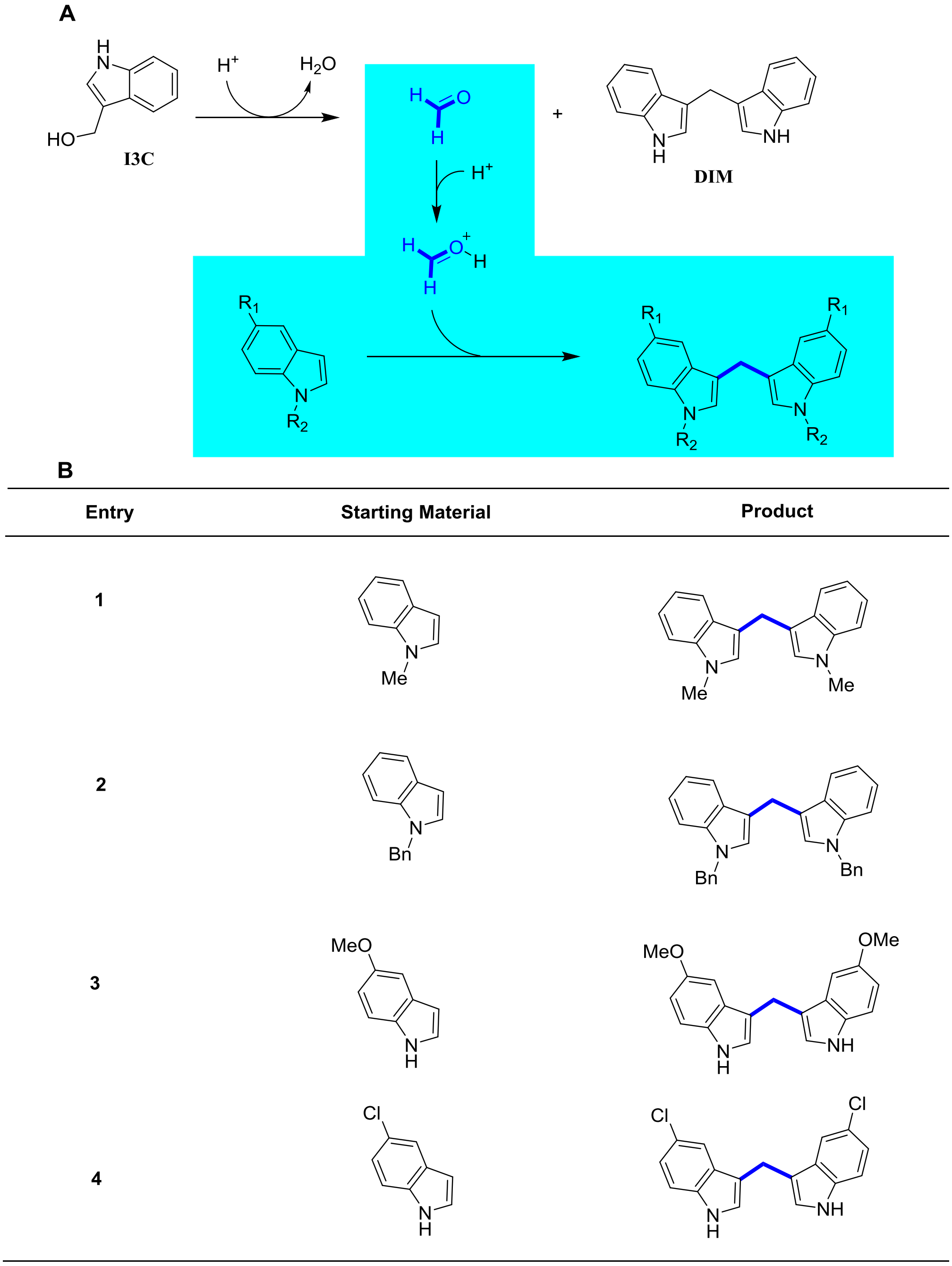

Figure S10. The capture of formaldehyde by substituent-labeled indoles. (A) The putative mechanism underlying the hybridization of formaldehyde with substituent-labeled indoles to form the corresponding DIM analogues with the label groups. (B) The list of the starting material and corresponding products with the experimental procedures (*see* "Derivation of I3C and its analogues" in Supplementary Information).

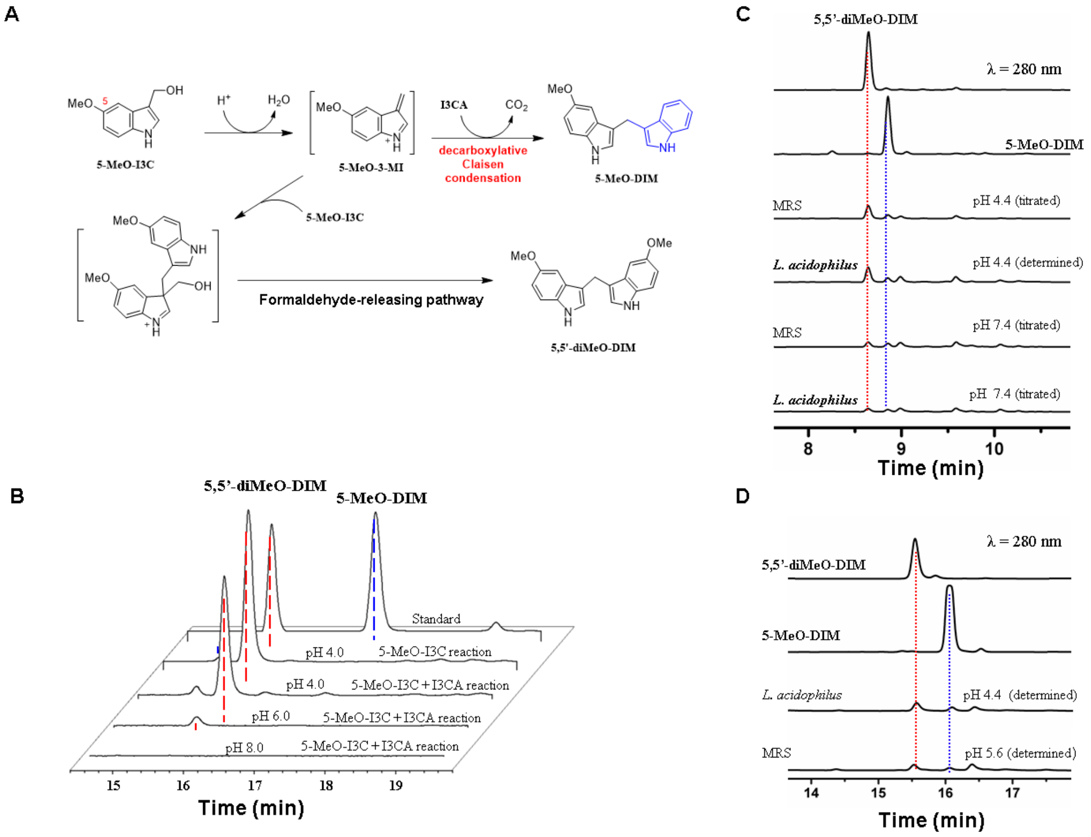

Figure S11. The decarboxylative Claisen condensation of I3CA and 3-methyleneindolium (3MI) to produce the DIM. (A) A trivial amount of 5-MeO-DIM was produced via the decarboxylative Claisen condensation of I3CA with 5-MeO-3-MI (formed *in situ* from 5-MeO-I3C), but the major product from such a reaction test was 5,5'-diMeO-DIM formed via the formaldehyde-releasing pathway. (B) HPLC profiles for products generated upon stirring two aliquots of 5-MeO-I3C solution in aqueous methanol (pH = 4, 6 or 8), with and without I3CA, respectively. (C) HPLC comparisons for the products resulting from 5-MeO-I3C and I3CA in the *L. acidophilus* culture and the blank MRS medium. The culture and medium were aliquoted into two parts which were titrated to pHs 4.4 and 7.4 using HCl and NaOH, respectively, followed by agitation with the two substrates. (D) HPLC comparisons for the products formed after the direct supplementation of 5-MeO-I3C and I3CA in the *L. acidophilus* culture and MRS medium. 5-MeO-I3C and I3C in (C) and (D) were at 0.15 mg/ml.

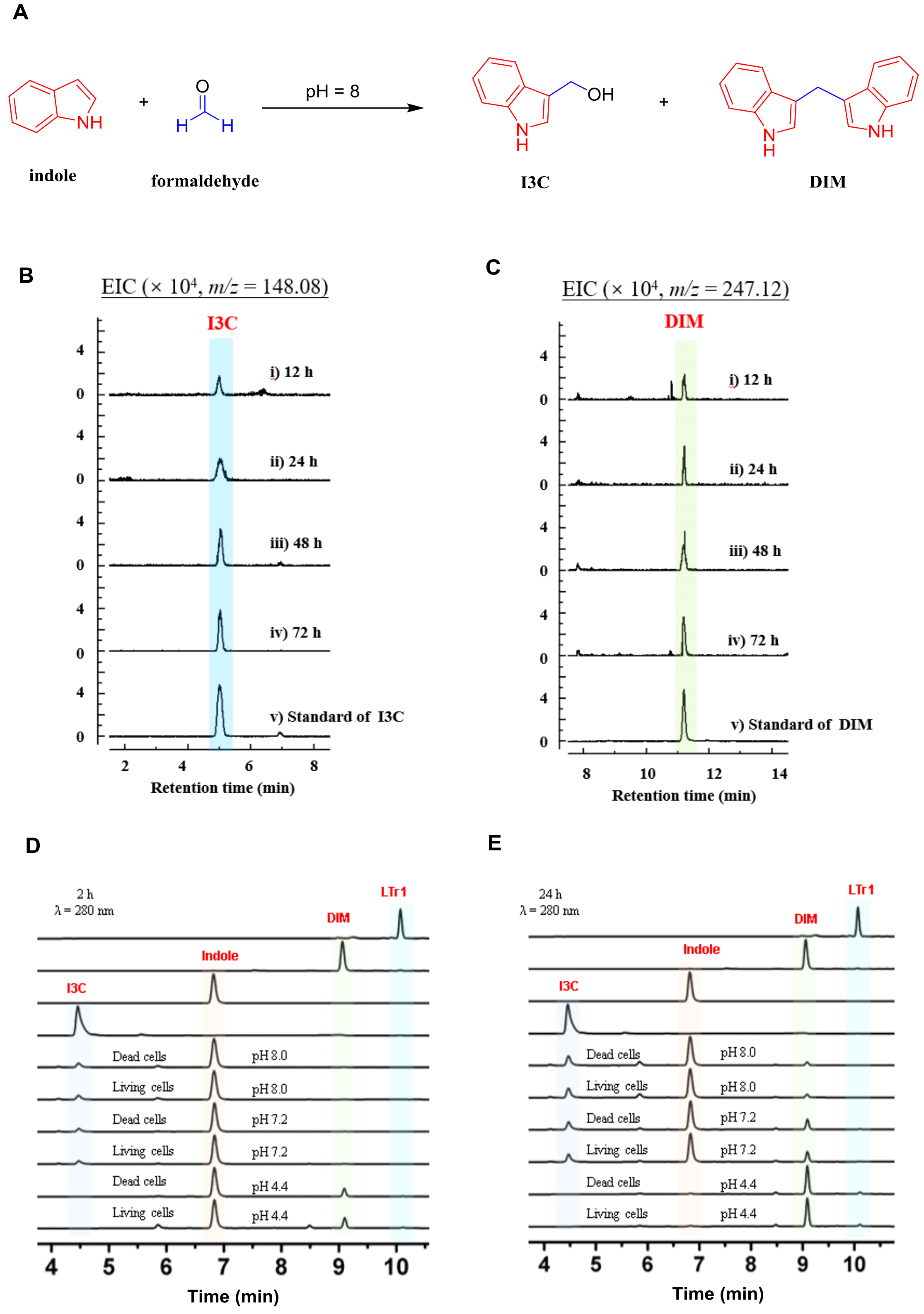

Figure S12. DIM (but not LTr1) resulting from formaldehyde-indole coupling in their physiological concentration ranges. (A) Scheme for I3C and DIM formations upon stirring the aqueous solution of indole (0.25 mM) and formaldehyde (0.2 mM) at 37 ± 2 ℃. (B–C)

LC-MS detection of I3C and DIM in the mixture with different reacting time. (D–E) HPLC detection of I3C, DIM and LTr1 formed in the buffer (pHs 4.4, 7.2 and 8.0) suspensions of living and autoclaved (or dead) *L. acidophilus* cells (120 ℃, 30 min) after the 2- and 24-hour agitations at 37 ℃ with indole (0.25 mM) and formaldehyde (0.2 mM).

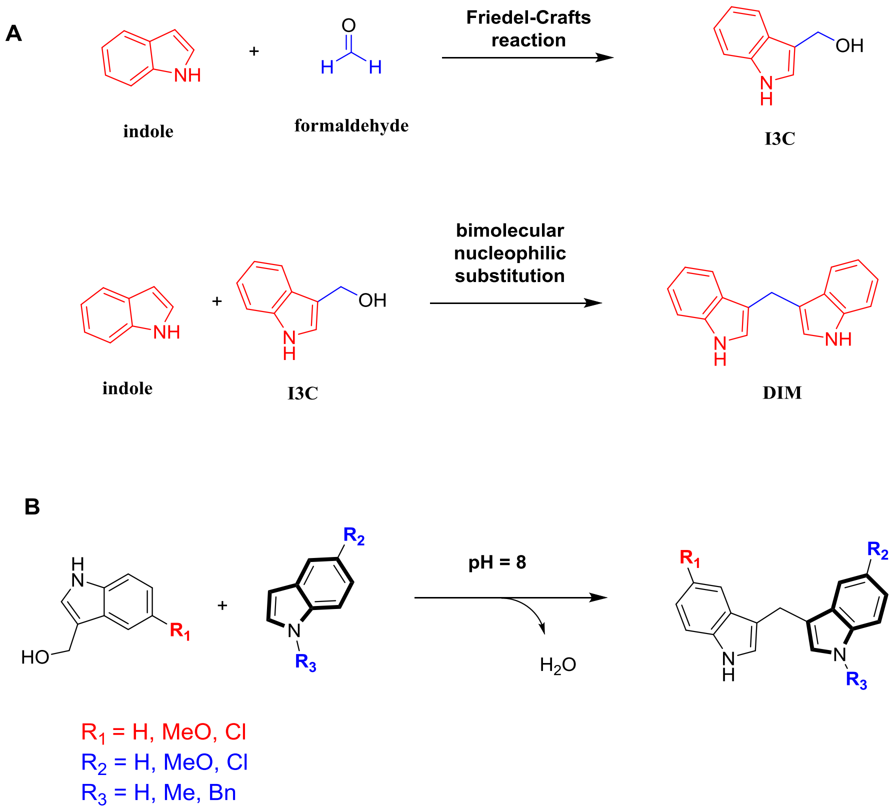

Figure S13. The I3C and DIM productivity from the coupling of indole to formaldehyde. (A) The reactivity of indole with formaldehyde and I3C. (B) The hybridization of substituted indoles with I3C derivatives.

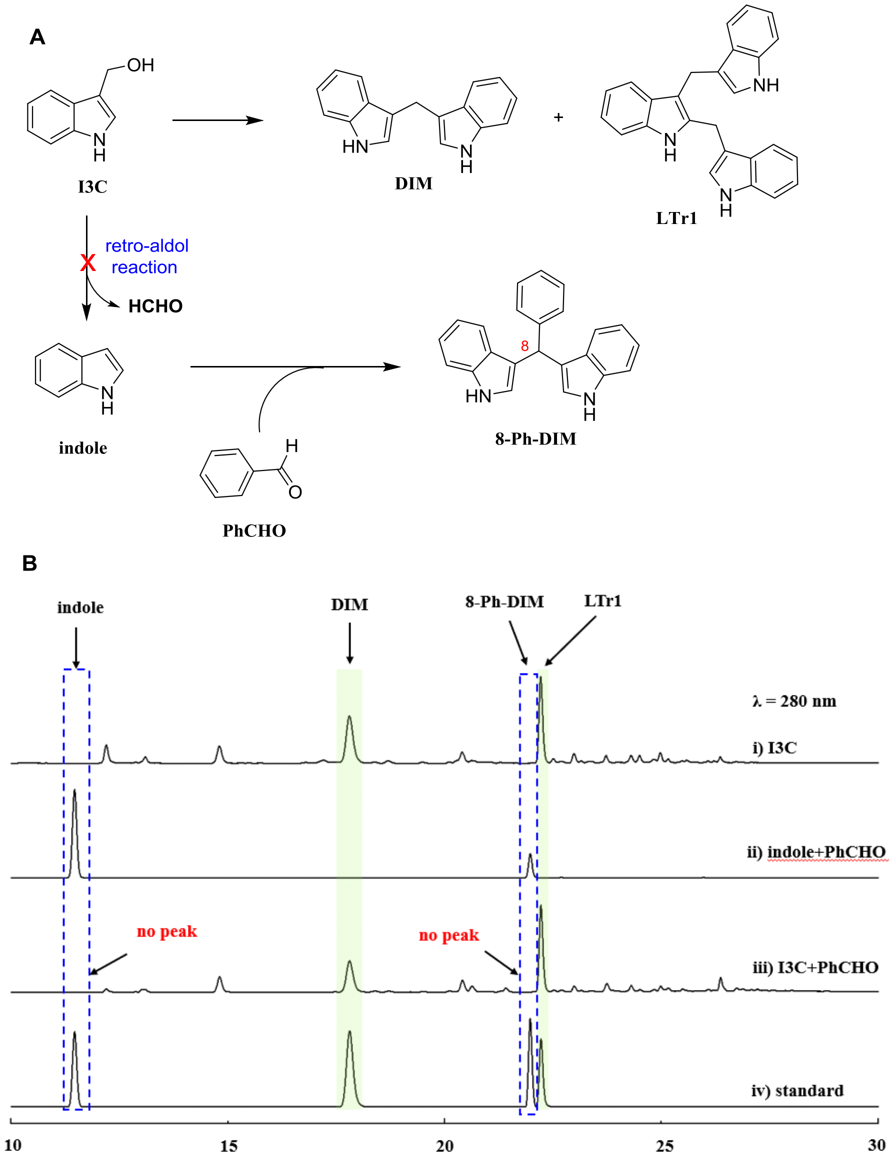

Figure S14. No indole forming from I3C via retro-aldol reaction. (A) The rationale for trapping the "I3C-derived" indole by benzaldehyde (PhCHO) to produce 8-Ph-DIM. (B) Neither indole nor 8-Ph-DIM could be HPLC-detected from the mixture after stirring I3C and PhCHO in acidic condition.

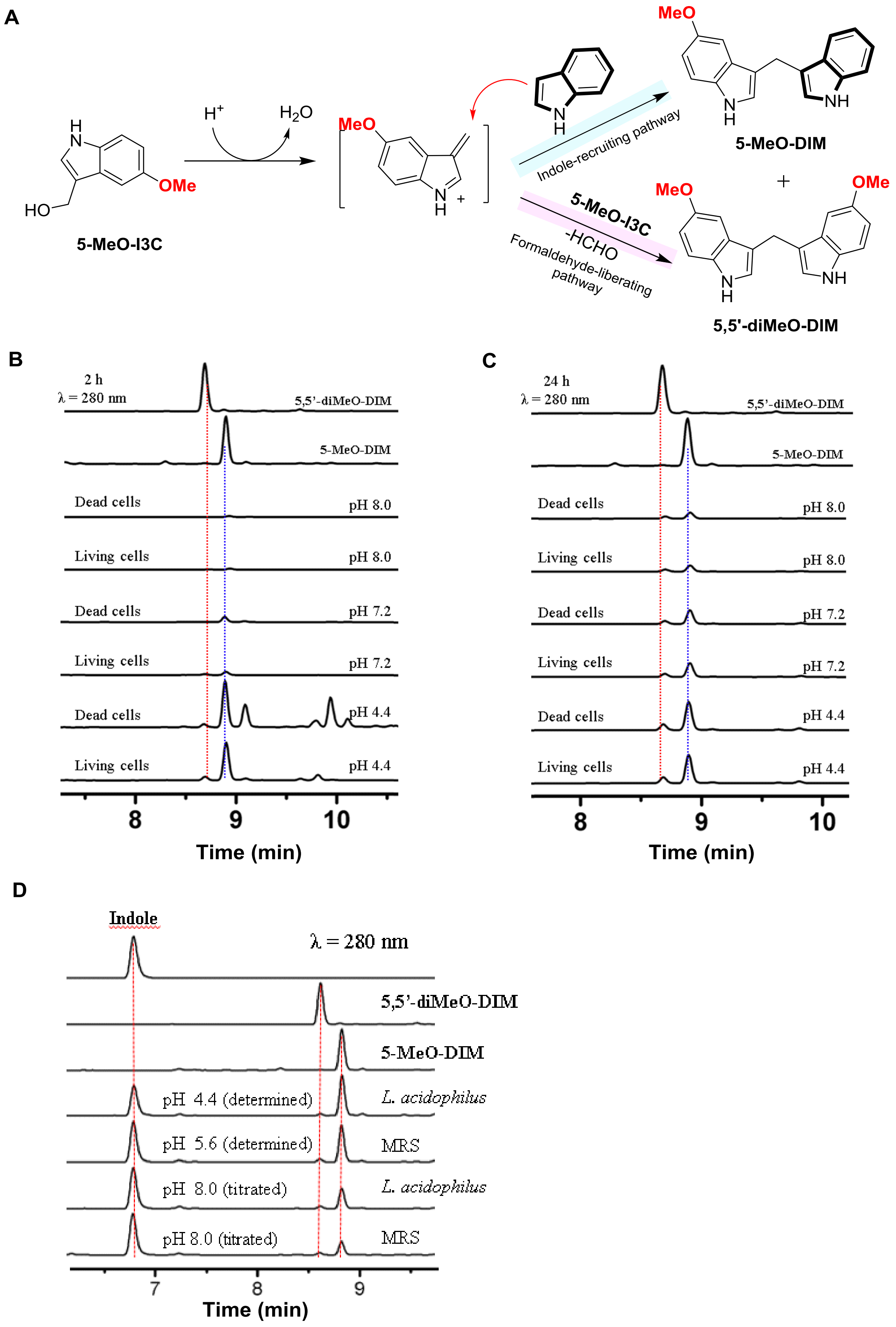

A
5-MeO-I3C
5-MeO-DIM
5,5'-diMeO-DIM
Indole-recruiting pathway
5-MeO-I3C
-HCHO
Formaldehyde-liberating pathway
B
2 h
λ = 280 nm
C
24 h
λ = 280 nm
Dead cells
Living cells
pH 8.0
pH 7.2
pH 4.4
Time (min)
D
Indole
λ = 280 nm
pH 4.4 (determined)
pH 5.6 (determined)
pH 8.0 (titrated)
L. acidophilus
MRS
Time (min)

Figure S15. Generation of 5-MeO-DIM and 5,5'-diMeO-DIM from the coupling of indole to 5-MeO-I3C. (A) Scheme for 5-MeO-DIM and 5,5'-diMeO-DIM from the two substrates. (B–C) HPLC detection of 5-MeO-DIM and 5,5'-diMeO-DIM generated in the buffer (pHs 4.4, 7.2 and 8.0) suspensions of *L. acidophilus* cells after being stirred at 37 ℃ for 2 (B) and 24 h (C) with indole and 5-MeO-I3C. (D) HPLC profiles of 5-MeO-DIM and 5,5'-diMeO-DIM generated after agitated at 37 ℃ for 24 h with indole and 5-MeO-I3C in the conventional bacterial culture and the blank MRS medium as well as their equivalents with pH titrated to 8.0.

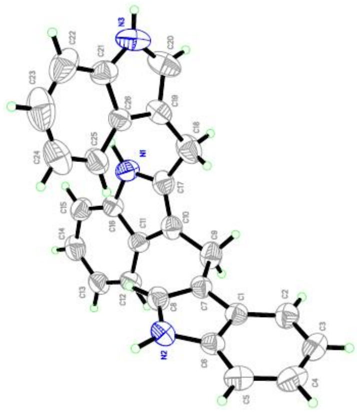

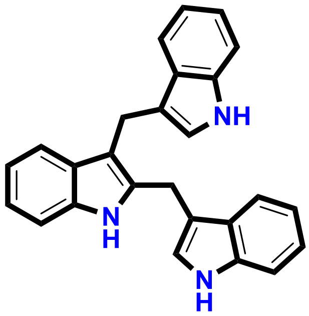

Figure S16. Crystal (left) and molecular structures (right) of LTr1.

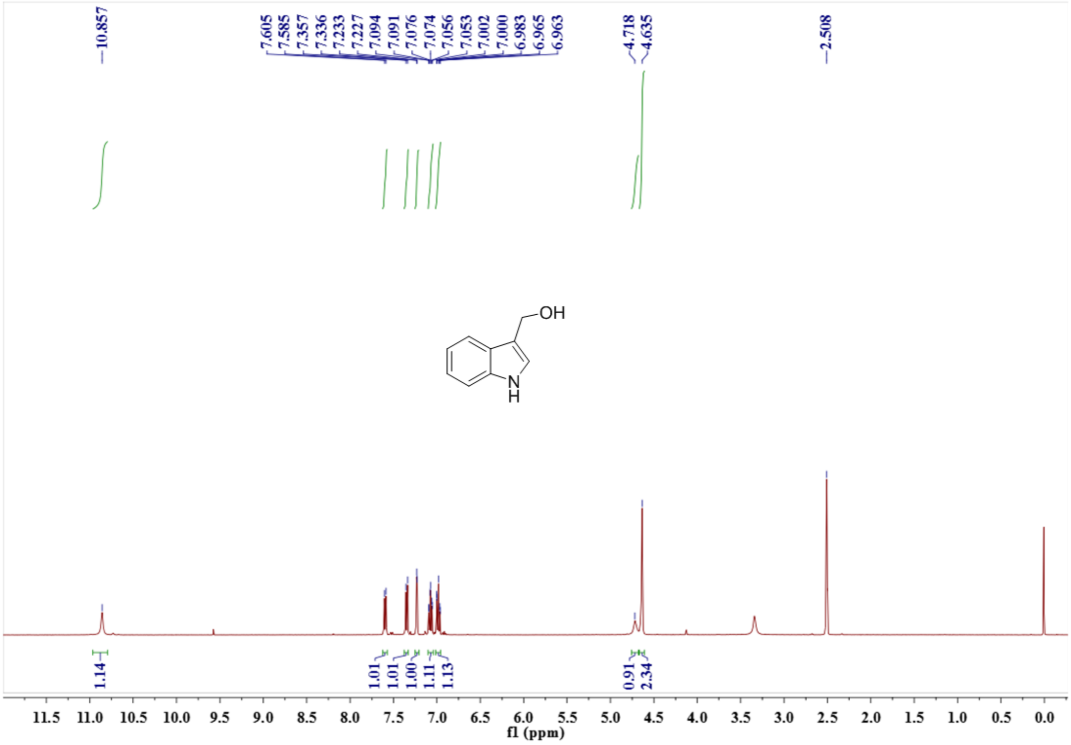

Figure S17. ^{1}H NMR spectrum of I3C (400 MHz, DMSO-d_6).

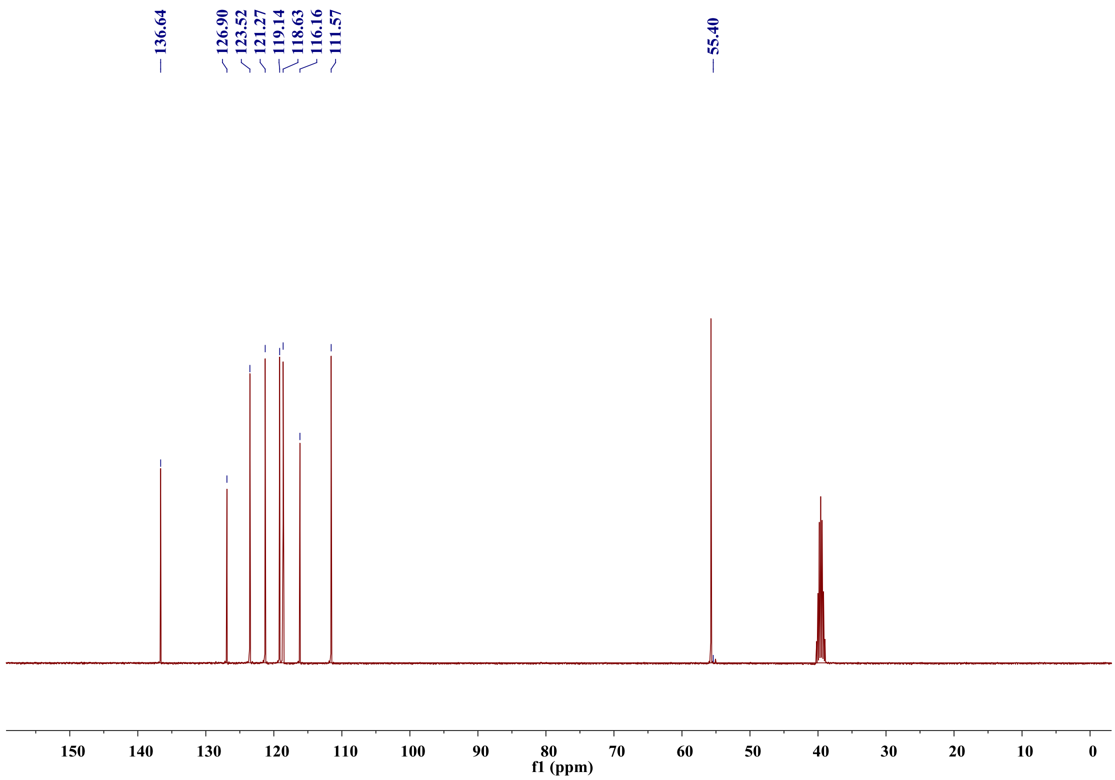

Figure S18. ^{13}C NMR spectrum of I3C (100 MHz, DMSO-d_6).

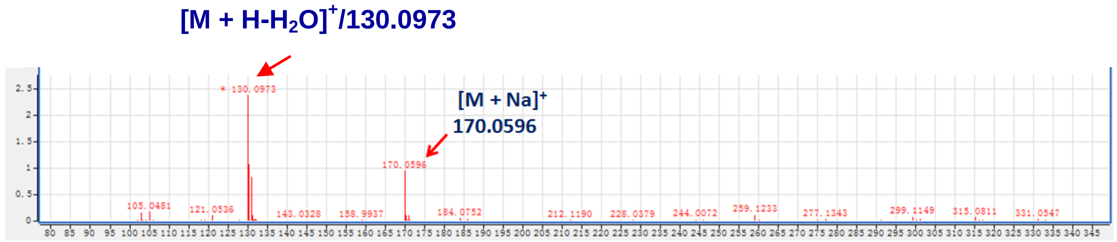

Figure S19. Pseudomolecular ions of I3C at its ESI-MS spectrum.

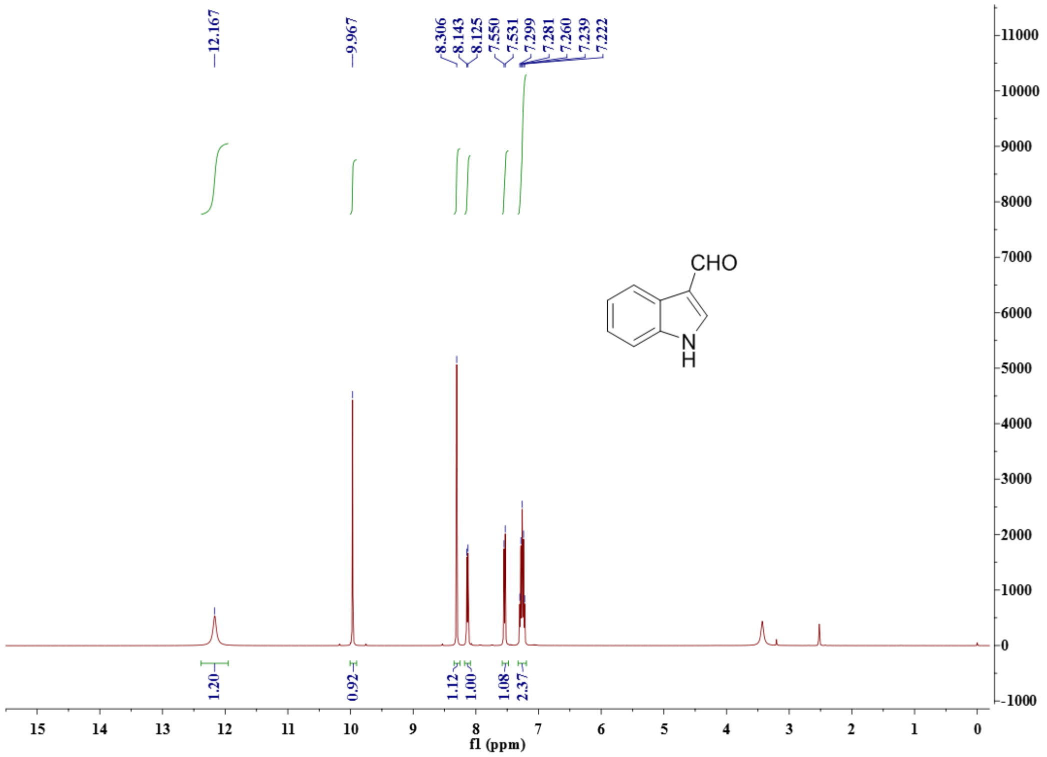

Figure S20. ^{1}H NMR spectrum of I3A (400 MHz, DMSO-d_6).

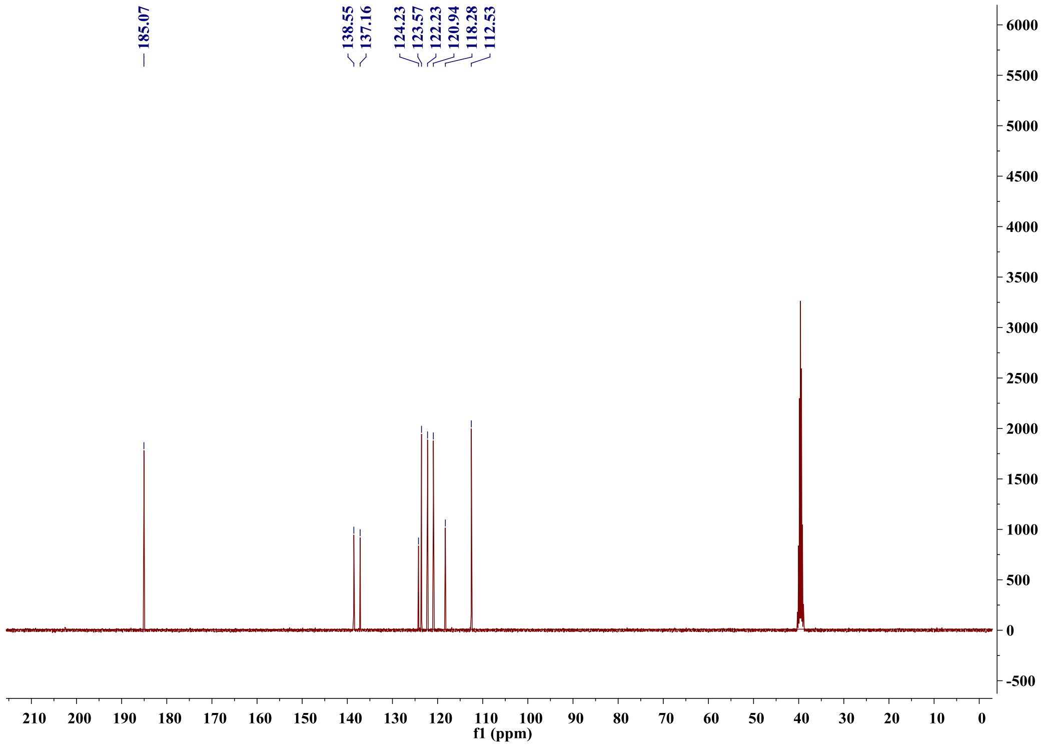

Figure S21. ^{13}C NMR spectrum of I3A (400 MHz, DMSO-d_6).

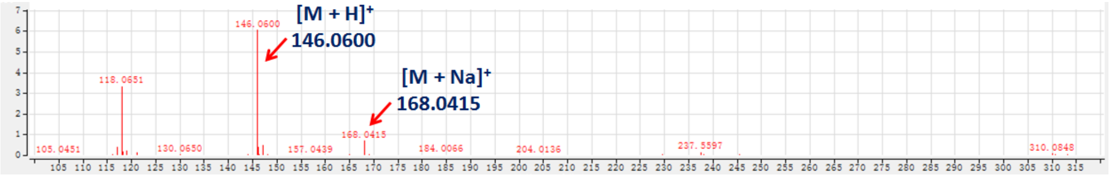

Figure S22. ESI-MS spectrum of I3A.

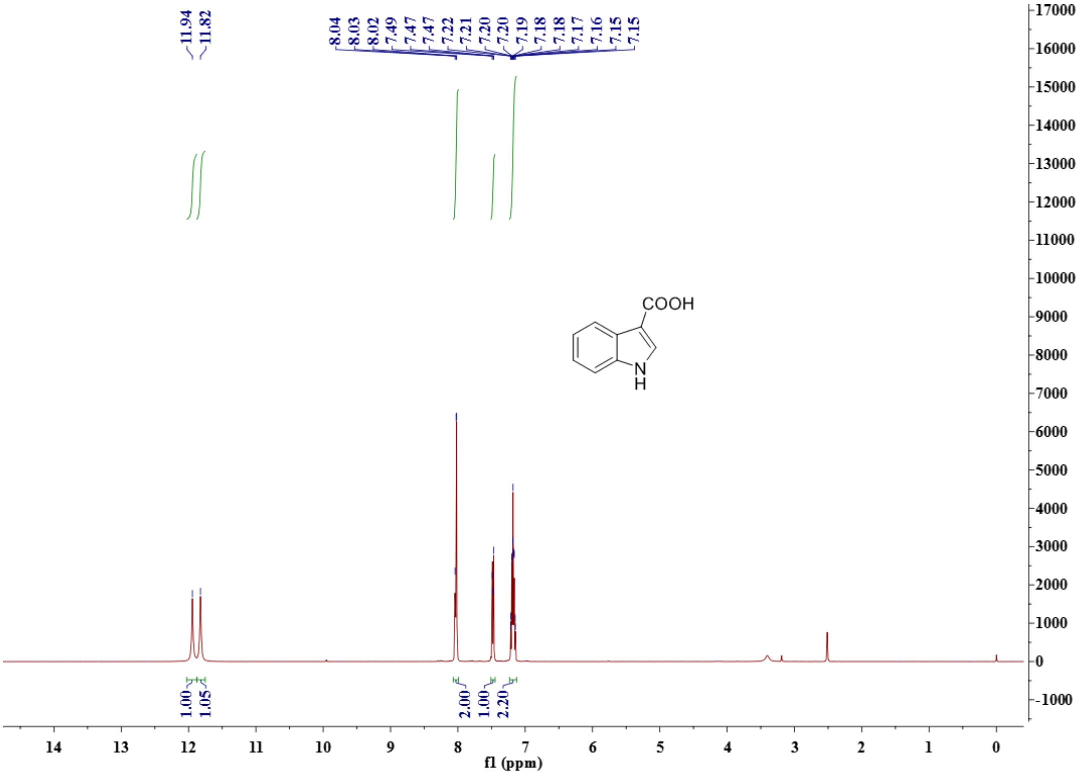

Figure S23. ^{1}H NMR spectrum of I3CA (400 MHz, DMSO-d_6).

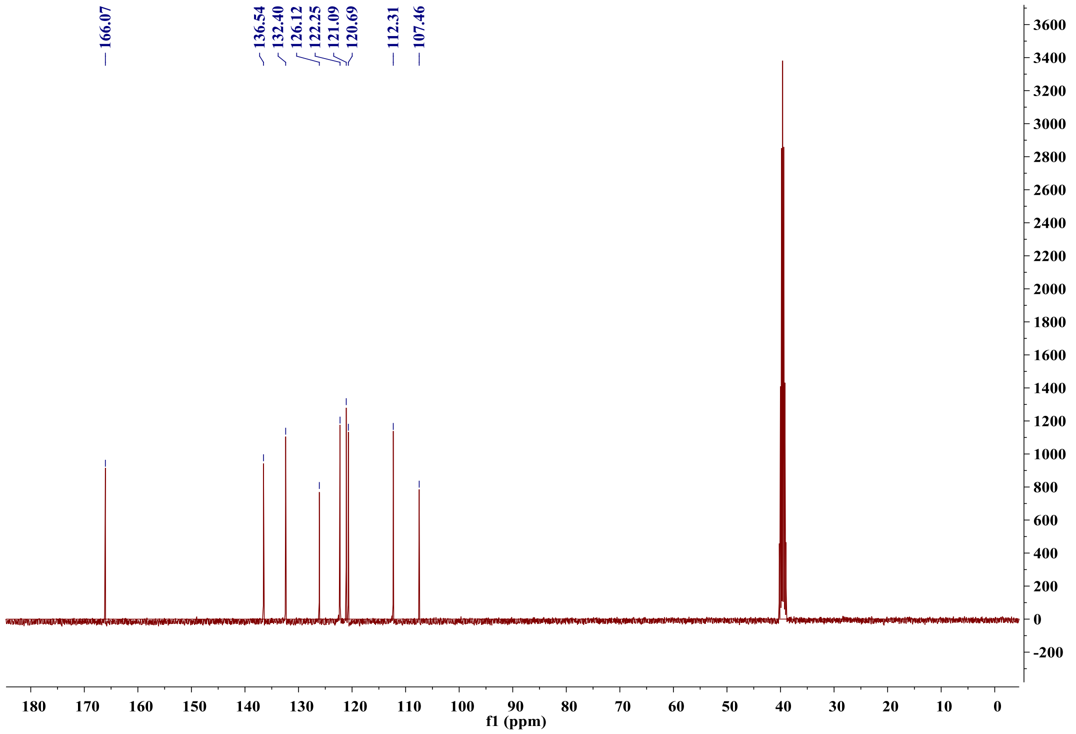

Figure S24. ^{13}C NMR spectrum of I3CA (100 MHz, DMSO-d_6).

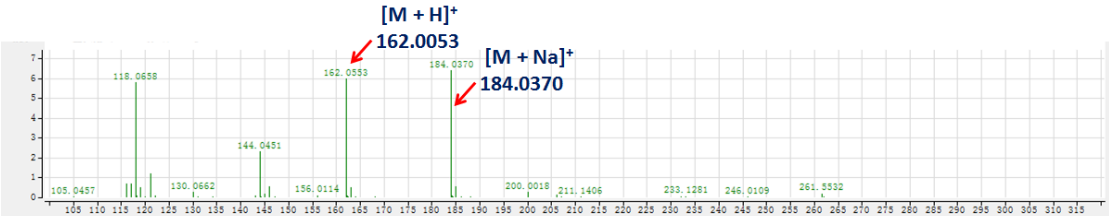

Figure S25. ESI-MS spectrum of I3CA.

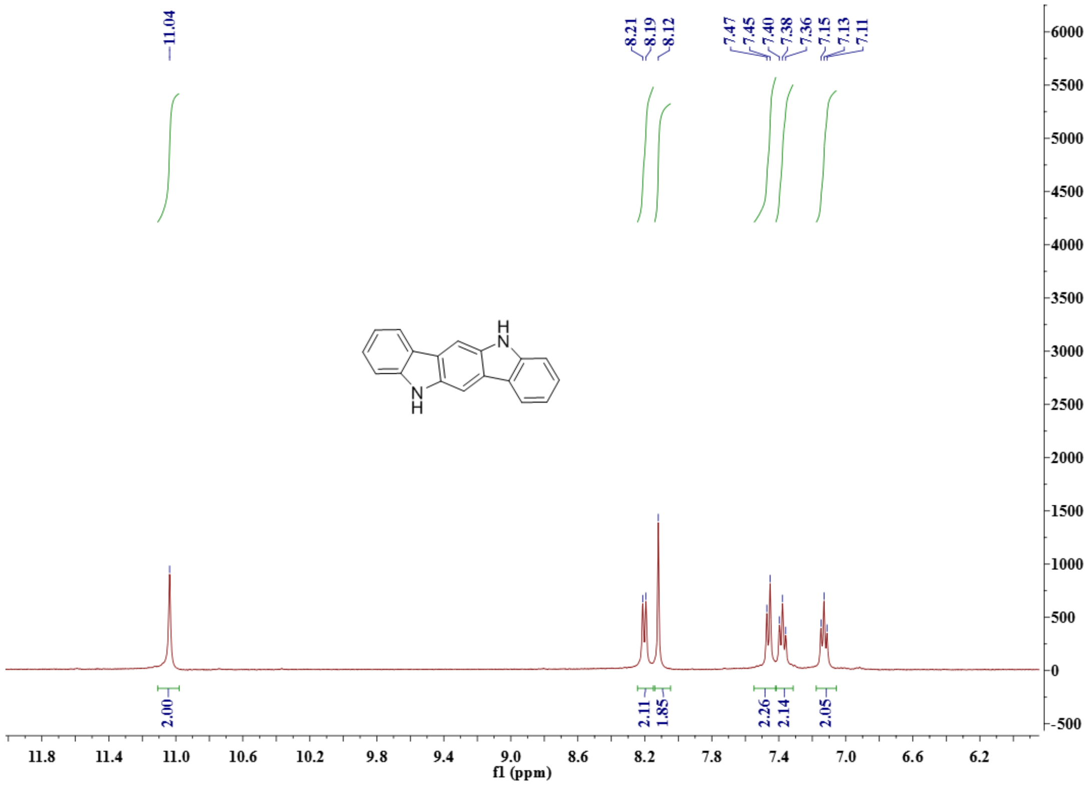

Figure S26. ^{1}H NMR spectrum of ICZ (400 MHz, DMSO-d_6).

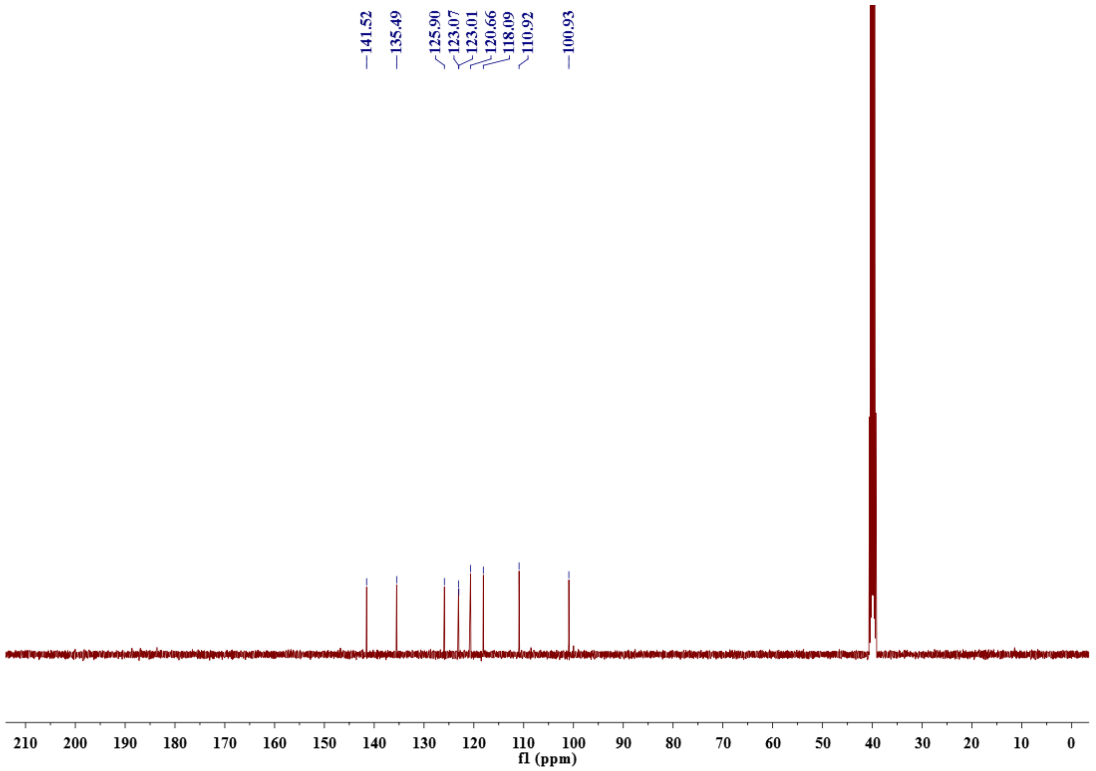

Figure S27. ^{13}C NMR spectrum of ICZ (100 MHz, DMSO-d_6).

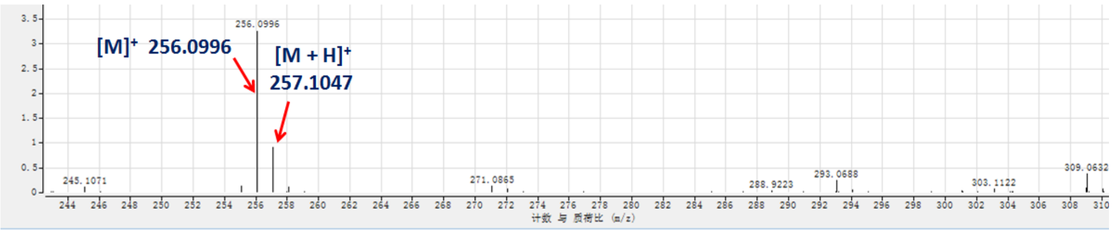

Figure S28. ESI-MS spectrum of ICZ.

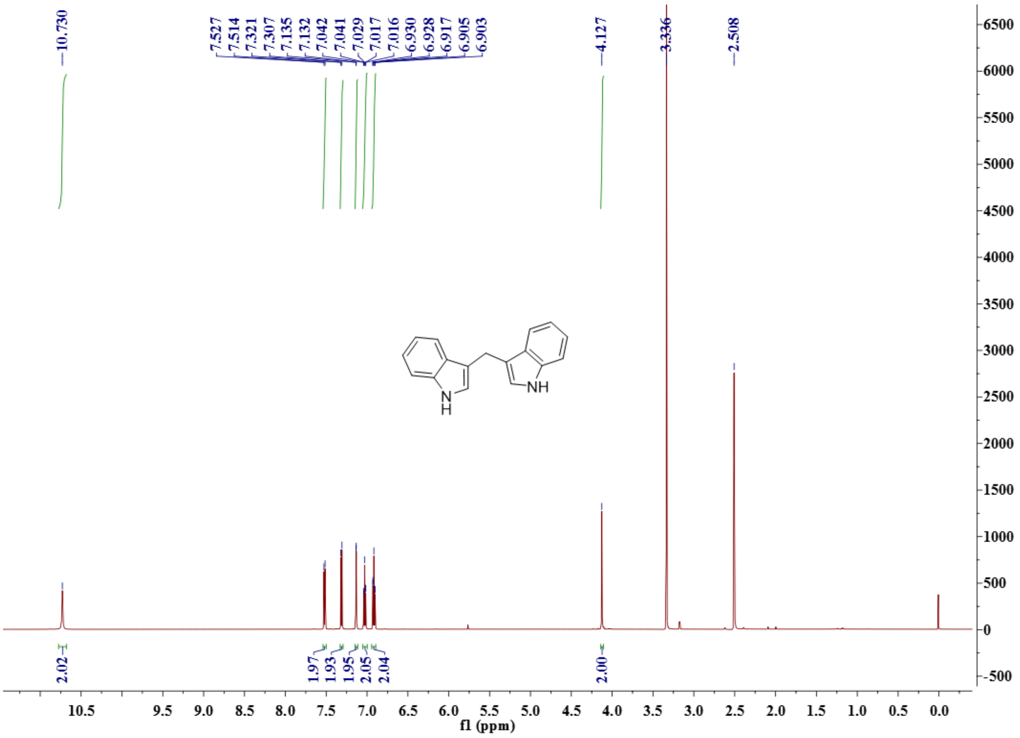

Figure S29. ^{1}H NMR spectrum of DIM (600 MHz, DMSO-d_6).

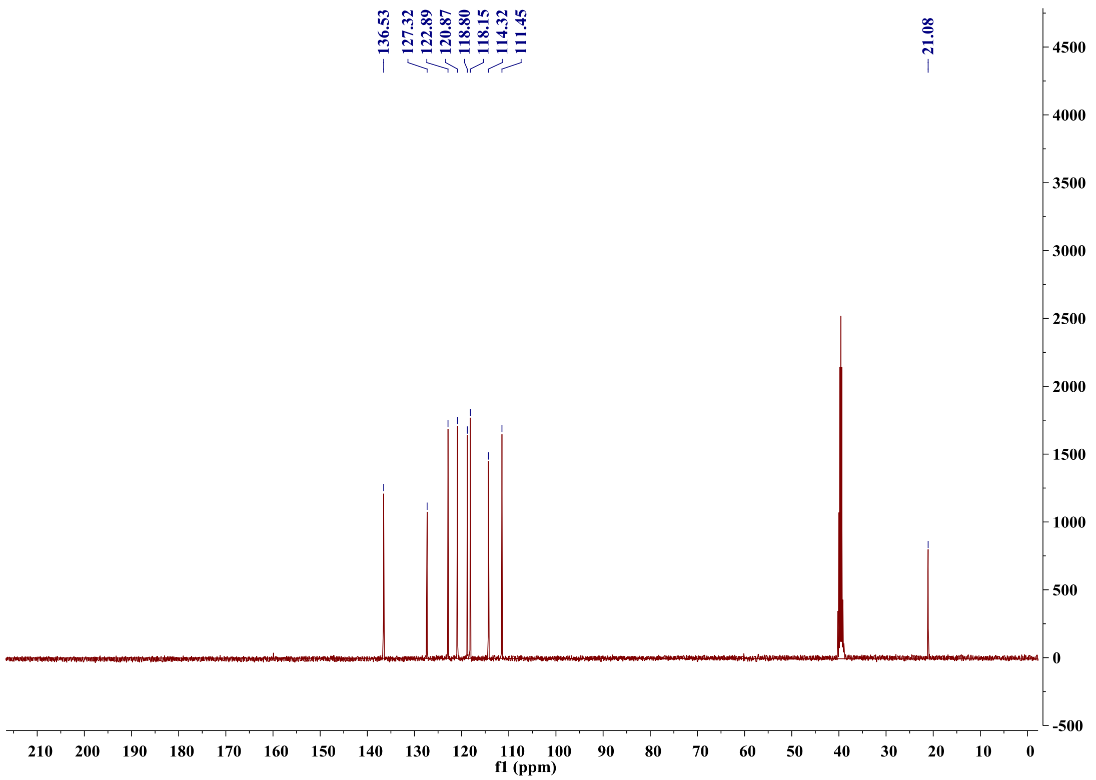

Figure S30. ^{13}C NMR spectrum of DIM (100 MHz, DMSO-d_6).

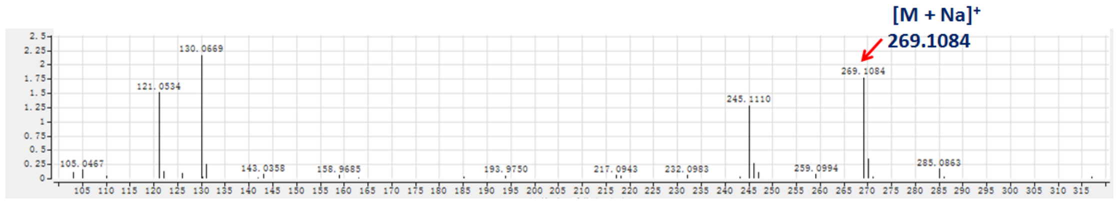

Figure S31. ESI-MS spectrum of DIM.

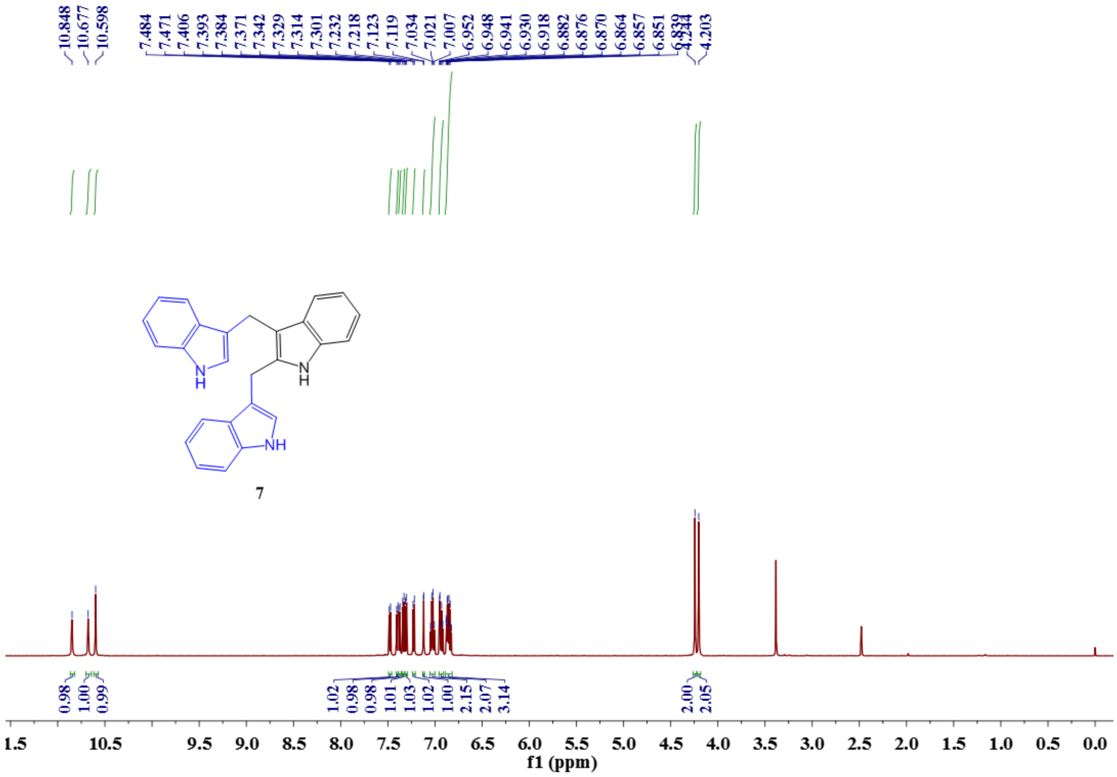

Figure S32. The ^{1}H NMR spectrum of LTr1 (600 MHz, DMSO-d_6).

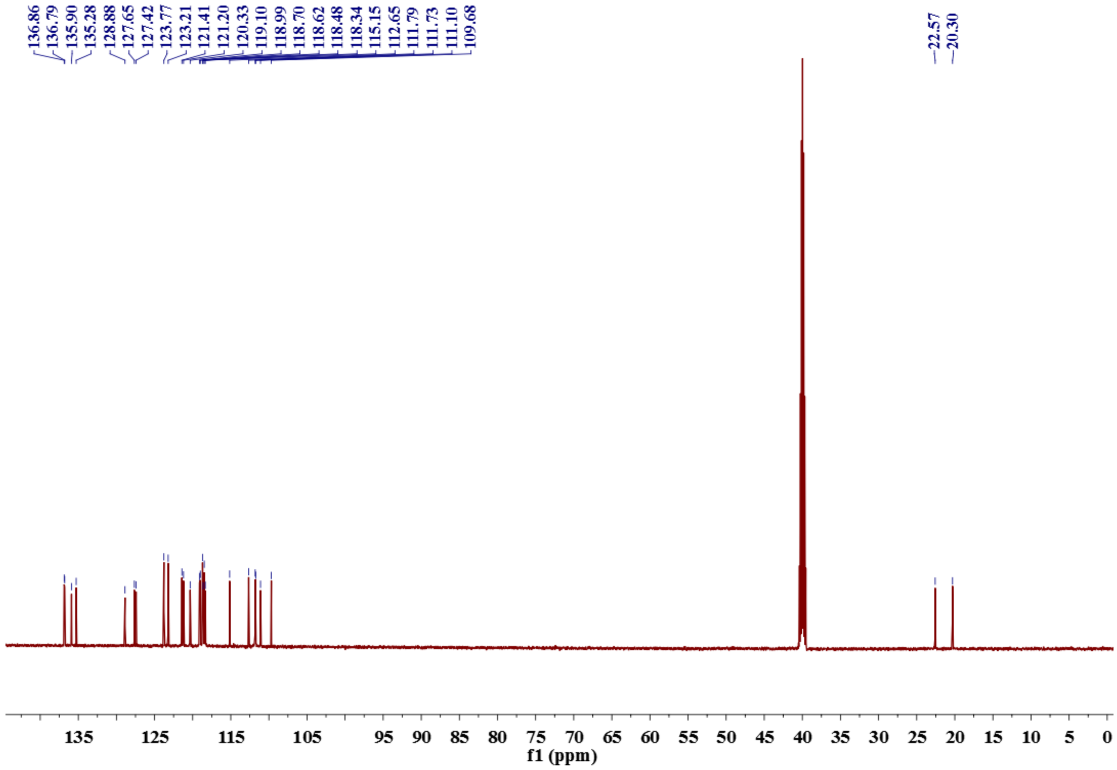

Figure S33. The ^{13}C NMR spectrum of LTr1 (100 MHz, acetone-d_6).

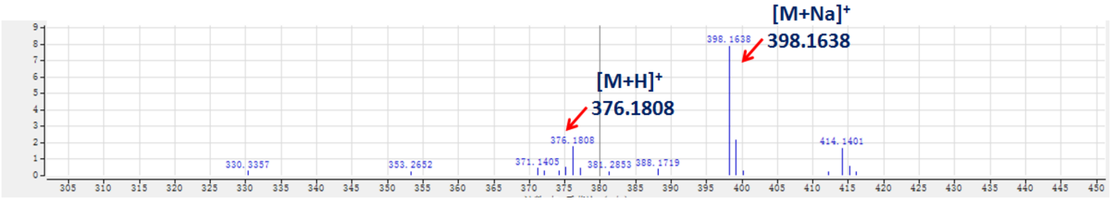

Figure S34. ESI-MS spectrum of LTr1.

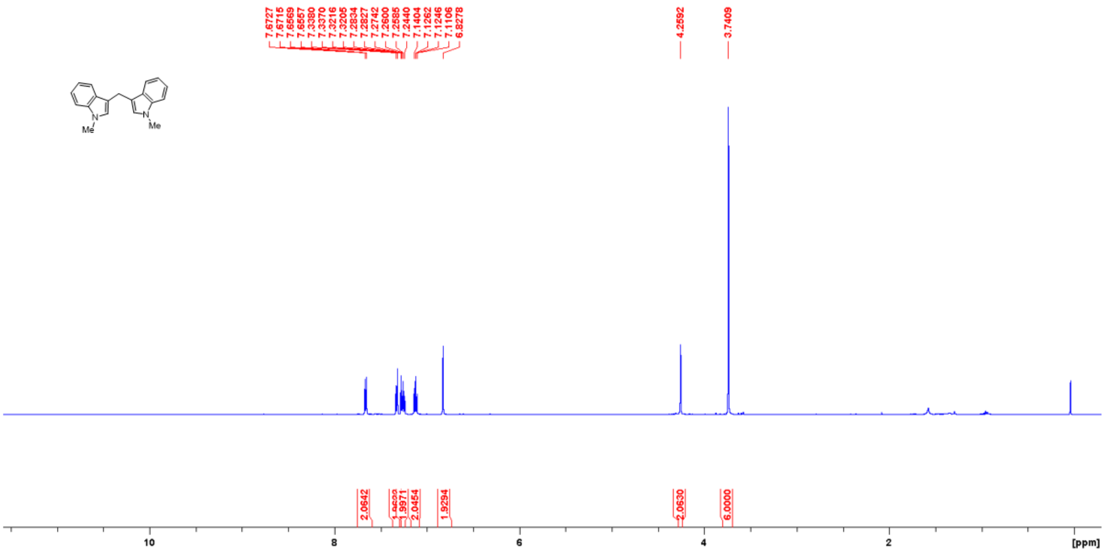

Figure S35. ^{1}H NMR spectrum of N,N'-diMe-DIM (500 MHz, acetone-d_6).

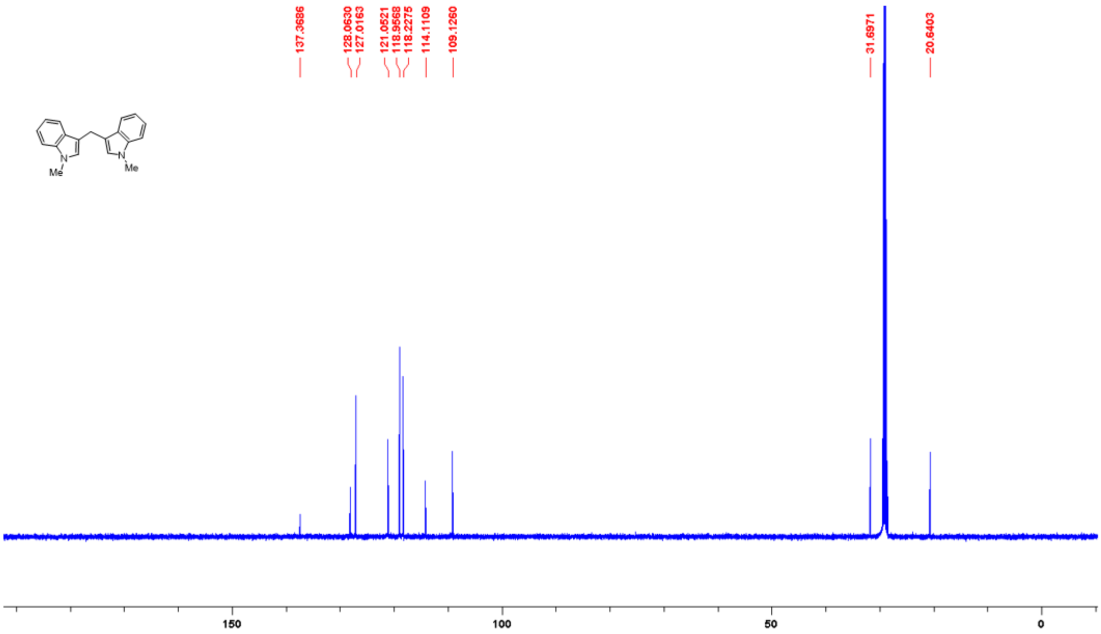

Figure S36^{13}C NMR spectrum of N,N'-diMe-DIM (125 MHz, acetone-d_6).

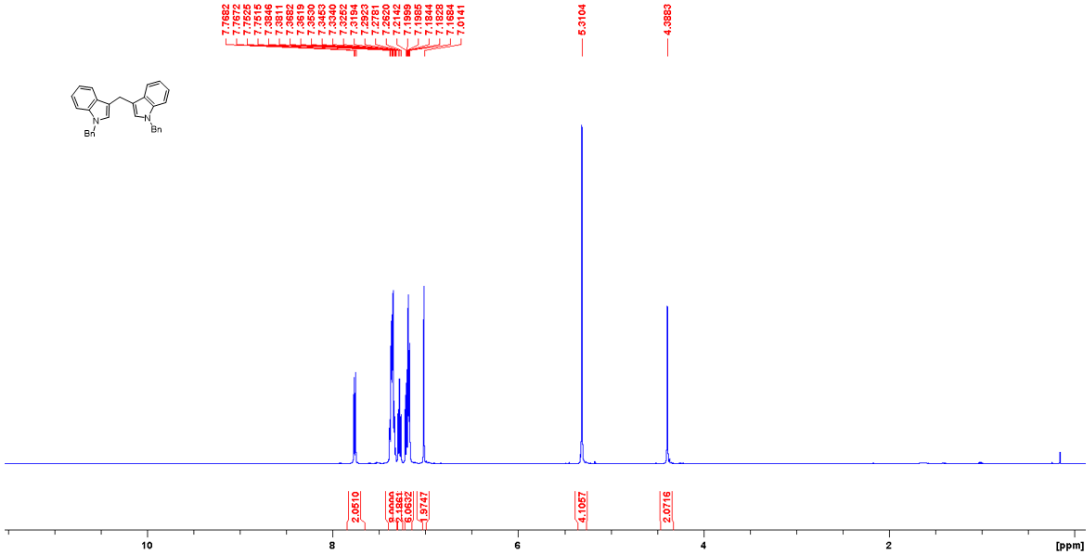

Figure S37. ^{1}H NMR spectrum of N,N'-diBn-DIM (500 MHz, acetone-d_6).

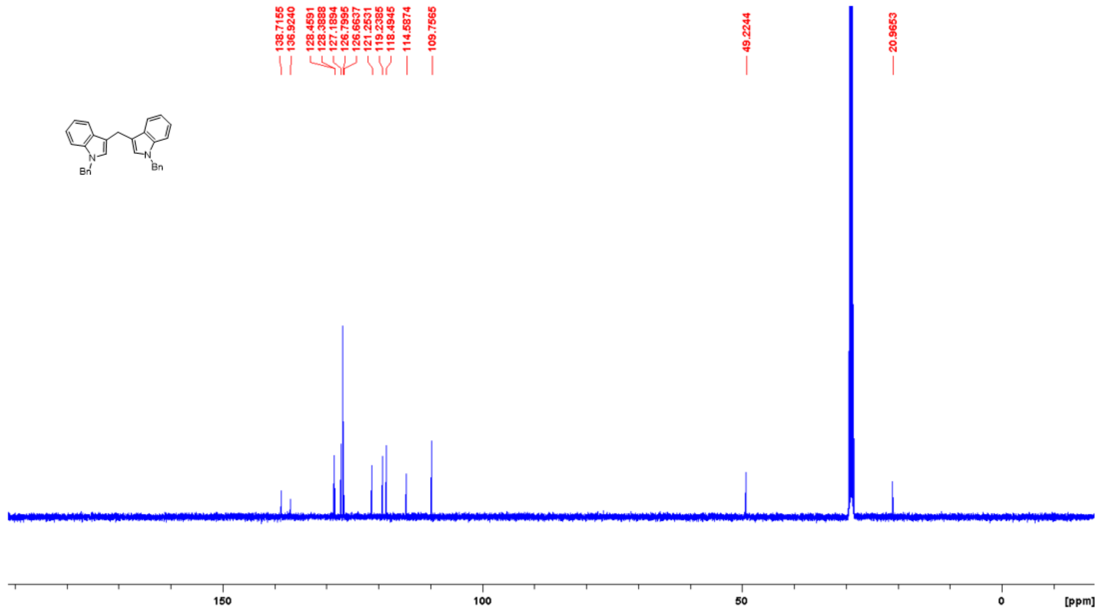

Figure S38. ^{13}C NMR spectrum of N,N'-diBn-DIM (125 MHz, acetone-d_6).

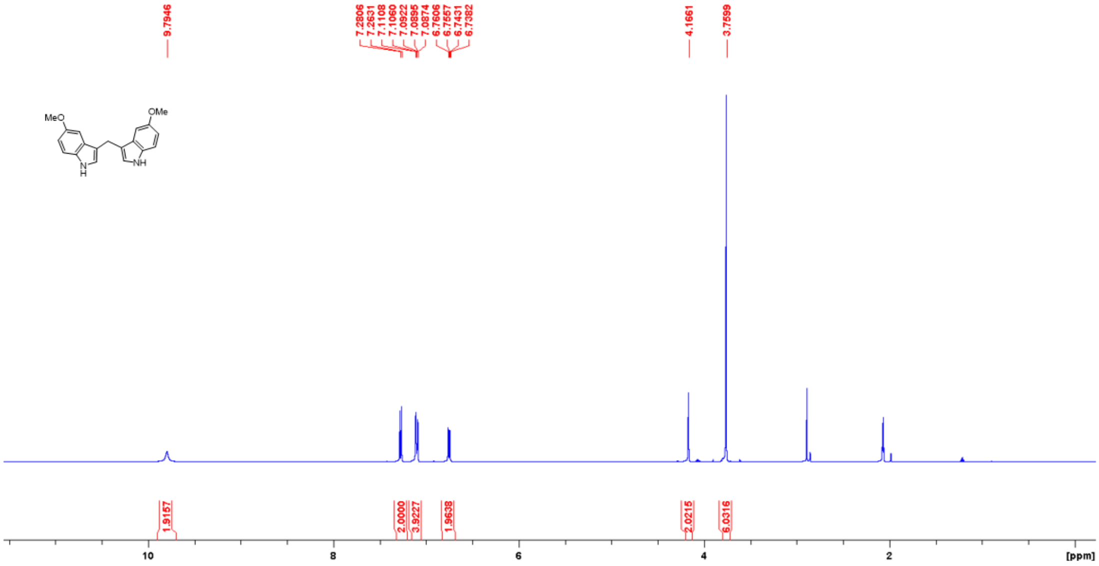

Figure S39. ^{1}H NMR spectrum of 5,5'-diMeO-DIM (500 MHz, acetone-d_6).

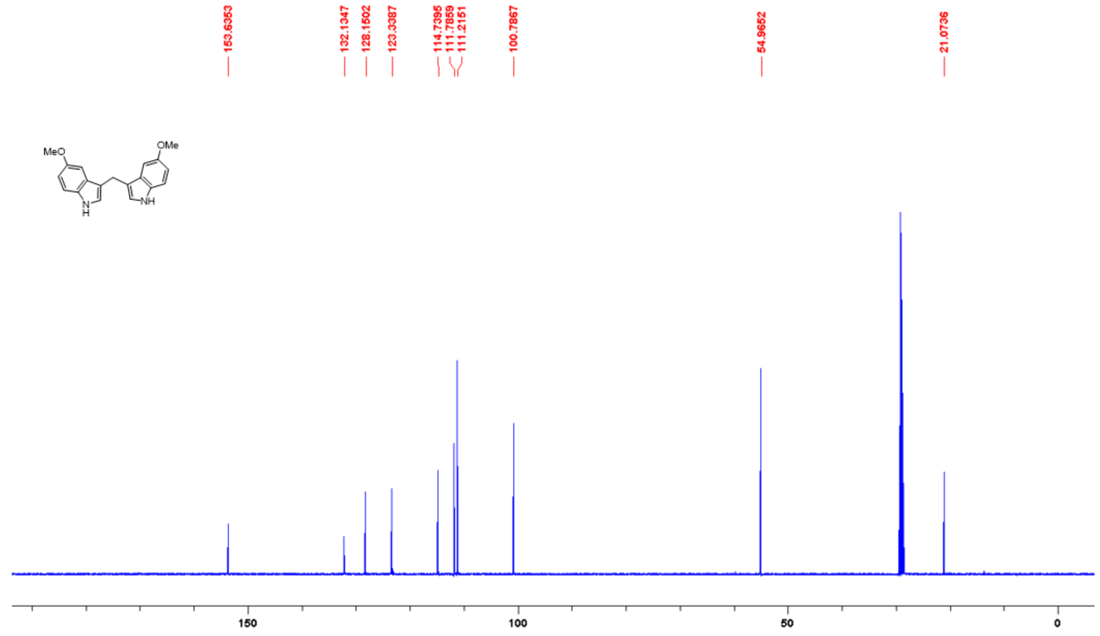

Figure S40. ^{13}C NMR spectrum of 5,5'-diMeO-DIM (125 MHz, acetone-d_6).

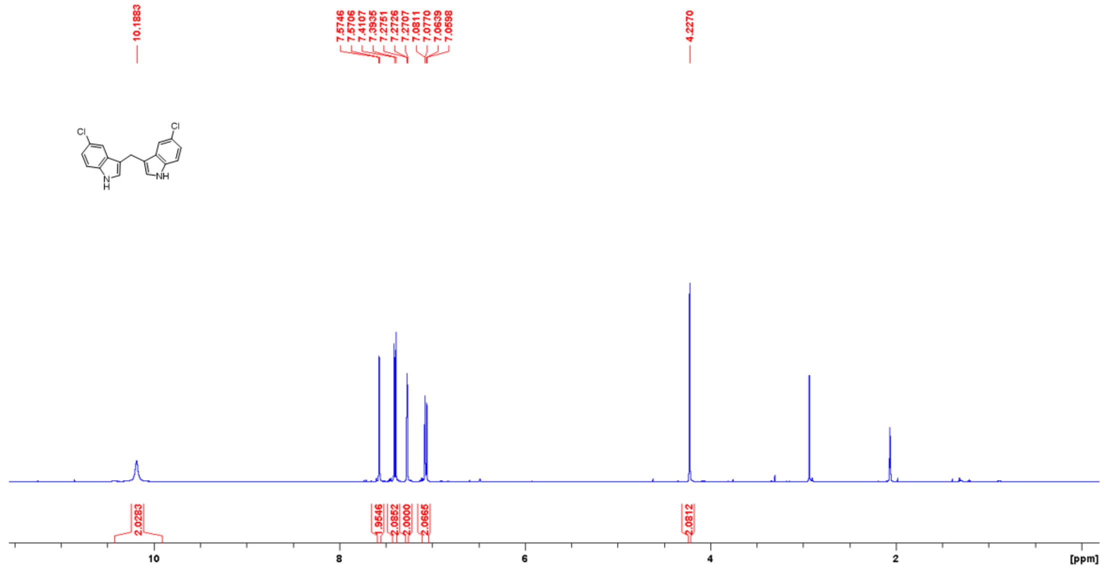

Figure S41. ^{1}H NMR spectrum of 5,5'-diCl-DIM (500 MHz, acetone-d_6).

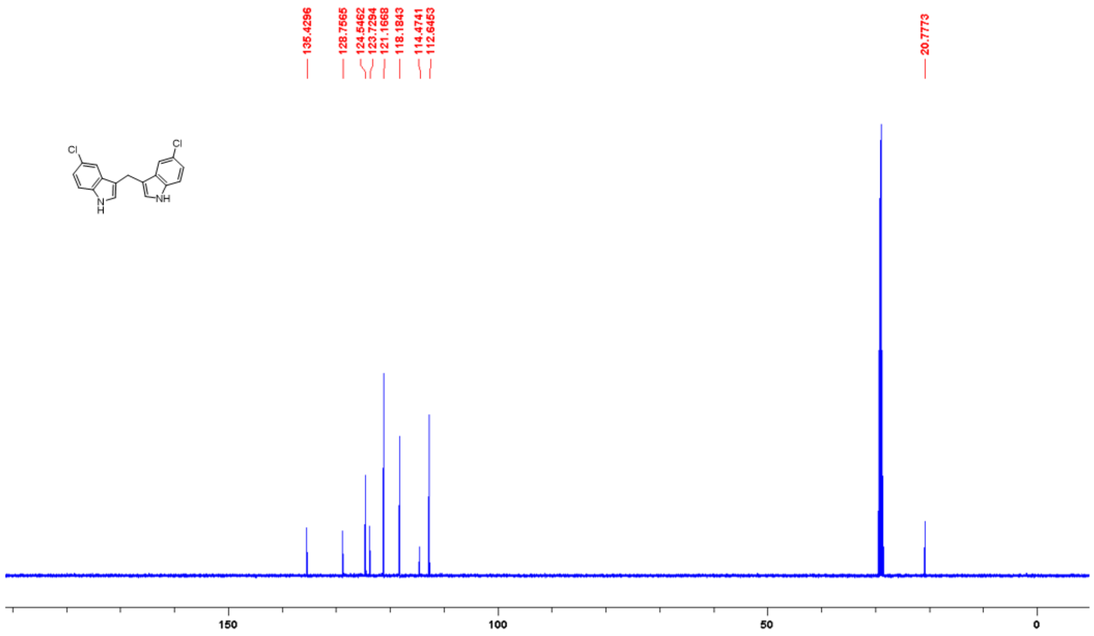

Figure S42. ^{13}C NMR spectrum of 5,5'-diCl-DIM (125 MHz, acetone-d_6).

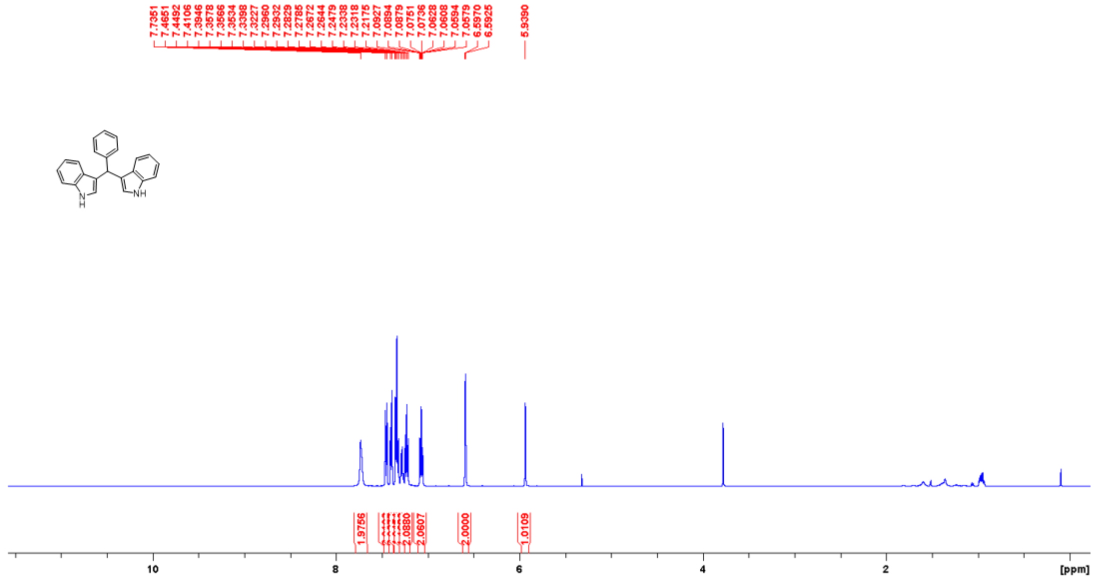

Figure S43. ^{1}H NMR spectrum of 8-Ph-DIM (500 MHz, $CDCl_3$).

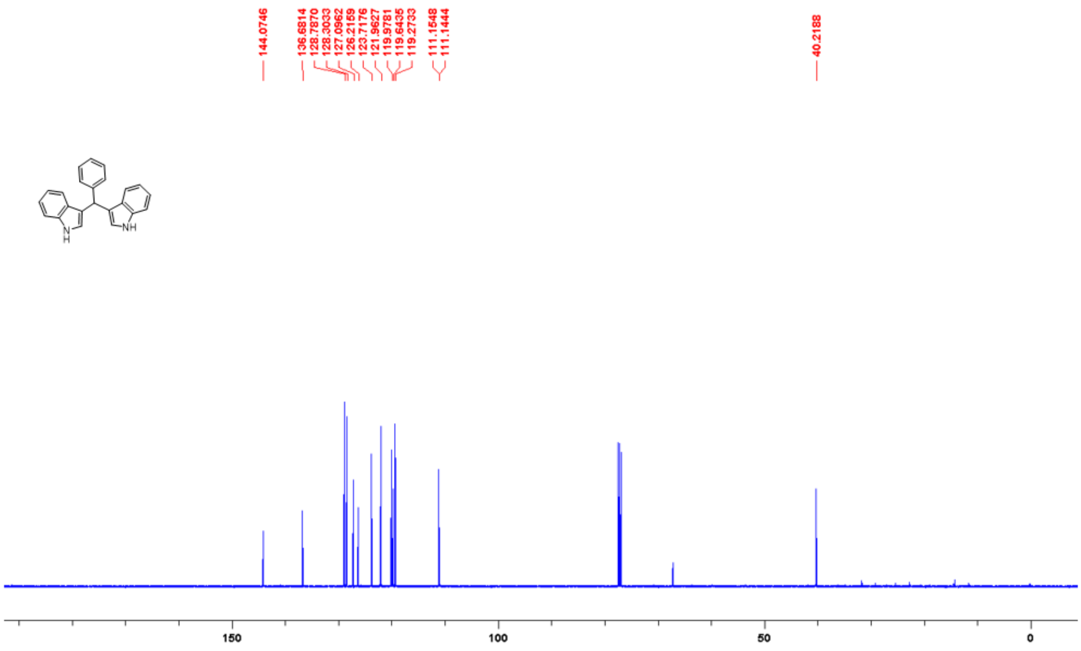

Figure S44. ^{13}C NMR spectrum of 8-Ph-DIM (125 MHz, $CDCl_3$).

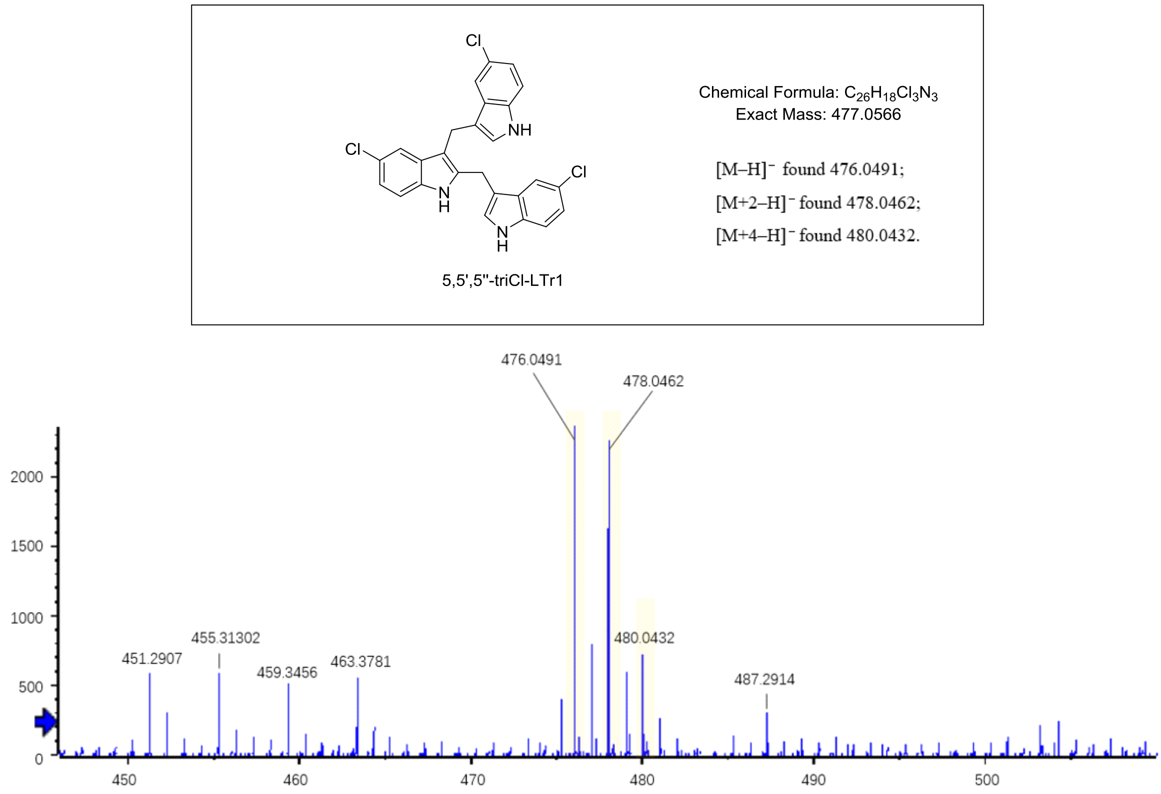

Figure S45. ESI-MS spectrum of 5,5’,5’’-triCl-LTr1.

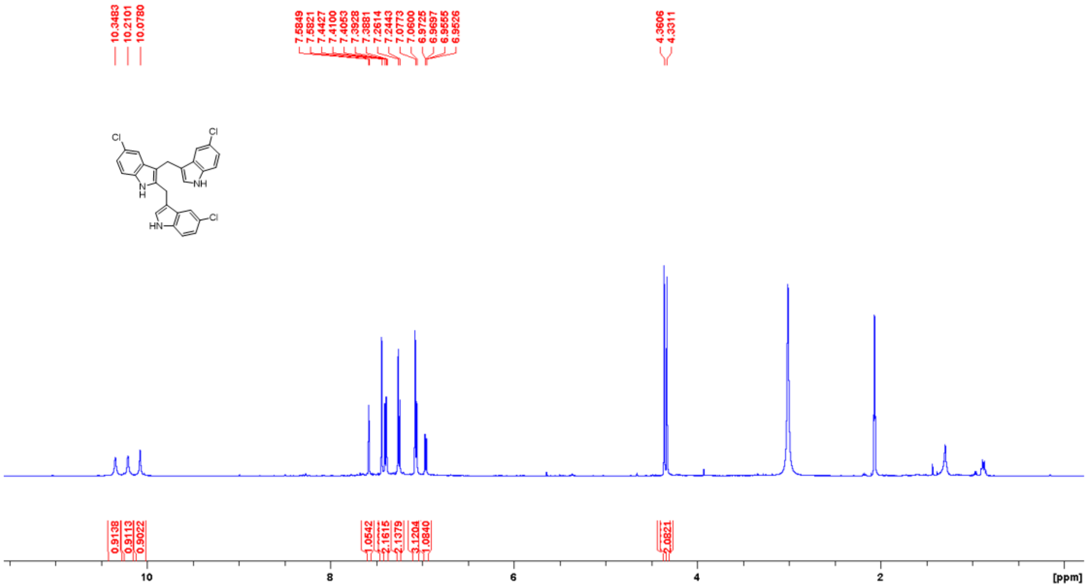

Figure S46. ^{1}H NMR spectrum of 5,5’,5’’-triCl-LTr1 (500 MHz, acetone-d_6).

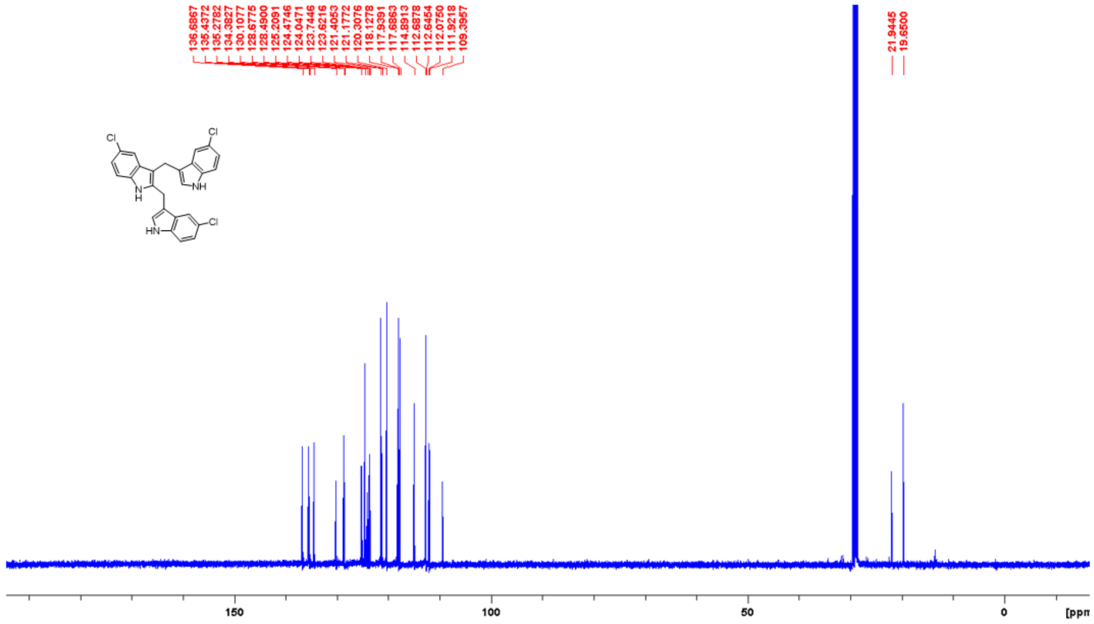

Figure S47. ^{13}C NMR spectrum of 5,5’,5’’-triCl-LTr1 (125 MHz, acetone-d_6).

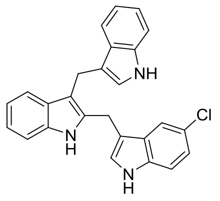

5"-Cl-LTr1

Chemical Formula: $C_{26}H_{20}ClN_3$
Exact Mass: 409.1346

$[M-H]^-$ found 408.1265;
$[M+2-H]^-$ found 410.1242.

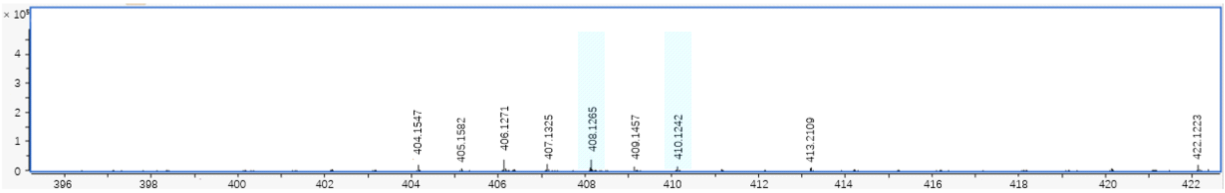

Figure S48. ESI-MS spectrum of 5”-Cl-LTr1.

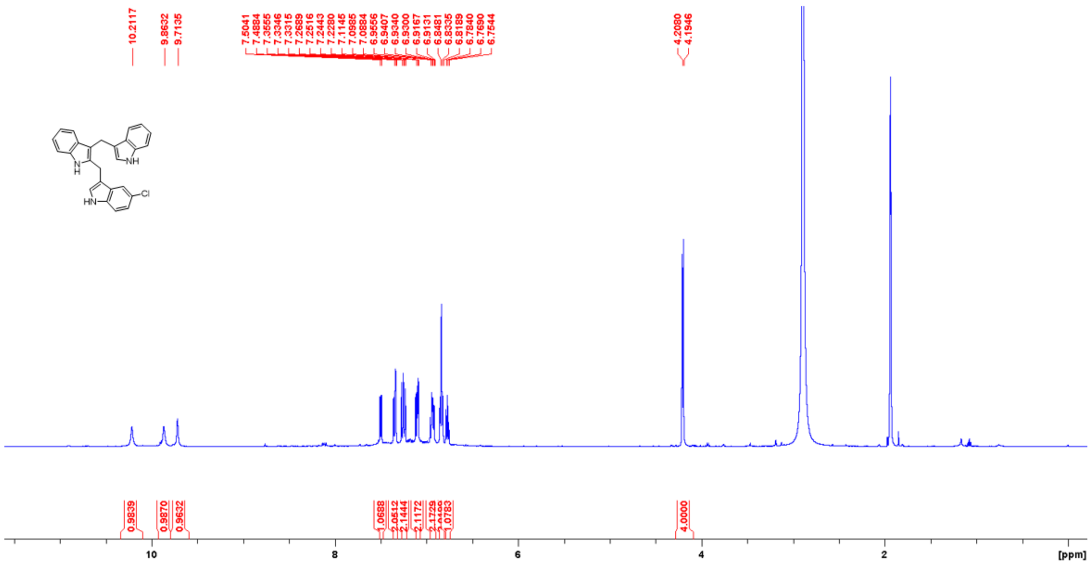

Figure S49. ^{1}H NMR spectrum of 5”-Cl-LTr1 (500 MHz, acetone-d_6).

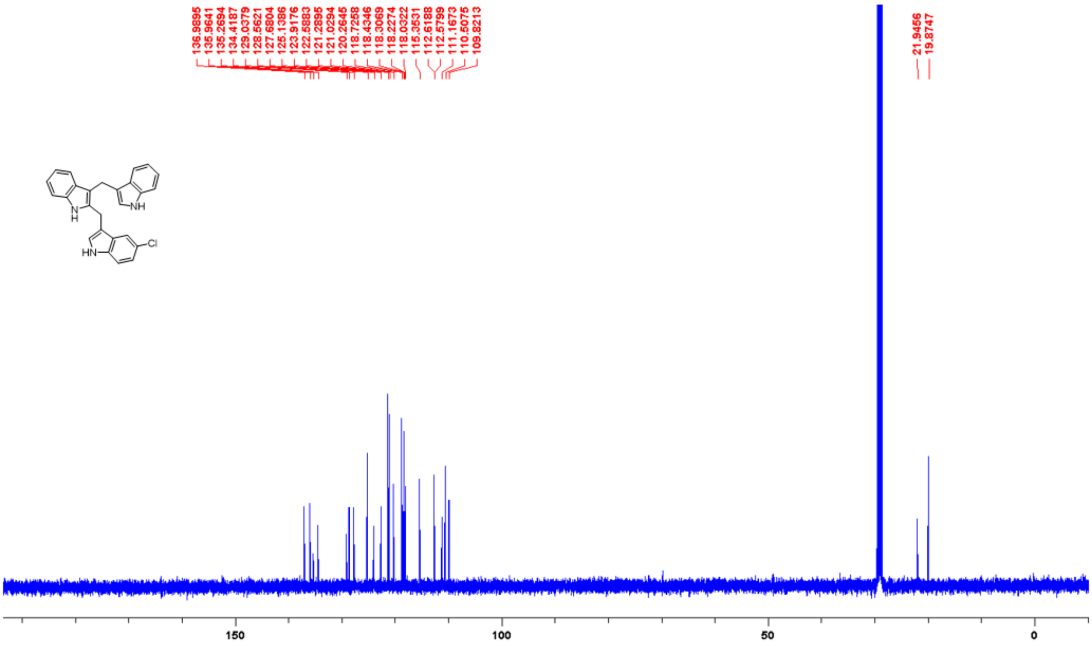

Figure S50. ^{13}C NMR spectrum of 5”-Cl-LTr1 (125 MHz, acetone-d_6).

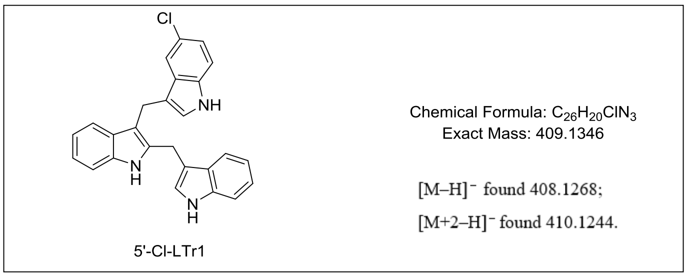

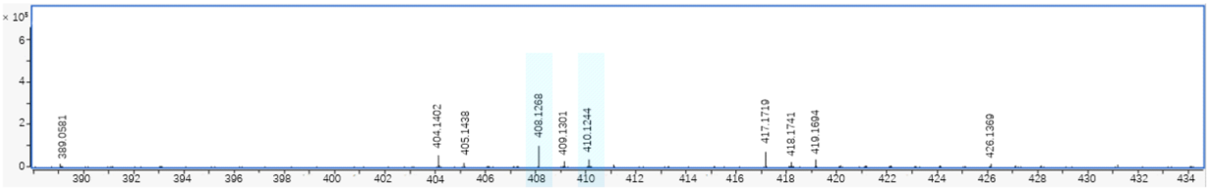

Figure S51. ESI-MS spectrum of 5’-Cl-LTr1.

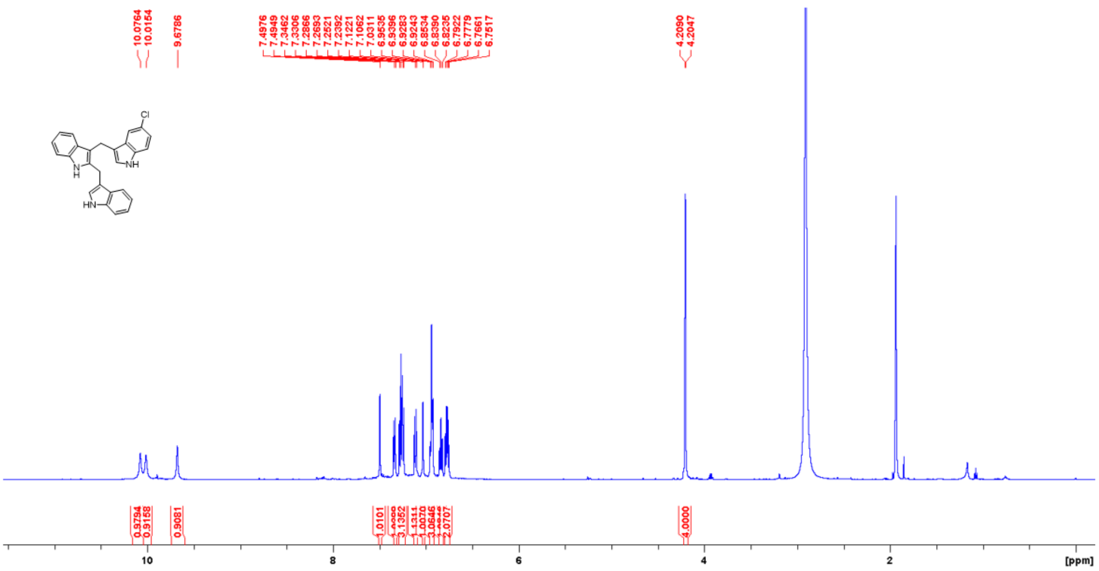

Figure S52. ^{1}H NMR spectrum of 5'-Cl-LTr1 (500 MHz, acetone-d_6).

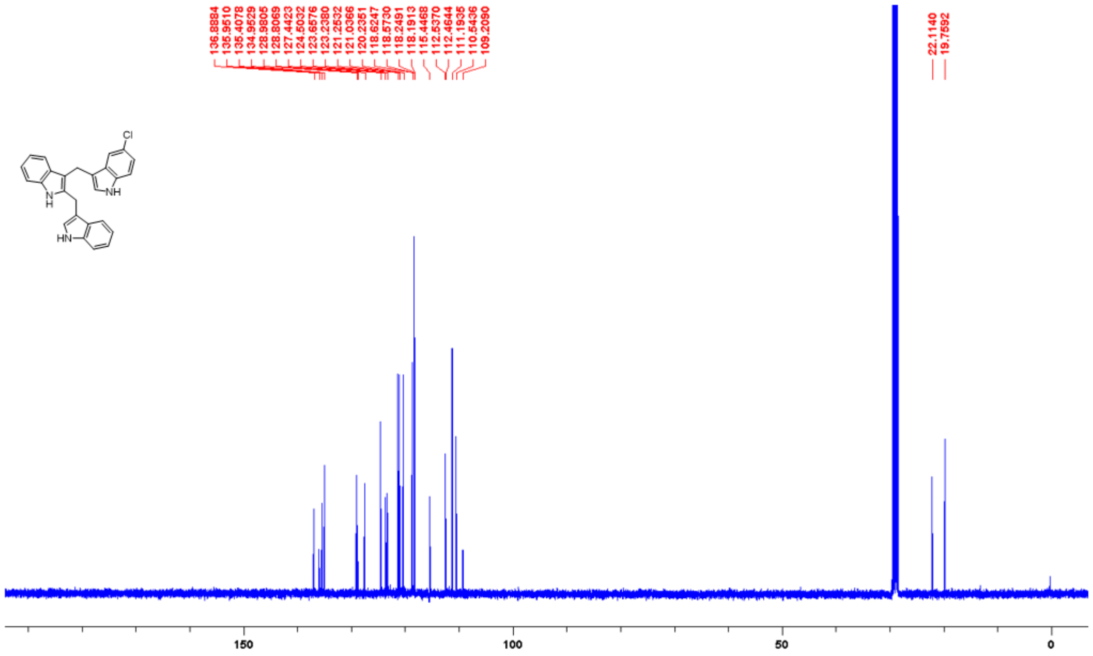

Figure 53. ^{13}C NMR spectrum of 5'-Cl-LTr1 (125 MHz, acetone-d_6).

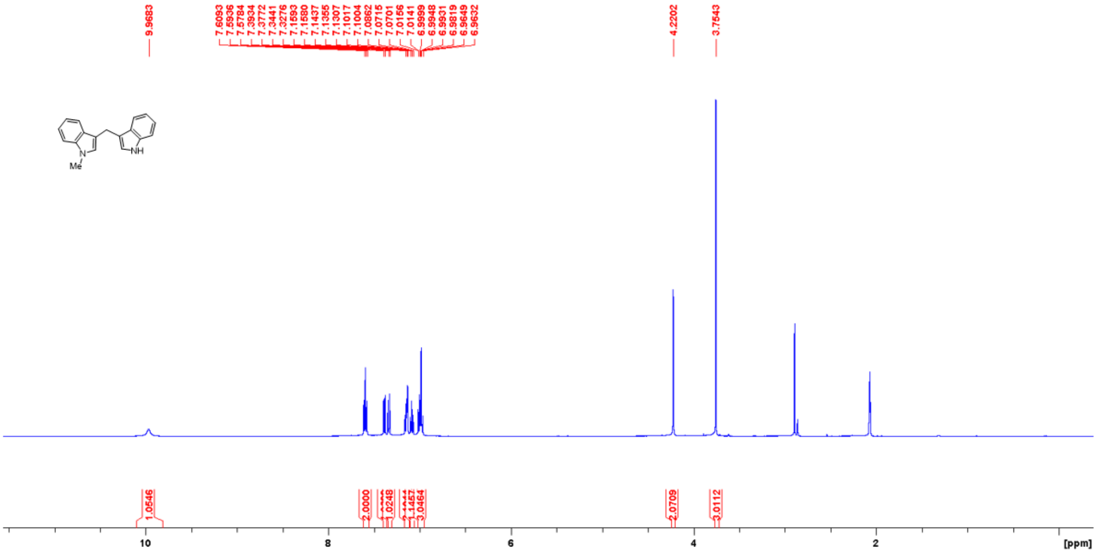

Figure S54. ^{1}H NMR spectrum of N-Me-DIM (500 MHz, acetone-d_6).

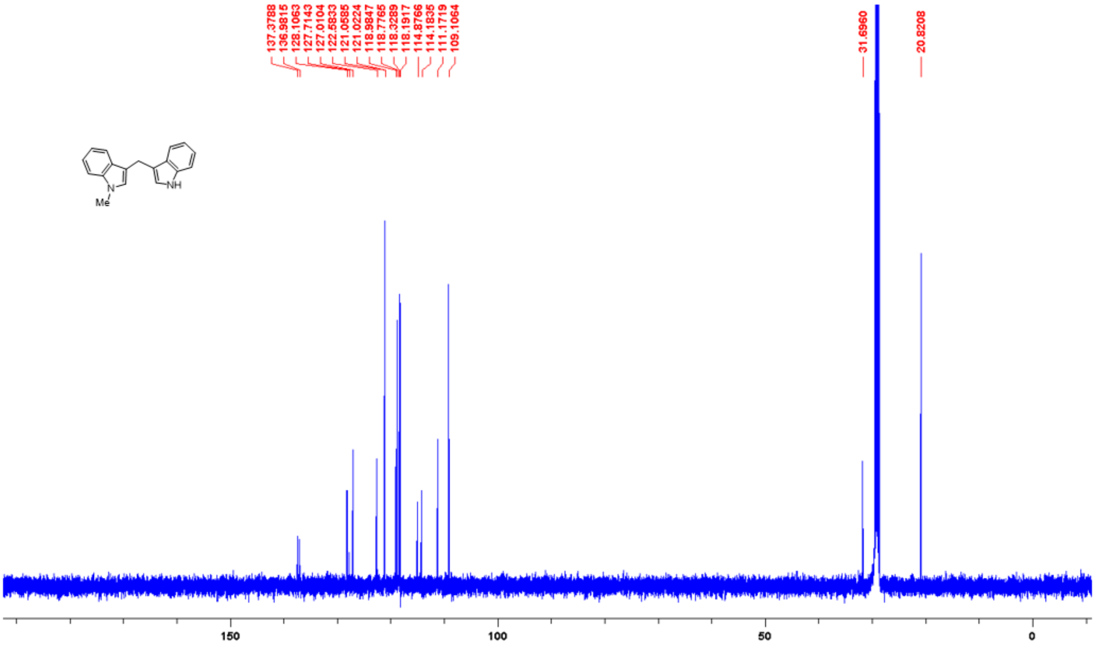

Figure S55. ^{13}C NMR spectrum of N-Me-DIM (125 MHz, acetone-d_6).

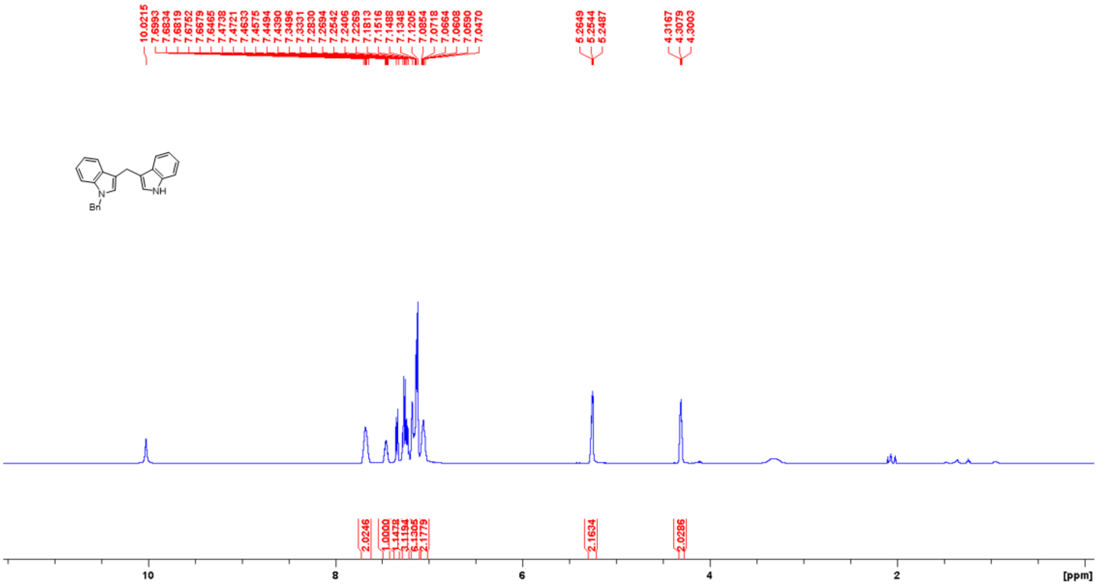

Figure S56. ^{1}H NMR spectrum of N-Bn-DIM (500 MHz, acetone-d_6).

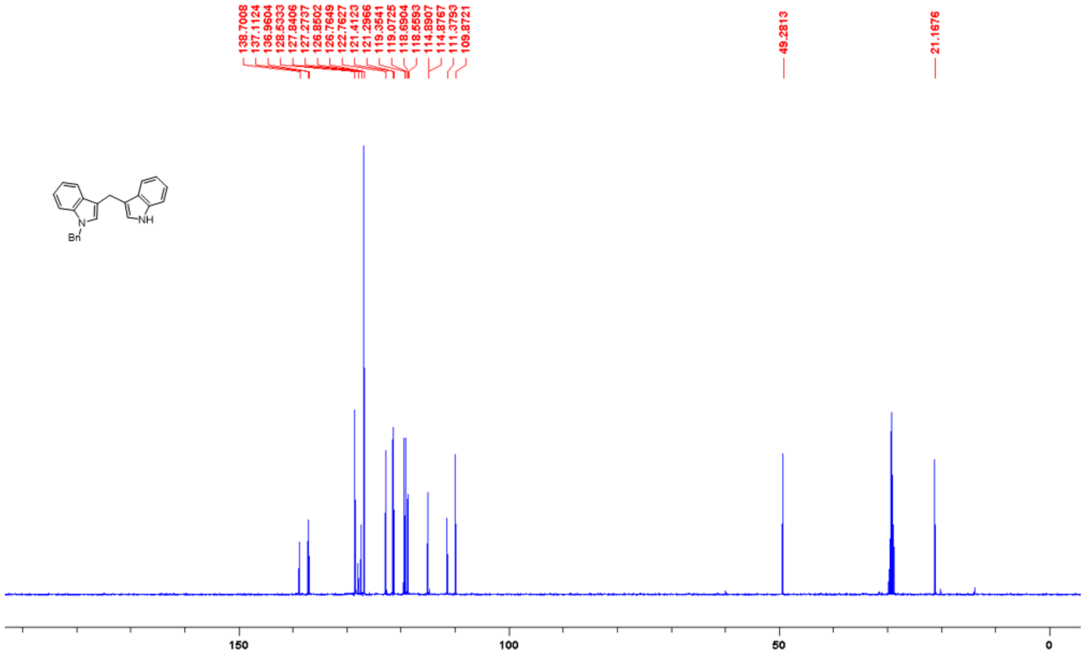

Figure S57. ^{13}C NMR spectrum of N-Bn-DIM (125 MHz, acetone-d_6).

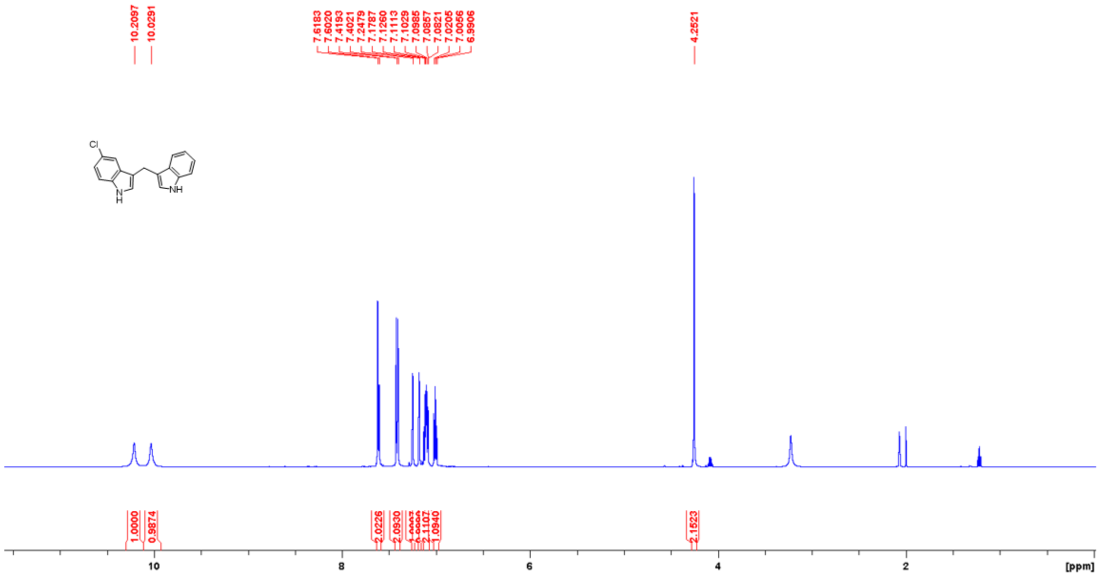

Figure S58. ^{1}H NMR spectrum of 5-Cl-DIM (500 MHz, acetone-d_6).

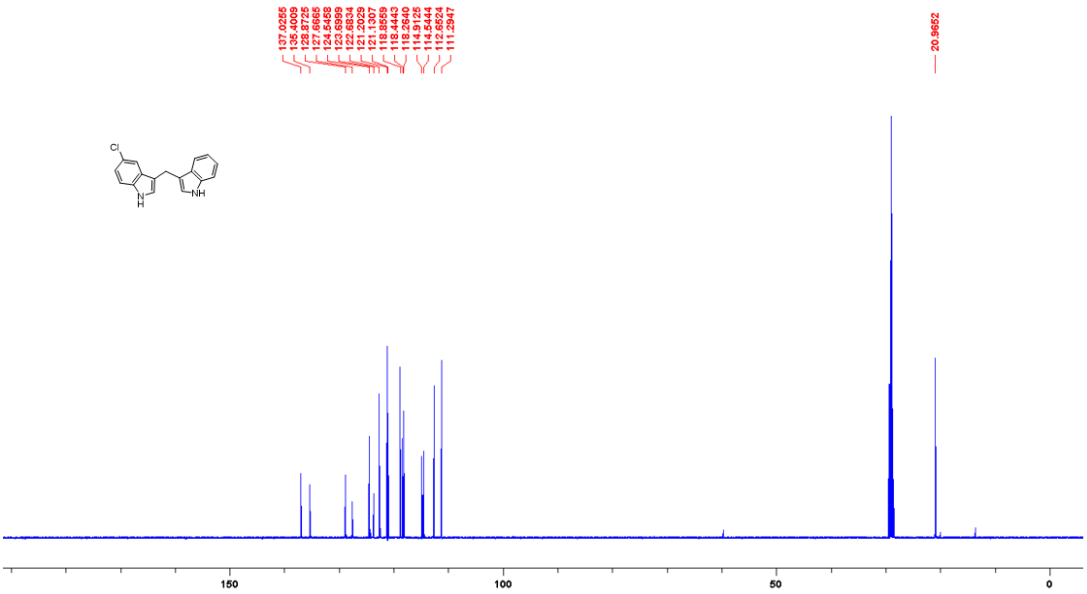

Figure S59. ^{13}C NMR spectrum of 5-Cl-DIM (125 MHz, acetone-d_6).

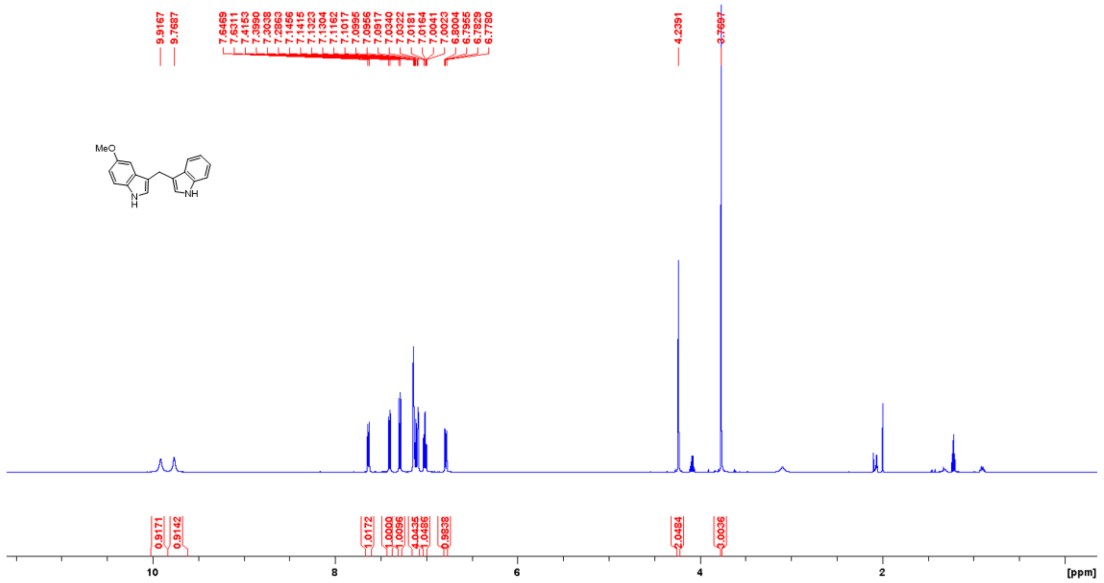

Figure S60. ^{1}H NMR spectrum of 5-MeO-DIM (500 MHz, acetone-d_6).

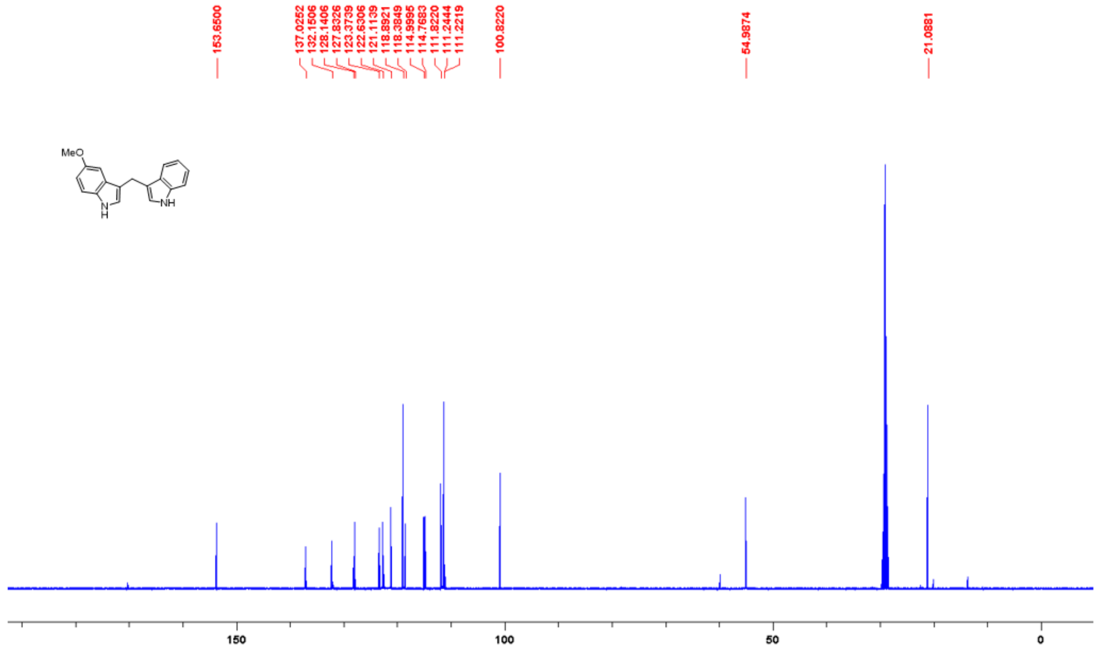

Figure S61. ^{13}C NMR spectrum of 5-MeO-DIM (125 MHz, acetone-d_6).

References

1. Vétizou M, Pitt JM and Daillère R *et al.* Anticancer immunotherapy by CTLA-4 blockade relies on the gut microbiota. *Science* 2015; **350**: 1079-84.
2. Jackson EL, Willis N and Mercer K *et al.* Analysis of lung tumor initiation and progression using conditional expression of oncogenic *K-ras*. *Genes Dev* 2001; **15**: 3243-8.
3. Kerr EM, Gaude E and Turrell FK *et al.* Mutant Kras copy number defines metabolic reprogramming and therapeutic susceptibilities. *Nature* 2016; **531**: 110-3.
4. Liu XJ, Duan NN and Liu C *et al.* Characterization of a murine nonalcoholic steatohepatitis model induced by high fat high calorie diet plus fructose and glucose in drinking water. *Lab Invest* 2018; **98**: 1184-99.
5. Hassaninasab A, Hashimoto Y and Tomita-Yokotani K *et al.* Discovery of the curcumin metabolic pathway involving a unique enzyme in an intestinal microorganism. *Proc Natl Acad Sci U S A* 2011; **108**: 6615-20.
6. Zimmermann M, Zimmermann-Kogadeeva M and Wegmann R *et al.* Mapping human microbiome drug metabolism by gut bacteria and their genes. *Nature* 2019; **570**: 462-7.
7. Cohen LJ, Esterhazy D and Kim SH *et al.* Commensal bacteria make GPCR ligands that mimic human signalling molecules. *Nature* 2017; **549**: 48-53.
8. Demir E, Li K and Bobrowski-Khoury N *et al.* The pheromone darcin drives a circuit for innate and reinforced behaviours. *Nature* 2020; **578**: 137-41.
9. Fontaine CA, Skorupski AM and Vowles CJ *et al.* How free of germs is germ-free? Detection of bacterial contamination in a germ free mouse unit. *Gut microbes* 2015; **6**: 225-33.
10. Wang W, Lin L and Du Y *et al.* Assessing the viability of transplanted gut microbiota by sequential tagging with D-amino acid-based metabolic probes. *Nat Commun* 2019; **10**: 1317.
11. Qiao Y, Sun J and Xie Z *et al.* Propensity to high-fat diet-induced obesity in mice is associated with the indigenous opportunistic bacteria on the interior of Peyer's patches. *J Clin Biochem Nutr* 2014; **55**: 120-8.
12. Panesar HK, Solano J and Minehan TG. Synthesis and DNA binding profile of N-mono- and N,N'-disubstituted indolo[3,2-b]carbazoles. *Org Biomol Chem* 2015; **13**: 2879-83.
13. Jong L, Jiang F and Li G *et al.* Analogs of indole-3-carbinol and their use as agents against infection. WO, WO2010033198 A2[P]. 2012.
14. Qiang WW, Liu X and Loh TPJASC *et al.* Supported iridium catalyst for the green synthesis of 3,3′-bis(indolyl)methanes using methanol as the bridging methylene source. *ACS Sustain Chem Eng* 2019; **7**:8429−8439.
15. Yan M, Hider R and Ma YJOCF. Cu(II)- or Co(II)-Catalyzed C(SP3)–H oxidation of N,N-dimethylaminoethanol: facile synthesis of methylene-bridged biindoles and 3-formylindoles selectively. *Org Chem Front* 2019; **6**:1168-1172.

16. Li D, Wu T and Liang K *et al.* Curtius-like rearrangement of an iron-nitrenoid complex and application in biomimetic synthesis of bisindolylmethanes. *Org Lett* 2016;18: 2228-31.
17. Sung WS and Lee DG. Mechanism of decreased susceptibility for Gram-negative bacteria and synergistic effect with ampicillin of indole-3-carbinol. *Biol Pharm Bull* 2008; **31**: 1798-801.
18. Aggarwal BB and Ichikawa H. Molecular targets and anticancer potential of indole-3-carbinol and its derivatives. *Cell Cycle* 2005; **4**: 1201-15.
19. Lee YR, Chen M and Lee JD *et al.* Reactivation of PTEN tumor suppressor for cancer treatment through inhibition of a MYC-WWP1 inhibitory pathway. *Science* 2019; **364**: eaau0159.
20. Wu Y, Li RW and Huang H *et al.* Inhibition of tumor growth by dietary indole-3-carbinol in a prostate cancer xenograft model may be associated with disrupted gut microbial interactions. *Nutrients* 2019; **11**:467.
21. Adwas AA, Elkhoely AA and Kabel AM *et al.* Anti-cancer and cardioprotective effects of indol-3-carbinol in doxorubicin-treated mice. *J Infect Chemother* 2016; **22**: 36-43.
22. Arora A and Shukla Y. Modulation of vinca-alkaloid induced P-glycoprotein expression by indole-3-carbinol. *Cancer Lett* 2003; **189**: 167-73.
23. Manson MM, Hudson EA and Ball HW *et al.* Chemoprevention of aflatoxin B1-induced carcinogenesis by indole-3-carbinol in rat liver--predicting the outcome using early biomarkers. *Carcinogenesis* 1998; **19**: 1829-36.
24. Hsu JC, Zhang J and Dev A *et al.* Indole-3-carbinol inhibition of androgen receptor expression and downregulation of androgen responsiveness in human prostate cancer cells. *Carcinogenesis* 2005; **26**: 1896-904.
25. Subhan H, Ahmad K and Lashin A *et al.* pH and temperature responsive electrooxidation and antioxidant activity of indole-3-carbaldehyde. *J Electrochem Soc* 2016; **163**: H690-H6.
26. Rajalaxmi M, Beema Shafreen R and Iyer PM *et al.* An *in silico*, *in vitro* and *in vivo* investigation of indole-3-carboxaldehyde identified from the seawater bacterium *Marinomonas* sp. as an anti-biofilm agent against *Vibrio cholerae* O1. *Biofouling* 2016; **32**: 1-12.
27. Suzuki M, Chozin MA and Iwasaki A *et al.* Phytotoxic activity of Chinese violet (*Asystasia gangetica* (L.) T. Anderson) and two phytotoxic substances. *Weed Biol Manag* 2019; **19**:12170.
28. Zelante T, Iannitti RG and Cunha C *et al.* Tryptophan catabolites from microbiota engage aryl hydrocarbon receptor and balance mucosal reactivity via interleukin-22. *Immunity* 2013; **39**: 372-85.
29. Cano A, Alcaraz O and Arnao MB. Free radical-scavenging activity of indolic compounds in aqueous and ethanolic media. *Anal Bioanal Chem* 2003; **376**: 33-7.
30. Gamir J, Pastor V and Sánchez-Bel P *et al.* Starch degradation, abscisic acid and vesicular trafficking are important elements in callose priming by

indole-3-carboxylic acid in response to *Plectosphaerella cucumerina* infection. *Plant J* 2018; **96**: 518-31.
31. Liu H, Wormke M and Safe SH *et al.* Indolo[3,2-b]carbazole: a dietary-derived factor that exhibits both antiestrogenic and estrogenic activity. *J Natl Cancer Inst* 1994; **86**: 1758-65.
32. Bjeldanes LF, Kim JY and Grose KR *et al.* Aromatic hydrocarbon responsiveness-receptor agonists generated from indole-3-carbinol in vitro and in vivo: comparisons with 2,3,7,8-tetrachlorodibenzo-p-dioxin. *Proc Natl Acad Sci U S A* 1991; **88**: 9543-7.
33. Herrmann S, Seidelin M and Bisgaard HC *et al.* Indolo[3,2-b]carbazole inhibits gap junctional intercellular communication in rat primary hepatocytes and acts as a potential tumor promoter. *Carcinogenesis* 2002; **23**: 1861-8.
34. Xue L, Pestka JJ and Li M *et al.* 3,3'-Diindolylmethane stimulates murine immune function *in vitro* and *in vivo*. *J Nutr Biochem* 2008; **19**: 336-44.
35. Banerjee S, Kong D and Wang Z *et al.* Attenuation of multi-targeted proliferation-linked signaling by 3,3'-diindolylmethane (DIM): from bench to clinic. *Mutat Res* 2011; **728**: 47-66.
36. Choi KM and Yoo HS. 3,3'-Diindolylmethane enhances glucose uptake through activation of insulin signaling in 3T3-L1 adipocytes. *Obesity (Silver Spring, Md)* 2018; **26**: 1153-60.
37. Kim JY, Le TAN and Lee SY *et al.* 3,3'-Diindolylmethane improves intestinal permeability dysfunction in cultured human intestinal cells and the model animal *Caenorhabditis elegans*. *J Agric Food Chem* 2019; **67**: 9277-85.
38. Kong D, Li Y and Wang Z *et al.* Inhibition of angiogenesis and invasion by 3,3'-diindolylmethane is mediated by the nuclear factor-kappaB downstream target genes MMP-9 and uPA that regulated bioavailability of vascular endothelial growth factor in prostate cancer. *Cancer Res* 2007; **67**: 3310-9.
39. Vivar OI, Saunier EF and Leitman DC *et al.* Selective activation of estrogen receptor-beta target genes by 3,3'-diindolylmethane. *Endocrinology* 2010; **151**: 1662-7.
40. Leong H, Firestone GL and Bjeldanes LF. Cytostatic effects of 3,3'-diindolylmethane in human endometrial cancer cells result from an estrogen receptor-mediated increase in transforming growth factor-alpha expression. *Carcinogenesis* 2001; **22**: 1809-17.
41. Abdelrahim M, Newman K and Vanderlaag K *et al.* 3,3'-Diindolylmethane (DIM) and its derivatives induce apoptosis in pancreatic cancer cells through endoplasmic reticulum stress-dependent upregulation of DR5. *Carcinogenesis* 2006; **27**: 717-28.
42. Chang YC, Riby J and Chang GH *et al.* Cytostatic and antiestrogenic effects of 2-(indol-3-ylmethyl)-3,3'-diindolylmethane, a major *in vivo* product of dietary indole-3-carbinol. *Biochem Pharmacol* 1999; **58**: 825-34.
